# LudusScope: Accessible Interactive Smartphone Microscopy for Life-Science Education

**DOI:** 10.1371/journal.pone.0162602

**Published:** 2016-10-05

**Authors:** Honesty Kim, Lukas Cyrill Gerber, Daniel Chiu, Seung Ah Lee, Nate J. Cira, Sherwin Yuyang Xia, Ingmar H. Riedel-Kruse

**Affiliations:** Department of Bioengineering, Stanford University, Stanford, CA 94305, United States of America; Texas A&M University College Station, UNITED STATES

## Abstract

For centuries, observational microscopy has greatly facilitated biology education, but we still cannot easily and playfully interact with the microscopic world we see. We therefore developed the LudusScope, an accessible, interactive do-it-yourself smartphone microscopy platform that promotes exploratory stimulation and observation of microscopic organisms, in a design that combines the educational modalities of build, play, and inquire. The LudusScope’s touchscreen and joystick allow the selection and stimulation of phototactic microorganisms such as *Euglena gracilis* with light. Organismal behavior is tracked and displayed in real time, enabling open and structured game play as well as scientific inquiry via quantitative experimentation. Furthermore, we used the Scratch programming language to incorporate biophysical modeling. This platform is designed as an accessible, low-cost educational kit for easy construction and expansion. User testing with both teachers and students demonstrates the educational potential of the LudusScope, and we anticipate additional synergy with the maker movement. Transforming observational microscopy into an interactive experience will make microbiology more tangible to society, and effectively support the interdisciplinary learning required by the Next Generation Science Standards.

## Significance Statement

For centuries, observational microscopy has greatly facilitated biology education, but we still cannot playfully interact with the microscopic world we see. Other media such as robotics construction kits, programming languages for children, and video games have demonstrated the tremendous educational potential of interactivity. We developed the LudusScope, a smartphone microscope platform that empowers users to interact with microorganisms by optically stimulating them, allowing structured and open-ended play, true scientific inquiry through experimentation and modeling, as well as learning through construction and modification of the device itself. Transforming observational microscopy into an interactive experience will make microbiology more tangible to society, and effectively support the interdisciplinary learning required by the Next Generation Science Standards.

## Introduction

For centuries, observation through microscopes has constituted a cornerstone of life-science education and research, but opportunities for playful interaction with microscopic living matter remain essentially non-existent. This situation is in striking contrast to other fields. For example, interactive robotic construction kits facilitate exploration of mechatronics [1–2]. Video games and programming nurture interest in engineering and science across ages and genders [3–6]. Unstructured play as well as structured games sustain engagement and motivate learning in various educational contexts [7–10], while accessible languages like Scratch motivate children to self-learn programming [11]. These playful and interactive approaches have translated advanced scientific concepts from industry and research labs into classrooms and other informal learning contexts [12–14]. But the life sciences are lagging in this regard, and we are only seeing the beginning of direct interactivity between learners and the microbiological world [15–19].

Furthermore, multiple organizations including the National Research Council of the National Academies [20], the American Association for the Advancement of Science [21] and the National Science Teachers Association have jointly developed the Next Generation Science Standards (NGSS) [22]. The NGSS are now introduced to US K-12 education and particularly emphasizes the need for new and cross-disciplinary educational approaches, active learning approaches and teaching critical thinking [23–24].

**Fig 1:**
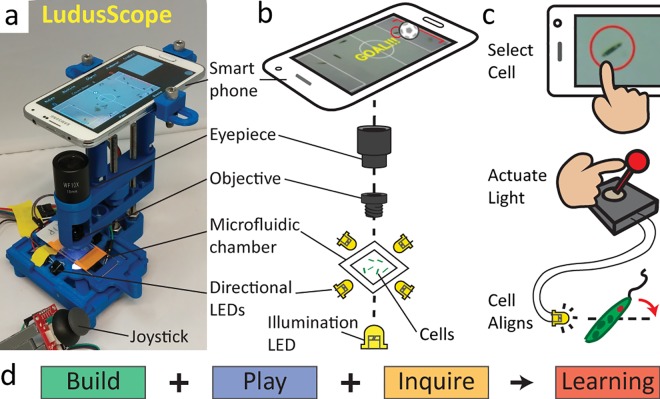
The LudusScope turns observational microscopy into an interactive experience by enabling open-ended interaction with microorganisms. (**A**) The LudusScope with smartphone attached displaying an interactive biology application. (**B**) The optics of the LudusScope mimics a traditional microscope, but four additional LEDs allow stimulation of motile microorganisms, e.g., *Euglena gracilis*. (**C**) The user can interact with individual cells by digitally selecting them via touch screen, and then operate a joystick to actuate the four directional LEDs, which then provide light stimuli to induce phototactic responses, e.g., the cell’s swimming direction aligns with the light. (**D**) The LudusScope integrates three essential educational modalities: Build, play, and inquire.

We therefore set out to develop an accessible cross-disciplinary biology platform that utilizes smartphone microscopy to enable open-ended interaction and exploration with microorganisms (Fig 1). This platform consists of a 3D-printed microscope with an enclosed microfluidic chamber (Fig 1*A* and 1*B*) containing *Euglena gracilis*, which are observed on a smartphone. The user interacts with this single celled organism in two ways: by selecting them on the screen for real-time tracking and via light stimuli that are actuated with the joystick (Fig 1*C*). We focused on smartphone technologies given their wide distribution, entertainment value, increased use in education [25], and potential synergy with emerging hand-held diagnostics for environment and health applications that rely on similar technology [26–29]. We call this open-source platform the LudusScope, as the Latin term “ludus” refers to “play/game” as well as “(elementary) school”; embracing a historical perspective on how playfulness and learning are intertwined [30–31]. We designed the LudusScope for easy replication and we developed smartphone applications for both serious science and playful games, thereby incorporating building, playing, and inquiring as three major modalities for science, technology, engineering, and math (STEM) learning (Fig 1*D*).

## Results

### The Interactive LudusScope Builder Kit

**Fig 2:**
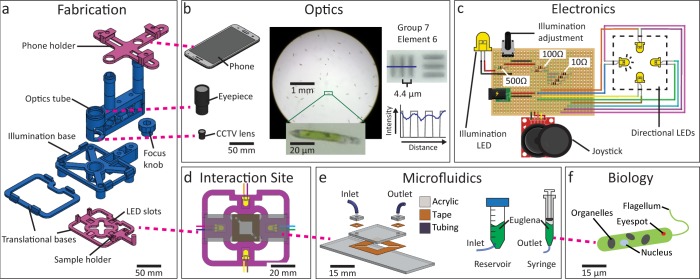
BUILD: All components of the LudusScope are designed to allow straightforward do-it-yourself (DIY) reproduction and emphasize learning in construction, optics, electronics, microfluidics, and microbiology. (**A**) Computer-aided design and assembly of the 3D-printed components of the LudusScope. Alternatively, a variation of the sample holder and phone holder (pink) enable mounting to a conventional microscope (S1 Fig). (**B**) The optical components consist of a smartphone camera, an eyepiece, and a closed-circuit television (CCTV) board lens. Utilizing a conventional eyepiece allows two-way compatibility of the smartphone and software with regular microscopes. The DIY scope is able to resolve Group 7, Element 6 targets, corresponding to a resolution of 4.4 μm. (**C**) Simple electronic circuit for analog light stimulus control of the four LEDs via joystick; a potentiometer controls the illumination LED of the microscope. (**D**) Sample holder with microfluidic slide and four directional LEDs pointing toward the center of the holder. (**E**) The microfluidic chamber is built via sticker microfluidics. A pair of syringe reservoirs connected to both ends of the chamber allows long-term *Euglena* culturing. (**F**) *Euglena* is used given its robust phototactic behavior and easy culturing conditions.

We designed the LudusScope to be accessible, expandable, and educational. Fig 2 summarizes the instructions for building the LudusScope (see Materials and Methods and Supplementary Information (S1 Note and S2 Video). These instructions can be broken down into five educational core concepts [22]: fabrication, optics, electronics, microfluidics, and microbiology [32] (Fig 2).

The 3D printed frame and optics emulates conventional microscopes to optimize integration into existing educational curricula. A nested set of sliding platforms supports standard microscopy slides and enables translation of the field of view (Fig 2*A*). A large focusing knob allows accurate positioning of the optical components for focusing. The optical path consists of a closed circuit television (CCTV) board lens and a standard 10x eyepiece (Figs 1*B* and 2*B*). The CCTV lens sits on a threaded seat, allowing fine focusing. The final image is projected onto a smartphone camera but the microscope is also functional without the phone. Additionally, most smartphones fit the adjustable holder. The imaging quality is sufficient to reveal subcellular details at a resolution of 4.4 μm (Fig 2*B*). These components provide the opportunity for students to learn basic fabrication and optics.

The major difference from a standard microscope is that the LudusScope sample holder includes four LEDs that point toward the center of the sample (Fig 2*C* and 2*D*), providing directional light stimuli. The analog joystick controlling these LEDs enables users to explore the cells’ response to varying light intensities. A circuit couples the analog joystick to a voltage divider to control the four directional LEDs. A diffused white LED is used for illumination, with brightness controlled by a potentiometer (Fig 2*C*). Despite its simplicity, this circuit covers a variety of electronics fundamentals.

The microorganisms are contained in easy-to-fabricate “sticker microfluidics” [33] (Fig 2*E*), which uses two acrylic layers assembled with double-sided tape with an inlet and an outlet, also providing an accessible introduction to the rising technology of microfluidics. For the LudusScope we focused on *E*. *gracilis*, a single-celled protozoan that exhibits negative phototaxis at high intensities [34] (Fig 2*F*). This light response is visible within seconds, allowing users to influence the cells’ orientation and swimming direction via light stimuli in real time. While *Euglena* are generally robust to ambient lighting conditions and thrive in typical indoors lighting, extremely bright conditions such as direct sunlight can have an adverse effect on *Euglena* behavior (S1 Disc). *Euglena* are widely used in educational settings due to its attractive appearance, culturing simplicity, robustness, ease of purchase, and safety, and the existence of many *Euglena*-based biology curricula [35].

We developed hardware and software expansions to demonstrate the LudusScope’s wider potential. Complexity and cost can be decreased further by just building the smartphone holder and interactive sample holder (Fig 2*C*) which can be mounted onto an existing school microscope (S1 Fig). This alternative is particularly suitable for classroom use where students already have access to microscopes and if fabrication is not the educational focus. We also implemented circuit and starter code for Bluetooth and Arduino communication (S4 Note), which allows more advanced data collection and real time data display.

### The Biotic Game-play Environment

**Fig 3:**
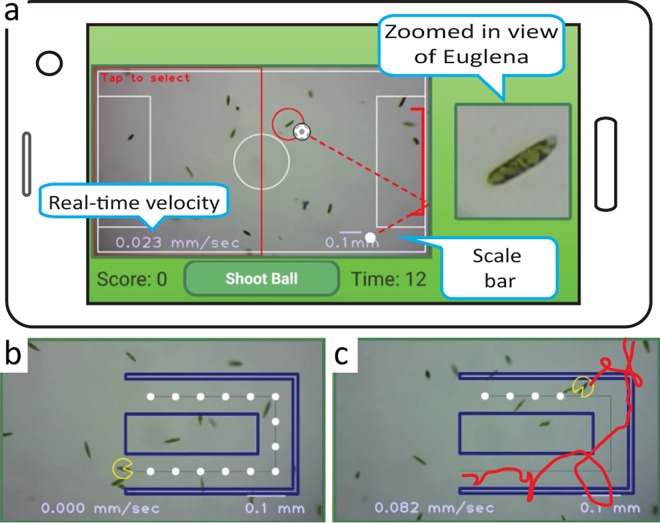
PLAY: The interactive microscope platform enables biotic game play with live cells. (**A**) Soccer-like game sequence in which the virtual ball is picked up, aimed, and carried or passed by *Euglena* toward the goal. The position of the ball is aligned with the swimming direction of *Euglena*. The screen emphasizes features relevant for biology education (cell speed, size, shape, and behavior). (**B**) Other games can be developed, such as “Pac Euglena”. (**C**) The sequence shows a convoluted path as the player tries to get the *Euglena* around the path. At the end of the game, the swimming path recorded during the game is displayed onscreen, connecting game play with educationally relevant content. (S4 Video).

To augment interactivity with biology, we developed biotic games for the LudusScope (Fig 3). Through multiple iterations of design and user tests we developed a simple soccer-themed game in which the player “dribbles” or “shoots” a virtual soccer ball into a virtual goal (Fig 3*A*; S3 Video). A red circle indicates the region of interest (ROI) where image recognition is performed to track the position of a single *Euglena*. The ROI follows the *Euglena*, and swimming direction is calculated from changes in the position of the ROI. The swimming direction is in turn indicated by the position of the soccer ball, and a dotted line projecting the path. Tapping a button “shoots” the ball along the dotted line. To facilitate learning while playing, the application features scale bars, zoomed-in views, and real-time speed readouts. Image processing and object recognition (red circle, Fig 3*A*) rely on the open-source computer vision library OpenCV.

Additionally, we developed a PacMan-inspired game (Fig 3*B* and 3*C*; S4 Video) in which a player taps the screen to select a cell (Fig 3*B*). A virtual “maze” appears on the screen, and the player must guide the cell (via light stimulation) to collect a series of virtual objects. The swimming path is recorded and displayed at the end of the game to allow the player to compare their path to the designated path from the PacMan-inspired game (S4 Video). These traces give insight into the biological behavior of *Euglena*. These applications allow users to interact with biology while being presented with physical information such as speed, size and positions of the organisms. This creates a unique opportunity for learning in which quantitative measurements allow cross-disciplinary teaching of basic physics alongside biology, while presenting the topics in an inviting medium.

### Scientific Inquiry with the LudusScope

**Fig 4:**
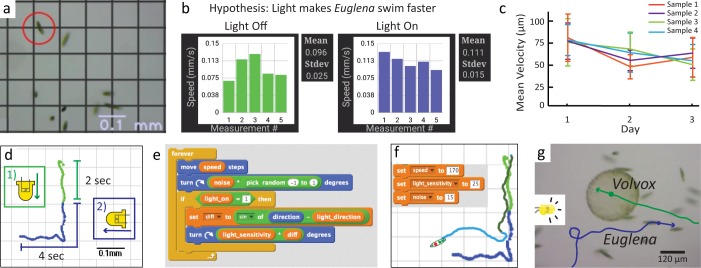
INQUIRE: The LudusScope enables scientific inquiry via quantitative hypothesis testing, measurement, and modeling. (**A**) Free-play exploration interface emphasizes learning with grid overlay. (**B**) Screenshot of app in which the user quantitatively tests hypotheses of whether *Euglena* cells swim faster under different conditions (S8 Video). Elements of the screenshot have been rearranged for illustrative purposes. In this case, the experiments do not support this hypothesis, as the differences between the mean are well within the standard deviation and standard error of mean (not shown). (**C**) With the same app, speed can be tracked over multiple days. In this example, four isolated populations were tracked for three days. The error bars are standard deviation, and show the large variability in *Euglena* speeds. (**D**) Another application allows the user to select individual *Euglena* and display their swimming traces; the blue and green portions represent the time before and after a change in light stimuli (S5 Video). (**E**) Models (simulations) of *Euglena* behavior in response to light are programmed in Scratch. The code portion displayed contains all relevant governing equations; the model parameters (orange) are speed, turning sensitivity to light, and noisiness of swimming path. (**F**) Euglena game emulations can be programmed and model parameters can be tuned to match the behavior of real *Euglena* (S6 Video). (**G**) Co-culturing *Volvox* (green) and *Euglena* (blue) demonstrates opposite phototactic response to light (S9 Video).

To demonstrate formal scientific inquiry with the LudusScope, we implemented a set of applications that enable observation, collection of quantitative data, and hypotheses testing (Fig 4). First we designed a free exploration interface that lacked game elements. In addition to features such as scale bars and speed readings, interface includes a grid overlay, which can be switched on or off in order to promote measurements (Fig 4*A*). In this application, users can tap on the screen to select individual *Euglena* to follow. This interface allows users to freely interact and make observations, without focusing on a pre-defined goal as in the games.

Additionally, we created an application so that users can measure and compare swimming velocities under different conditions (e.g., light on vs. light off) (Fig 4*B*). Measurements are presented graphically and basic statistical analyses are available (S8 Video). This allows testing of hypotheses such as that changing light conditions change swimming speed of *Euglena*. In this case, the results shown in Fig *4B* highlight the importance of considering standard deviations, which show the speeds with and without light do not differ from one another more than the noise. While rigorously testing hypotheses with P-Values and T-tests is outside the scope of most high-school courses, this basic analysis serves as an introduction to students on the importance of considering more than just the mean when drawing conclusions. Furthermore, the application can be used to collect data over multiple days allowing students to investigate long-term culture behavior (Fig 4*C*).

Another application allows users to collect swimming traces of *Euglena*. Users select a Euglena by tapping on the screen, which also starts the tracking (Fig 4*D*; S5 Video). The cell’s swimming path is automatically tracked over several seconds. After a few seconds the application alert the user to change the direction of light. A trace is displayed at the end in different colors corresponding to the different light conditions. Meandering paths can reveal the meandering motion of *Euglena*, delays reflect the response times of the *Euglena* to the light stimuli, and the overall path gives insight into how responsive that particular *Euglena* was to light stimulus.

We wanted to incorporate opportunities to learn programming through the LudusScope, and also to enable students to develop biophysical models. The Android environment is rather complex so we turned to Scratch, a programming language for children [11]. We developed a simple computational Scratch model of *Euglena* movement and response to light (Fig 4*E*). Adjusting speed, noise in orientation, and sensitivity to light enables “visual fitting” of experimentally observed behaviors, such as those obtained through the swimming trace application (Fig 4*F*, S6 Video). Hence, incorporation of Scratch empowers users to construct and assess biophysical models, which are important components of modern scientific inquiry [36]. Furthermore, students can also use this *Euglena* model as a basis for prototyping games in Scratch (S7 Video). The LudusScope can also be extended to other microorganisms or mixtures of species. For example, *Volvox* displays positive phototaxis; when mixed with *Euglena*, the directions of movement of each species is clearly specified (Fig 4*G*; S9 Video).

### User Interaction Studies: Utility for Education

**Fig 5:**
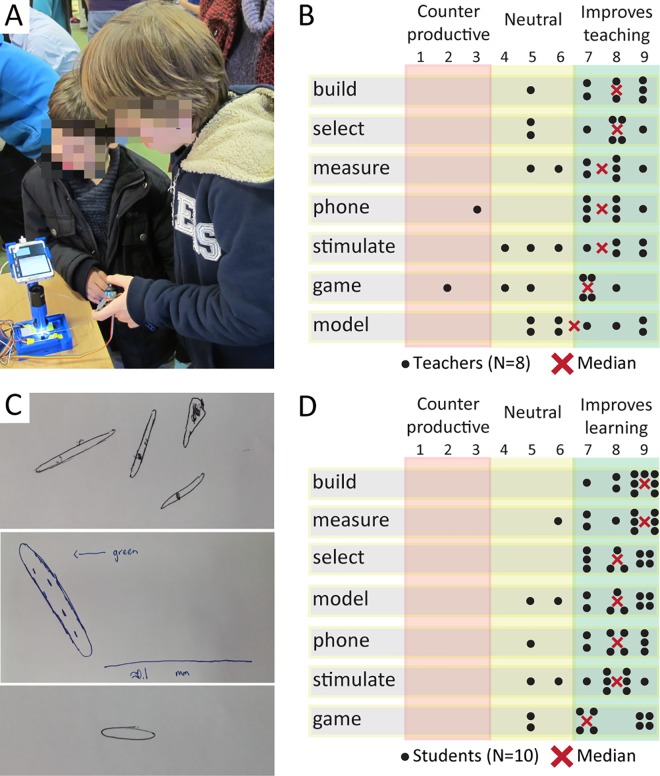
User studies demonstrate the potential of the LudusScope for STEM education. (**A**) An example of a user study setting in which children interact with the LudusScope. (**B**) Teacher scoring (n = 8; Likert scale, 1–9) of seven potential advantages of the LudusScope over currently existing teaching practices. Overall, these scores highlight the utility of the LudusScope; the self-building and measurement features seemed most desirable to these teachers. “Build” corresponds to the construction aspect of the LudusScope, “select” is ability to choose *Euglena* to follow, “measure” is ability to make quantitative measurements, “phone” is the integration of a smartphone, “stimulate” is the directional light stimulus and phototaxis of *Euglena*, “game” is the utility of games for learning, and “model” is the utility of the Scratch model for learning. (**C**) Examples of student drawings of *Euglena* based on observations with the LudusScope. (**D**) Student scoring (n = 10; Likert scale, 1–9) of seven potential advantages of the LudusScope over currently existing teaching practices. The categories are the same as in the teacher response. Overall, the students rate the platform highly, and align well with the teacher responses.

We performed user tests with the LudusScope with both teachers and high school students. These user studies were designed to test the practicality of the LudusScope and its modules as well as its educational potential. We also gave public demonstrations of our LudusScope modules informally at a number of public occasions, ultimately demonstrating the LudusScope to over 150 users from diverse demographics, including teachers, students, children, and game design professionals (Fig 5*A*). These public demonstrations were well received, and demonstrated the robustness and wide appeal of the LudusScope. Furthermore, we successfully tested that 12 year olds can assemble the setup and operate the various applications using the instructions provided in the supplements; although adult supervision and help as typical for afterschool programs or maker spaces should be provided.

We demonstrated the setup and activities (Figs 2–4) to eight science teachers (individually or in pairs) and solicited feedback on how they value the educational potential of the LudusScope, and which features might be of particular interest (S4 Disc). Afterwards, we also asked the teachers to rate seven features of the LudusScope in terms of their possible utility for their own teaching in comparison to existing instructional approaches (Fig 5*B*). All teachers responded very positively to many of the features that enable direct interaction with the organisms, stating that it would render the biology more tangible for the classroom and facilitate activities such as observation, exploration, data collection, and hypothesis testing. Specifically, teachers liked being able to directly stimulate cells with light and observe cellular responses within seconds (given the “short attention span of students”). Teachers also liked the robustness of the closed observation chamber (“many students just ram the microscope objective into the liquid”). On the other hand, a majority of teachers were skeptical about the immediate utility of Scratch to model *Euglena*, as modeling is currently not commonly used in K-12 education. In discussing lesson plans with the LudusScope, teachers saw potential for a wide range of target audiences of the LudusScope, from 6th grade biology classes to advanced high-school biology classes. Most teachers recommended three rounds of activity with the LudusScope as follows: a general tutorial, serious data collection and hypothesis testing (Fig 4), and gaming (Fig 3) as a form of relaxing reinforcement. Two teachers felt they would rather start with gaming to engage the students, followed by the other activities. Most teachers also reported that they had previously successfully used games in the classroom, and viewed the playful aspects of the LudusScope as important complements of formal science education. Multiple teachers indicated that “kids will love” the soccer game, although one teacher expressed concern that the game “responses are too slow” compared to existing “fast-paced video games.” Overall, the LudusScope was perceived as fitting the general push toward more integrated and cross-disciplinary teaching in schools, and multiple teachers expressed interest in testing it with their students in the future.

Similarly, in order to assess the potential for student engagement and learning we carried out a series of one-on-one student sessions (16–18 years old) (n = 10 student participants). In these one-hour activities, approximately half of the time was dedicated to an introduction to the LudusScope, the Euglena Soccer game, and a student worksheet, which was designed to emulate an assignment fitting within a typical class session. The remaining time was dedicated to an introduction to the Scratch simulation and a series of pre- and post-questionnaires. We recorded feedback throughout the study (see S4 Disc for more details). Overall, the students were able to easily operate the LudusScope, and demonstrated engagement throughout the activity. All students (10/10) were able to successfully operate the microscope. While playing the Euglena Soccer game, all students (10/10) successfully finished the worksheet (S3 Note) and were able to accurately make qualitative (color, light response) and quantitative (size, speed) observations about *Euglena*. When drawing the cells (Fig 5C), all students (10/10) correctly captured the elongated cell shape, many (9/10) drew intercellular details, one included a scale bar, and one drew several cells capturing the morphological diversity of the *Euglena* population. Throughout the activity and gameplay, many of the students (9/10) displayed clear signs of enjoyment such as laughter, excitement, and comments such as “[I] gotta beat the high scores!” Like the teachers, the students suggested a broad range of age groups (3^rd^-12^th^) that the LudusScope could be applicable for, the most common suggestions being 8^th^ to 9^th^ grade.

After the activity, the students filled out a questionnaire in which they rated the educational utility of the same seven aspects as the teachers (Fig 5D). The students mirrored the teacher response in that the ability to build, measure and select *Euglena* were favored. Most students (9/10) indicated interest in building a LudusScope. The students rated the stimulation of organisms and ability to play games lowest in the questionnaire. However, when students were verbally asked to describe their favorite aspects of the LudusScope, gameplay (5/10) was the most common response, followed by the ability to observe *Euglena* (4/10), to build the scope (3/10), and to interact with the *Euglena* (3/10). Interestingly, the two students that rated gameplay the lowest in the questionnaire (Fig 5D) also mentioned gameplay as a favorite aspect of the LudusScope. This suggests that while some students do not consider the gameplay particularly educational but nevertheless enjoy it–pointing to the potential of games to support other educational modalities as a motivational component. Regarding future improvements, student pointed to the occasional need of re-focusing the microscope while playing (3/10), the estimated cost of building a LudusScope (3/10) and the ergonomics of the gameplay controls (3/10). Further iterations of the LudusScope design can potentially address these issues. While the simulation was well rated in the questionnaire (Fig 5D), when asked to compare and contrast the Scratch simulation to the LudusScope many students (6/10) stated a benefit to interacting with the real biology, e.g., one student stated, ‘‘it is more convincing if you have a real cell than someone telling you ‘this is what happens.’ Usually the programmed behavior is not as convincing.” When asked what they had learned in the activities, the students self-reported a wide variety of topics, e.g., something related to biology (10/10), about microscopy (5/10), and about Scratch programming (1/10).

## Discussion

We developed the LudusScope (Fig 1), an accessible interactive biology platform that combines educational objectives via building, play, and inquiry. The LudusScope exploits free play (Fig 3) and structured investigation (Fig 4) to enhance curiosity and to empower learners to make observations on the structure and dynamic behavior of microorganisms, thereby providing a starting point for deeper investigation that ultimately can transition into the scientific method. Building a LudusScope (or parts of it) integrates knowledge and skills from fabrication, electronics, biology, optics, and microfluidics. Being able to modify and expand the LudusScope empowers additional interdisciplinary learning, following Papert’s vision of “low-floor/high-ceiling/wide-walls” [1], [37]. All these characteristics make the LudusScope particularly well suited for promoting cross-disciplinary learning. Based on user studies (Fig 5; S2 Disc and S4 Disc), we conclude the LudusScope has versatile potential in formal K-12 education. While we acknowledge that the high school student group is not representative of the general population, we believe the study sufficiently demonstrates the feasibility of the technology. The many layers of activity possible through the LudusScope make it applicable to both middle school and high school which aligns well with many biology and science curricula [22], and even college, where we previously established a similarly themed, but more advanced device-engineering lab course [19].

We envision multiple avenues for wider adoption: Since microscopes are already present in many schools and smartphones and tablets are becoming increasingly available, the microscope attachment (S1 Fig) seems the most attractive route of dissemination in classroom settings. Self-builder kits for schools, makerspaces, and do-it-yourself (DIY) individuals also seem attractive. The self-builder price for the full setup is ~$100, including the cost to have a 3^rd^ party 3D print the structural components. With access to a 3D printer, the cost drops to ~$60. All components are either 3D printed, or commercially available. For the microscope attachment the cost drops even further, and with mass production (in particular avoiding the cost of 3D printing) the cost is estimated to drop to ~$30. Overall the LudusScope is much more accessible than previous *Paramecium*-based biotic games [18], in particular regarding simplicity of hardware design, and long term stability of the organisms. All parts lists, code, and building instructions are open source for ease of adaption and modification (S1, S2 & S4 Notes), and we invite stakeholders from all backgrounds to participate. During development, we identified multiple design principles for interactive biology applications and biotic games in general [38]. We suggest adopting the perspective that the player interacts with cells rather than controls or manipulates them—all the player controls is a physical stimulus to which the cell has significant freedom to respond. This may help to develop a positive disposition towards microbial life [39]. We also speculate that using mobile devices with integrated microfluidics synchronously for diagnostic, educational, and entertainment purposes could accelerate their uptake given shared underlying technology [32], [18], [26], [27].

In summary, the LudusScope relies on a lush intersection of scientific disciplines such as fabrication, biology, optics, computer science, and electronics. The user studies in this paper provide a basic validation of the LudusScope from the perspectives of both educators and students. The LudusScope is now ready for use and in-depth evaluation in formal and informal learning environments at various ages. In addition, lowering the barrier of access for microscopy stands to benefit low cost diagnostic approaches [40]. Transforming observational microscopy into an interactive experience that blends structured inquiry with unstructured play will make the bio-sciences and engineering more accessible and fun for society, and particularly support the interdisciplinary instruction emphasized by the Next Generation Science Standards.

## Materials and Methods

### Biology

*Euglena gracilis (Euglena)* was obtained from Carolina Biological (#152800). The culture were kept in the original glass containers with the lids loosened or in an open reservoir at room temperature (~24°C) and ambient light (~1,000 lux). We maintained the cells in a simple reservoir (Fig 2*F*) for up to a month (S1 Disc), providing a steady supply of fresh organisms for the games and experiments. Longer-term culturing can be achieved using simple growth medium in accordance with the distributor’s instructions. We obtained *Volvox* from Carolina Biological (#131864). *Volvox* (S9 Video) was observed in a droplet on a glass slide as it is too big to fit in the current microfluidic chip.

For the speed measurement example, the *Euglena* were measured over three days. Reservoirs were prepared by filling 15 mL conical tubes with 5 mL of a fresh *Euglena* culture. The conical tubes had a hole drilled in the side into which plastic tubing (Tygon, AAQ04103) was inserted and glued. The plastic tubing connected to a sticker microfluidic chamber. The outlet of the chamber was connected via plastic tubing to a 1-mL syringe. Three vials were kept under ambient light (~1,000 lux). All vials were kept with the lids loose to promote oxygen exchange. To assay organism responsiveness, at a random hour every day for three days, swimming speed was measured using the speed-measurement app, and results were averaged for each sample of each day (n = 10 per culture) (Fig 4*C*).

### Construction Kit

All structural components were designed in SolidWorks (Dassault Systemes, v2014) and 3D printed with ABSplus plastic (Stratasys uPrint+). Microfluidic chips were made with a laser cutter (Epilog Helix, 45 W) on double-sided tape (3M, 300LSE), a 1.5 mm thick poly(methyl methacrylate) base and cover, and tubing (Tygon, AAQ04103), resulting in chamber dimensions of 6.5 mm x 6.5 mm x 170 μm (Fig 2*F*). All of the electrical components were ordered from Sparkfun (S2 Note). The electronic extension used an Arduino UNO board (DEV-11021), transistors (COM-00521), and BlueSMiRF Silver wireless serial cable (WRL-12577) ordered from Sparkfun. We used a 10x microscope eyepiece lens (Cnscope, WF10x, 18-mm field of view, 23.2-mm mount), a 35-mm CCTV board lens (M12 Lenses, PT-3621), and a CCTV lens threaded seat (M12 Lenses, PT-LR001P). A low-cost school microscope (HomeScienceTools, MI-5000DHD) was used for the smartphone-attachment version of the LudusScope. We use an Android Galaxy S5 smartphone, which has a 16-MP rear camera and a 1/2.6” sensor.

### Software

All applications were programmed in Android Studio using the OpenCV library. Our *Euglena* recognition algorithm isolates *Euglena* within images by filtering for green pixel values, followed by contour detection to identify objects of size and shape consistent with *Euglena*. Ultimately, the algorithm tracks the position and calculates the orientation and velocity of individual *Euglena* based on changes in position. All code is available on Github (https://github.com/riedel-kruse-lab/). For the Bluetooth extension, the Arduino Uno was programmed in Arduino. Scratch software is directly accessed in a web browser and can be downloaded and shared (https://scratch.mit.edu). Offline data analysis was performed using ImageJ (Version 1.38, National Institutes of Health, USA), and Excel (Microsoft, v2013).

### User Testing

Development of games and apps involved frequent iterations of informal user testing in our laboratory. We also demonstrated the setup to local science teachers, and an email was sent to a large cohort of these teachers to ask whether they were interested in providing feedback on new educational technology. Eight teachers volunteered; they came to our laboratory alone or in pairs on different days, and we demonstrated the features of the platform and engaged in open-ended discussion about teachers’ needs, experience, and feedback about the LudusScope. A few weeks later, we asked these teachers via email whether they would like to share their opinions for this paper and to rank the utility of various features of the LudusScope; all teachers responded (Fig 5*B*). We also demonstrated the platform to local high school students participating in various summer programs at Stanford University. Ten students participated in the one-on-one study. They came to our laboratory, and were explained the LudusScope. Afterwards each student operated the microscope and played the Euglena Soccer game, while filling out a worksheet. After the biological interaction, the students also played with the Scratch simulation. Finally, the students filled out the same question provided to the teachers, ranking the utility of the various features of the LudusScope. Before, after and throughout the activity the students were asked questions and their responses were recorded. Both formal studies followed guidelines provided by Stanford’s Research Compliance Office (IRB 18334), and were performed with informed consent or a corresponding signed waiver. For minors, a signed written consent was also obtain from their legal guardians.

## Supporting Information

S1 Disc Discussion of culture stability of *Euglena*. (DOCX)

S2 Disc Detailed teacher feedback from user studies. (DOCX)

S3 Disc Potential applications of the LudusScope in the context of Next Generation Science Standards (NGSS). (DOCX)

S4 Disc Detailed student feedback from user studies. (DOCX)

S1 Fig Microscope attachment version of the LudusScope. (*A*) The LudusScope can be adapted to fit onto a standard microscope. (*B*) A 3D printable microscope attachment is needed (S2 Note). The same 3D printed sample holder as the full version can be used, as well as the same circuit sans illumination LED. This alternative approach may be more convenient for classrooms that already have access to standard microscopes.
(TIF)

S2 Fig Qualitative assessment of responsiveness of *Euglena* population over 10 days. To assay the responsiveness of the organisms, at a random hour every day for ten days a video was taken of three different reservoirs of *Euglena* to measure their response to light. The light sequence used was Left, Right, Up, Down, Right, Down, Left, Up for 10 seconds each. The data was analyzed on a qualitative scale of 1–5 for each direction, with 5 indicating clear directional movement as well as immediate clear response to light, 4 corresponding to clear directional movement after time elapsed but not a clear immediate response, 3 corresponding to a weak directional movement, 2 corresponding to completely unclear movement, and 1 to nonmotile/spinning *Euglena*. Every ten second light stimuli interval was assessed on this scale, and the results were averaged for each sample of each day. Error bars are standard deviation.
(TIFF)

S1 Note Building instructions for the LudusScope. (DOCX)

S2 Note Parts list for the LudusScope. (DOC)

S3 Note Basic worksheet suggestion. (DOCX)

S4 Note Links to Downloads. (DOCX)

S1 Video LudusScope overview. Overview movie of the LudusScope and its various applications.
(MP4)

S2 Video Construction of LudusScope. Time-lapse of construction (hardware, electronics, chip, loading).
(MP4)

S3 Video Euglena soccer game. In this single-player game the player needs to score points by dribbling or shooting a ball into the goal. The virtual ball is controlled by a Euglena cell, which is tracked. The player tries to orient the Euglena via the joystick controlling the 4 LEDs. The player may wait until a cell swims “through” the ball to pick it up, or tap with the finger on a Euglena onscreen to pick up the ball there. By tapping the “Shoot Ball” button, the ball is shot a long distance in the direction of the current Euglena orientation. Carrying or shooting the ball into the goal scores 5 points. Tapping to select cost 1 point. The scoring goal switches sides every 30 seconds to prevent that all Euglena end up on just one side. These two different game modes to select the ball (and parameters therein) can be used to significantly alter the challenge and duration of the game. Note scale bar, real-time velocity, and enlarged display of tracked Euglena to naturally emphasize relevant biology content during the game play. Euglena are approx. 50–80 micrometer in length. Speed is real-time.
(MP4)

S4 Video Euglena Pac Man game. Example of the Euglena Pac Man game demonstrating versatility of game design beyond soccer: Here a player selects a cell on screen, then the Pac Man overlay and the maze appears at the corresponding spot, and the player can now guide the cell through that maze via joystick and light. At the end of the game the swimming trace is displayed; demonstrating how game play directly relates to interesting biology content. Euglena are approx. 50–80 micrometer in length. Speed is real-time.
(MP4)

S5 Video Euglena tracing experiment. A science application allows collecting traces of individual Euglena. The cell is guided in one direction first, and after an acoustic signal the player should change the direction. This movie shows two successful and one unsuccessful guidance experiment of a single cell. Note however, that some of the other cells still respond as expected.
(MP4)

S6 Video Euglena scratch simulation. The Euglena’s behavior can be easily simulated by using the Scratch programing language. Code blocks account for the Euglena model and real user-data can be fitted by adjusting model parameters like speed, light-sensitivity, and noise. This movie shows the simulated version of experiments equivalent to the traces in Movie 5, i.e., the image of a trace is inserted into Scratch and can the be fitted.
(MP4)

S7 Video Euglena soccer on Scratch. Scratch soccer. With the Scratch programing language other games can be implemented by using the same code and model parameters as in Movie 7.
(MP4)

S8 Video Euglena speed experiment. Measuring Euglena speed under different light conditions. Euglena are approx. 50–80 micrometer in length. Speed is real-time.
(MP4)

S9 Video Euglena and Volvox phototaxis. Other microorganisms such as Volvox can be investigated simultaneously with Euglena. While Euglena shows a negative photactic response (swims away from the light), Volvox shows positive phototaxis and is thus attracted by the light. Note also how the hydrodynamic flow fields generated by Volvox affect the motion of nearby Euglena. Euglena are approx. 50–80 micrometer in length; Volvox about 300 micrometer in diameter. Speed is real-time.
(MP4)
