# Correction: LudusScope: Accessible Interactive Smartphone Microscopy for Life-Science Education

**DOI:** 10.1371/journal.pone.0168053

**Published:** 2016-12-09

**Authors:** Honesty Kim, Lukas Cyrill Gerber, Daniel Chiu, Seung Ah Lee, Nate J. Cira, Sherwin Yuyang Xia, Ingmar H. Riedel-Kruse

S4 Note contains incorrect information. Please view the correct S4 Note below.

## Supporting Information

S4 Note Links to Downloads. (DOCX)

## References

[1] Kim H, Gerber LC, Chiu D, Lee SA, Cira NJ, Xia SY, et al (2016) LudusScope: Accessible Interactive Smartphone Microscopy for Life-Science Education. PLoS ONE 11(10): e0162602 doi:10.1371/journal.pone.0162602 27706189 10.1371/journal.pone.0162602 PMC5051900

