# Supplementary material for: LudusScope: Accessible Interactive Smartphone Microscopy for Life-Science Education
Source: PLoS One. 2016 Oct 5;11(10):e0162602. doi: 10.1371/journal.pone.0162602 (PMC5051900; doi:10.1371/journal.pone.0162602)
Supplement: S1 Disc — (DOCX) [file pone.0162602.s001.docx]

**Supplementary Discussion 1**

**Euglena Stability**

An important component of the LudusScope and any biology-based interactive platform is the inherent variability in living organisms. While *Euglena gracilis* is an exceptionally hardy organism requiring minimal care, there is still variability over time in the cultures that may impact the quality of interactions. For example, a highly light responsive culture may for unknown reasons respond less well at later times – and then performance may increase again. We suspect cell and culture internal state changes (such as circadian rhythms, adaptations to different oxygen levels etc.) as well as environmental influences (such as temperature) to be the reason, which are hard to control for; one thing we clearly noticed is that direct sunlight has a strong and dramatic adverse effect.

Following are several tips to maximize the reliability of Euglena culture:

Based on our experience, a *Euglena* culture from Carolina Scientific will reliably last up to a month with acceptable gameplay when stored in the original container under decent room light. Over this time, the *Euglena* decrease in responsiveness, speed, and viability. Therefore, a fresher *Euglena* culture will perform better. As an alternative, Carolina Scientific offers media preparations that can be used to exchange old media, which can extend the life of a culture well beyond a month.

When keeping Euglena inside the “interaction chamber” (Fig.2D left), response quality and speed usually goes down within 2-5 days (Fig.4C). To compensate for such a less responsive culture, the reservoir/syringe system (Fig.2E right) can be used to exchange organisms between reservoir and chamber to find responsive subpopulations of cells (Fig.2E), i.e., by pulling on the syringe one can flush *Euglena* through the chip until one finds a stable population.

Alternatively, one can always load fresh Euglena from Carolina into the reservoir/syringe system. These fresh cultures can be additionally enriched for highly light responsive Euglena: To do this, the *Euglena* culture should be transferred to a container to about 1 cm deep. A strong light should then be applied from the bottom of the culture. After a few minutes, dense green regions of *Euglena* bioconvection patterns should be visible on the surface. The top segment should then be collected and transferred to another container for using a pipette. This technique will not only collect highly responsive *Euglena*, but will also remove immotile *Euglena*.

The *Euglena* culture is generally robust, and can be maintained in storage and in the dark for a couple days without significantly impacting the health of the culture. This makes the organisms easy to transport. We have on multiple occasions left the organisms in dark backpack and luggage cases for long periods of travel, and found they are responsive afterwards. However, one should clearly watch out for direct sunlight, which can adversely affect the responsiveness of *Euglena* cultures.
