# Supplementary material for: LudusScope: Accessible Interactive Smartphone Microscopy for Life-Science Education
Source: PLoS One. 2016 Oct 5;11(10):e0162602. doi: 10.1371/journal.pone.0162602 (PMC5051900; doi:10.1371/journal.pone.0162602)
Supplement: S2 Disc — (DOCX) [file pone.0162602.s002.docx]

**Supplementary Discussion 2**

**Detailed Teacher Feedback**

In order to assess the potentially utility for classroom use we demonstrated the setup to eight science teachers. These teachers were doing a summer internship at Stanford University, and were recruited for the study by sending an email to about 30 teachers asking for volunteers who would be interested to come to our lab, get a demo and provide feedback on the setup we had developed. Among the eight teachers who volunteered, five were female, three male; half of them taught biology, the other physics and math, two had taught both math and biology throughout their career; three taught middle school; four high school; one college. Teachers came to our lab – either individually or in pairs. We then demonstrated the components of the setup and engaged in an open conversation about what they liked, what suggestions they had etc. This more open approach and the fact teachers not always being interviewed individually does not allow us to draw quantitative conclusions (e.g., 6 out of 8 had opinion X), but we capture the general trend and opinion by taking notes throughout on their feedback, which is summarized in the following paragraphs. (The quotes provided below may not always be the very exact teachers’ words as we were note taking in real time during the discussion, but the essence and intention of each statement is certainly captured.) After we had interviewed all teachers we asked for some additional feedback – allowing us to achieve a more quantitative picture on their opinions. Note also that some of the teacher feedback has already informed the design of the setup and software as presented in the paper.

Showing the initial setup, teachers complemented the phone attachment as it allows students to work in pairs, pointing out in particular that multiple students can see simultaneously what is happening to the specimen, which would enable direct discussion between students. This is in contrast to traditional eyepiece use, where one student is looking while the others cannot see what is going on. Teachers also liked the direct data analysis (such as estimating density and speed) and data logging on the screen. One teacher stated that children would love to use their phone during class as they are typically forbidden in school. Two teachers also complemented the closed observation chamber (“many students just ram the objective into the liquid”). There were varying opinions on whether the microscope image should be displayed inverted (as seen through the eye piece – and which is often a challenging but important concept for children to grasp) or whether to digitally correct for that (as we had implemented).

Multiple teachers liked the interactive stimulus, i.e., being able to stimulate the cells with light, and it was seen as a plus that these cellular light responses were visible within seconds, given the short attention span of students. Teachers liked the fact that by tapping individual cells could be selected and tracked (teachers comments: “It is like a mini game”, “this is fun”, “would be nice to select two – each student one – and then let them race”). The display of scale-bar and speed was well liked, but multiple teachers wished for a grid overlay to have students measure and calculate size, density, and speed. (We therefore implemented a grid; see Fig.4A.) Being able to read scale and speed directly from the screen was seen as a very useful feature (“children often are challenged with understanding scale”). The enlarged view of the tracked cell (Fig.3A) was also appreciated (“reveals that they are 3D”, “they role around their own axis”); even more augmented detail was suggested including highlighting more subcellular compartments and automatically labeling it.

Taking 5 measurements under 2 different conditions to test a hypothesis was thought to be valuable (Fig.4B), and in particular that everything was graphed automatically. Spending about 3sec per trace was seen as a good rate. (Teachers commented that exporting data to Excel is an important skill to learn – some would like to do that with their students, others felt that it takes too much time and hence the it is good that students can directly interpret the data from the phone screen.) Teachers stated that this activity would teach students how to read graphs, compare conditions, and make them recognize variability. 5 data points might be the right amount for middle schools, high schools should collect more, and utilize data for AP statistics classes. The tracing activity under light response (Fig.4D) was seen as useful (even just observing a trace without the light) and good for class discussion. Seeing the meandering motion of *Euglena,* one teacher even commented that one could use this to discuss random walks with students.

Most teachers stated that they have good experiences with games as learning tools given that they are fun and motivating (multiple teacher mentioning good experience with the Colorado Phet simulations, https://phet.colorado.edu/). Regarding our biotic soccer game (Fig.3A) multiple teachers stated that “kids will love it”, while one teacher expressed concern of whether the game responses are too slow (turning light for 5 sec into one direction – compared to the much faster paced video games that children are used these days might run the danger of losing students interest). Some teachers stated it would be better to have a two player game, but there was varying opinion on whether competitive games are a good or bad thing. Some expressed that competition additionally motivates the children, while another teacher stated that it often leads to “trash talk” and related behavior, and this particular teacher preferred to have students play after each or play collaboratively. Some teachers said that games have a “goal oriented” aspect, which is good for getting students focused to the task.

Teachers were more skeptical and divided on the direct applicability of Scratch, many teachers had not used this programming language before, and they also stated that programming in general is still in the process of finding its proper place in formal education. Especially in biology education programming is not used much yet, and teachers also emphasized that the Next Generation Science Standards (NGSS) puts more emphasize on cross-disciplinary approaches and that consequently integrating programming much more strongly into biology education is certainly desired, at the same time will require new curricula and training of teachers. Two teachers with robotics teaching experience stated that the modeling and programming would nicely align with those classes. Regarding age multiple teachers pointed out that trigonometry is only taught in high-school hence the modeling (which currently uses a sin-function, Fig.4E) would have to be easier if used for younger students. Opinions among teachers also widely varied on the usefulness of parameter fitting (Fig.4F), ranging from “random parameter fitting with no gain” vs. “parameter fitting by iterated guessing is not uncommon and very useful” and “very instructive to fit something that is not just a line”. Teachers also acknowledged that (mathematical) modeling for biology is not really done much in school, but that it will certainly be more important in the future given the NGSS. And some teachers commented that the model could also be used much more as a black box.

Regarding constructing the whole microscope (Fig.2) or just the attachment (*SI, Figure 1*), the teacher cohort stated that none of them teach any fabrication skills, but that the advancement of maker movement, 3D printers etc. should enable strong integration and make it relevant to the students. One physics teacher stated that electric circuits on breadboards would make a good to connection to electricity in physics teaching; one teacher commented that many kids actually like soldering. There was overall acknowledgement for the potential for cross subject teaching and integration.

All teachers agreed that the setup and its application are relevant for formal education and that the primary target audiences based on specific presentation were identified for ~6^th^ grade biology, as well as for more advanced high-school classes including AP biology – and also cross disciplinary teaching such as analyzing data in a statistics class; one teacher mentioned that some of the activities might already work from 3^rd^ grade on.

Regarding logistics teachers largely agreed that typically class sets of 18 setups (for 36 students) would be needed. Assuming that such as class-set of microscope attachments (*SI, Figure 1*) might cost $500, this would constitute a reasonable expense. Usually, the more pressing questions of whether to purchase such technology are (1) long-term maintenance (e.g., schools typically have money to buy new microscopes and other equipment, but one equipment breaks down over time, schools usually do not have funds for repairs), and (2) to what extend any new technology can be used for many different activities across ages and throughout the year (e.g., would this setup only be use for one lesson in 6^th^ grade to teach about Euglena, vs. studying different organisms, using it in different grades such as AP biology etc.). That the students provide their own smart phones (one per group) should not be an issue, as increasingly mores students have their own phones. Teachers even commented that students using their phone might actually lower the logistics and cost for the school that would normally have to provide the computing resources. Differences of course exist of how many students have smart phones and what type depending on average family income level in the particular school district. This would require corresponding additional support, but in the long run tablet devices in general get cheaper, are more widely available, and more widely used in education for many subject areas.

When asked whether these activities (play and inquiry in Fig.3 and 4, but without the building project in Fig.2) would provide a good class activity and how to best structure such a class, multiple teachers envisioned the following: begin by demonstrating the system to the class from a screen, motivate the students by explaining that there will be a game with living cells, and follow up with three rounds of activities. The first activity is a general tutorial (Fig.4A), followed by serious measurement and hypothesis testing (Fig.4B,D), and finally finishing with a game (Fig.3). The strategy is to do the “serious work” first while the children still are attentive, and afterwards use a game as relaxing reinforcement while also providing a second chance for students who still haven’t grasped the concepts. Two teachers proposed an alternative sequence: let the students play first a game to get them motivated and to make them understand the setup (i.e., use the game as tutorial), and then go into the more “serious” activities.

The teachers provided a number of suggestions of what other features to implement and changes to make in order to make these activities more useful and user friendly (many of these suggestions have already been incorporated as shown in the paper). Some of the more relevant items were to be able to “Freeze the screen for notebooks”, having grid overlays and timer to do more direct measurements regarding speed, size, density, showing in real time on screen where the light is coming from, being able to control all four LEDs more independently, and having some enlarged view (real or photo) with labels on the Euglena parts.

At the end of the demonstration we also asked each teacher what they found particularly compelling (if at all) about the setup. A few of the comments were: “Having a microscope that is interactive is a big plus and advancement”; “It is not virtual but real (virtual is great – and there is so much going into that direction – but danger of losing sight of the real – this kind of brings that back), also that the [cell] behavior is not deterministic.”; “Screen – as its never clear what kids actually see through the scope.”; “Being able to manipulate with light.”; “Being able to collect and manipulate data, also where variables have clear meanings, also that kids can use their own phones”; [The “meaning of the variables” the teacher referred to implied that a velocity of a cell is something a student can easy understand and relate to.] “Making math more relevant about numbers”; “Game is fun and rewarding”; “Usually students are explicitly forbidden to use phones in school – they would love to be allowed to use it.”

In summary, teachers responded very positively to many of the features that enable a direct interaction with these microscopic organisms, making them more tangible for the classroom, and also facilitating for the students many relevant activities such as observation, exploration, data collection, hypothesis testing, and measurement. The usage of a smart phone was seen as an advantage. The setup is well in line with the general trend towards more integrated and cross-disciplinary teaching (besides the general challenge for many teachers to actually implement that in the class room for logistic reasons and as well as the teacher’s training). It would be important and opportunistic to use this technology for many other school activities beyond *Euglena* biology, such as the tracking other objects with the phone like ants. Playful aspects (both the structured game as well more unstructured “catching cells with your finger”) were seen as important enriching components to support the more formal science and learning parts.

After all the teachers had seen the demo, we sent another short email questionnaire to get a more quantitative understanding of the teacher opinions (summarized in Fig.5B) – and particularly asking whether they could see the use of this setup for their own teaching. This feedback essentially validates their earlier feedback, and we note that the building and experimentation aspects seemed to be better received than the games and modeling.
