# Supplementary material for: LudusScope: Accessible Interactive Smartphone Microscopy for Life-Science Education
Source: PLoS One. 2016 Oct 5;11(10):e0162602. doi: 10.1371/journal.pone.0162602 (PMC5051900; doi:10.1371/journal.pone.0162602)
Supplement: S3 Disc — (DOCX) [file pone.0162602.s003.docx]

**Supplementary Discussion 3**

**Alignment of the LudusScope with Next Generation Science Standards**

The Next Generation Science Standards (NGSS) put forth a set of eight practices that they recommend students use in science education. In the following, we describe the practice highlight by the NGSS, and suggest how the LudusScope may fulfill these practices.

**Practice 1: Asking Questions and Defining Problems.** In traditional microscopy via eye-piece, only one user can observe at any given time. Consequently, student-student and student-teacher communication over observations is hindered. Because the LudusScope uses a smartphone to visualize microscope images, it naturally creates a shared experience between multiple users (*Fig. 1A & S1A*). This promotes an environment conducive to discussion and asking questions. Furthermore, the playful nature of the platform potentially increases engagement and interest of students, encouraging participation.

**Practice 2: Developing and Using Models.** Observations of *Euglena* swimming behavior and light responsiveness is heavily emphasized with the LudusScope through both its games and science apps (*Fig. 3 & 4*). Furthermore, the integration of the Scratch programming language allows students to construct a simple model of *Euglena* phototactic response (*Fig. 4E & 4F*).

**Practice 3: Planning and Carrying out Investigations.** The science applications in the LudusScope are designed to allow students to test hypothesis. The speed app in particular allows students to test the effects of different conditions on *Euglena* swimming speed (*Fig. 4B & 4C*). The tracing application on the other hand promotes observation and descriptions of *Euglena* behavior (*Fig. 4D*).

**Practice 4: Analyzing and Interpreting Data.** The science apps both present data for the students to analyze and interpret. In the speed measurement app, students at the end are presented with data such as mean, minimum, maximum, and standard deviation. Furthermore, measured data is also presented graphically (*Fig. 4B*). The tracing app also produces data in the form of a graphical trace, giving students information on the spatial and temporal response (or lack of) of *Euglena* to changing light stimuli (*Fig. 4D*). These features allow students to extract meaning and insight from their experiments.

**Practice 5: Using Mathematics and Computational Thinking.** The inclusion of scale bars and grids allow students to make mathematical calculations based on observations such as estimation of *Euglena* size, and *Euglena* population density (*Fig. 3A& 4A*). These features in particular allow cross disciplinary experience in physics and biology.

**Practice 6: Constructing Explanations and Designing Solutions.** The design and testing of hypothesis using the LudusScope as well as the modeling via Scratch provides the opportunity for students to construct explanations, and further test the validity of their explanations (*Fig. 4*). Furthermore, the open source nature of project allows iteration and alterations to design solutions to the construction of the LudusScope (*Fig. 2*).

**Practice 7: Engaging in Argument from Evidence.** The LudusScope gives students the opportunity to create and test hypotheses (*Fig. 4B*). The results of these experiments give students the opportunity to argue their conclusions using data-based evidence.

**Practice 8: Obtaining, Evaluating, and Communicating Information.** The curricula for LudusScope-based teaching inherently promotes digestion of information by students. In order to understand *Euglena* behavior, students may seek external sources of information promoting their ability to collect and comprehend scientific literature.
