# Supplementary material for: LudusScope: Accessible Interactive Smartphone Microscopy for Life-Science Education
Source: PLoS One. 2016 Oct 5;11(10):e0162602. doi: 10.1371/journal.pone.0162602 (PMC5051900; doi:10.1371/journal.pone.0162602)
Supplement: S4 Disc — (DOCX) [file pone.0162602.s004.docx]

**Supplementary Discussion 4**

**Detailed Student Feedback**

In order to assess student interactions with the LudusScope, we demonstrated the setup to ten high school students who volunteered to participate in the study. The participants came from a wide variety of high schools in the San Francisco Bay Area, but all were part of various summer programs at Stanford. The participant ages ranged from 16 to 18. Half of the participants were male and the other half female. Of the participants, all ten had operated microscopes before. Most had seen living cells under a microscope (7/10), and only one participant had seen Euglena before. Most also owned smartphones (9/10), with one participant mentioning that smartphones were more common than laptops amongst their classmates. The studies were conducted one-on-one, and typically lasted an hour. There were always two investigators present, one walking the participant through the activities and asking questions, and the other taking notes. The session followed a pre-set template (see details below), but because of the open ended nature of the discussions and interactions, not all participants were always asked the exact same questions.

Each study began with an introduction to the LudusScope and its operation. The investigator pointed out each of the components of the LudusScope to the participant and explained its function in the scope. After verifying that the participant comprehended the operation, the investigator then placed a slide containing freely swimming Euglena into the LudusScope and focused onto the sample using the Android camera app. At this point the participant freely explored the sample and familiarized themselves with the operation of the LudusScope. Then the instructor defocused the microscope and removed the sample, and prompted the participant to try and find the Euglena again. All participants were able the find and focus on the sample. Most were able to do this on the first attempt (8/10), and the remaining participants (2/10) were able to do it on the second try. Among the participants that were directly asked, all (8/8) stated that they recognized the similarities between the LudusScope and a conventional microscope.

After familiarizing the participant with the operation of the microscope, the investigator opened the Euglena Soccer application and prompted the participant to describe all the features on the screen. Most participants (8/10) were able to immediately recognize the objective of the game. A little over half (6/10) of the participants were able to recognize the meaning of the scale bar and speed measurements on their own. After clarifying any confusing areas, the investigator then prompted the participant to play with the Euglena soccer app using just the ability to select Euglena and to shoot the ball. At this point, the joystick control had not been introduced and the participants were unaware of the phototactic response of the Euglena. Almost all of the participants (9/10) played multiple rounds of the game. Most (7/10) of the participants were clearly engaged by the game, displaying one or more of the following traits: competitiveness, frequent laughter/smiling, asking to play again multiple times, or repeatedly returning to the game during later portions of the activity. One particularly competitive participant stated that they, “gotta beat the high score.” In addition to the gameplay, the participants were generally engaged by the interactivity (“[The LudusScope] is pretty cool, because you interact with something live.”) as well as the microscope itself (“The whole setup is really, really cool.”).

After gameplay, the participants were instructed to play with the joystick and observe the LEDs. All of the participants (10/10) correctly identified the relationship between the joystick and LEDs. At this point the participants were given the worksheet (*Supplementary Note 3*), and prompted to answer the questions while using the Euglena Soccer game for reference. All of the participants (10/10) correctly identified the color of the organism (Q1). Furthermore, all (10/10) gave reasonable estimates for the Euglena dimension (Q2). All participants (10/10) gave reasonable estimates of Euglena speed (Q3), and all (10/10) were able to recognize that there were a distribution of speeds (Q4). In both the size and speed measurements, some participants did not notice the decimal point at first. The hardest question based on the time spent (between 30 sec and 4 minutes) for the participants was identifying Euglena response (Q5), and the direction Euglena swim in response to light (Q6). All participants (10/10) ultimately correctly recognized Euglena swim away from light. From these observations (and also earlier informal studies) we conclude that it is crucial that participants get sufficient scaffolding to understand the light response of Euglena, which can be achieved by making sure they understand the relationship between the joystick and the direction of light. Similarly, participants often tend to just turn on the joystick and then let go within a second – expecting an obvious Euglena response (i.e., the similar time scale as most people are likely used to from traditional video games); but the Euglena responses often only become noticeable when keeping one LED on for about five seconds, hence it advantageous to encourage participants to apply longer light stimuli in case they do not do it on their own. Finally, for the last question the participants were prompted to draw a Euglena cell with as much detail as they can (Q7). All participants (10/10) correctly captured the elongated shape of the Euglena. Nearly all (9/10) included some intercellular detail. One participant included a scale bar, and another participant drew several Euglena of varying shapes. One participant also drew a Euglena with a flagellum although the flagellum is not visible from the LudusScope. When asked, the participant responded that they “[knew] cells swim with flagellum”.

After finishing the worksheet, the participant was instructed to play the Euglena Soccer game, except this time with the joystick and light. Of the participants that were asked, most (6/8) felt that the joystick and light control helped them during gameplay. There were two participants who responded that it was more difficult to play the game now, but citing the need for multitasking between the joystick and tapping on the screen as the reason for their response. Of the two participants that were not asked whether the light control was helpful, both scored higher in the game with the light control than without.

Next the investigator demonstrated the Scratch simulation to the participant, and the participant played a game on the simulation. Afterwards the investigator asked the participant to compare and contrast the Scratch simulation and the real biology. There were a variety of responses to this open ended question. The majority of participants seemed to prefer interacting with the real biology: Many participants (6/10) explicitly said they preferred the LudusScope, while none explicitly said they preferred the Scratch simulation. Many participants (6/10) stated that the Scratch simulation responded faster to stimuli/was easier to see. In contrast, one participant felt the real biology was easier to control. One participant suggested starting lessons with the Scratch simulation because “It is easier to understand the concepts with the simulation. On the scope you have to focus on a lot of other things.” A few participants (3/10) stated that the Scratch simulation captured the Euglena behavior accurately. This statement was not mutually exclusive to the previous statement that the simulation responded faster. Two participants pointed out differences in Euglena behavior between the simulation and real biology (beside faster responses). One participant stated, “I think it is more convincing if you have a real cell then someone telling you ‘this is what happens’. Usually the programmed behavior is not as convincing. A programmer can only put just so much variability in and has to leave things out”. Another advantage of the simulation that one participant pointed out is that from a classroom perspective “it is cheaper and gives a good preview of what the real organisms will be like.” The same participant also liked that the simulation was always in focus. In contrast, another participant stated they enjoyed re-focusing the microscope in-game.

After concluding the Scratch simulation, the investigator asked the participant to rate the various aspects discussed and demoed during the activity. The participants were prompted to rate the various activities, with the results as shown in Figure 5 in the main paper.

To conclude the activity, the participants were asked a series of open ended questions. When asked what the participant had learned, there were a wide variety of answers. The most common responses were regarding Euglena biology, and microscopy. When asked how the participant envisioned the platform being incorporated in their classes, four stated that it would fit in well in a biology lab, two mentioned that it would be good for students that are interested in building things, and one mentioned it would be good for cross-disciplinary learning. Three participants also mentioned that it would be good for engaging students that are not necessarily interested in biology. Two participants stated that instead of a regular school curriculum, the LudusScope would be better suited for afterschool or summer programs. When asked what age group the LudusScope would be appropriate for, the participants responded with a wide range from 3^rd^ grade to college. The most common grade suggested seemed to be 8^th^/9^th^ grade. When asked if the participant would be interested in building the LudusScope in either a classroom or personal setting, most (9/10) said they would be. One participant qualified their answer by saying “Not everyone would have the interest or patience.”

To conclude the study, the investigator asked the participant to list the things they enjoyed the most, and to give some suggestions for improvement. Among the aspects the students enjoyed the most, the most common response was the games aspect (5/10), followed by the ability to see live Euglena (4/10). The ability to control/interact with the Euglena (3/10), and the fact that the LudusScope can be self-built (3/10) tied for the third most common response. Amongst the aspects the participants would like to see improved, the most common responses were the cost for building a LudusScope (3/10), ergonomics/controls (3/10) and the need to focus while playing the game (3/10). In addition, the participant felt the scope was too fragile (2/10), and that the application was buggy (2/10).
