# Supplementary figures and images for: LudusScope: Accessible Interactive Smartphone Microscopy for Life-Science Education
Source: PLoS One. 2016 Oct 5;11(10):e0162602. doi: 10.1371/journal.pone.0162602 (PMC5051900; doi:10.1371/journal.pone.0162602)

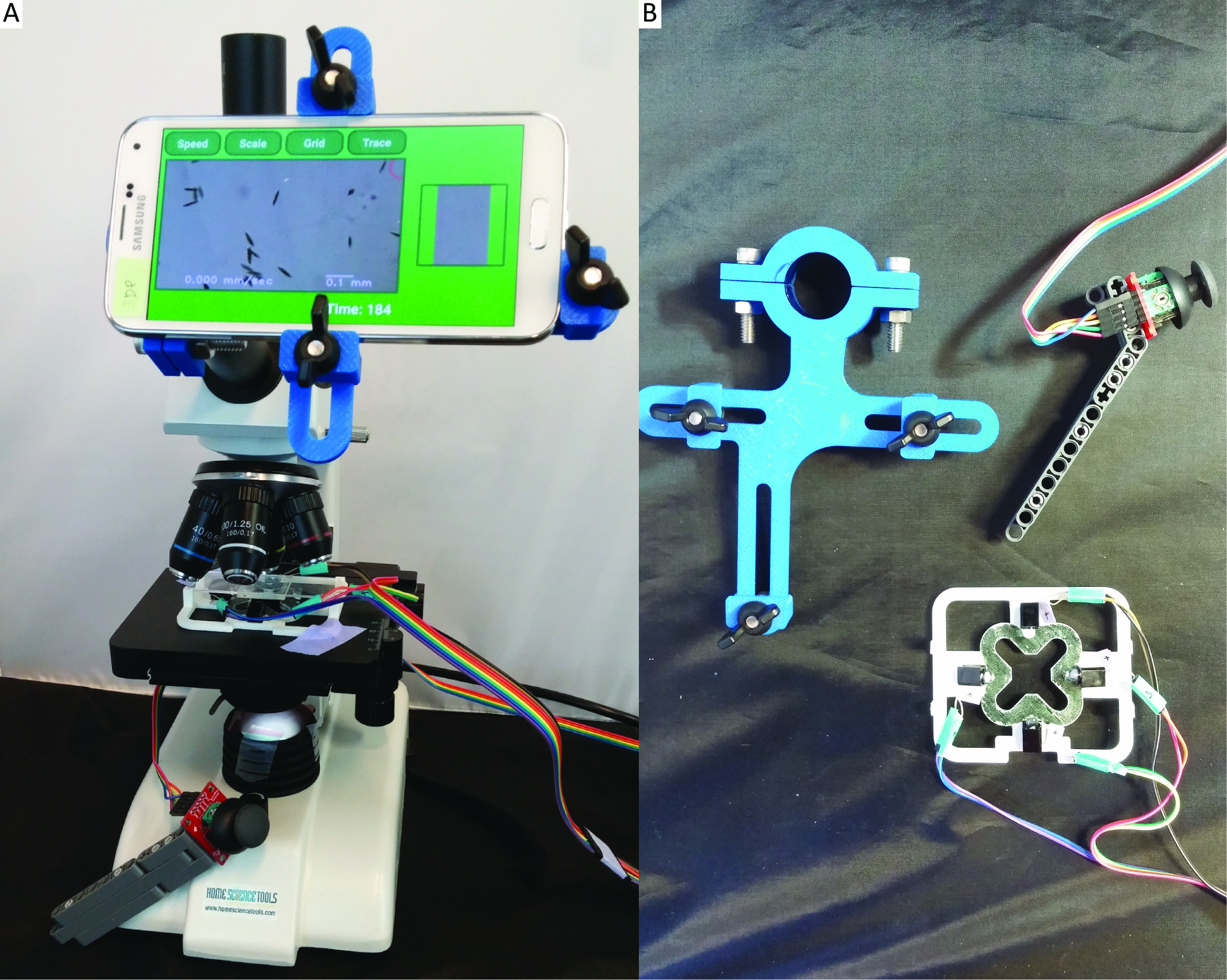

Supplement: S1 Fig — (A) The LudusScope can be adapted to fit onto a standard microscope. (B) A 3D printable microscope attachment is needed (S2 Note). The same 3D printed sample holder as the full version can be used, as well as the same circuit sans illumination LED. This alternative approach may be more convenient for classrooms that already have access to standard microscopes. (TIF) [file pone.0162602.s005.tif]

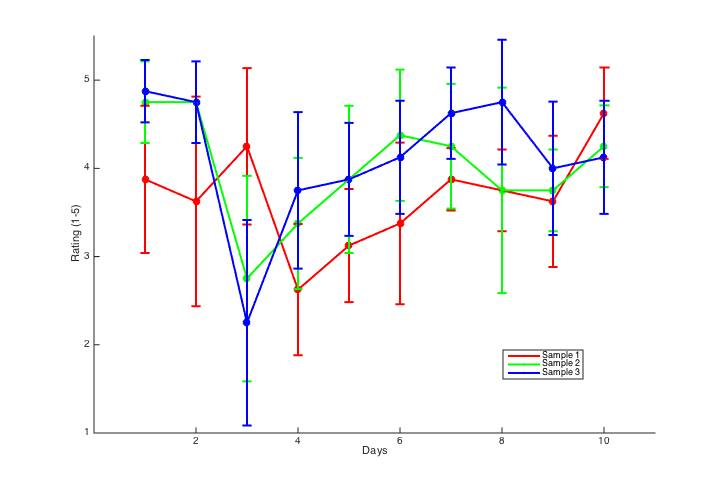

Supplement: S2 Fig — To assay the responsiveness of the organisms, at a random hour every day for ten days a video was taken of three different reservoirs of Euglena to measure their response to light. The light sequence used was Left, Right, Up, Down, Right, Down, Left, Up for 10 seconds each. The data was analyzed on a qualitative scale of 1–5 for each direction, with 5 indicating clear directional movement as well as immediate clear response to light, 4 corresponding to clear directional movement after time elapsed but not a clear immediate response, 3 corresponding to a weak directional movement, 2 corresponding to completely unclear movement, and 1 to nonmotile/spinning Euglena. Every ten second light stimuli interval was assessed on this scale, and the results were averaged for each sample of each day. Error bars are standard deviation. (TIFF) [file pone.0162602.s006.tiff]
