# Supplementary material for: LudusScope: Accessible Interactive Smartphone Microscopy for Life-Science Education
Source: PLoS One. 2016 Oct 5;11(10):e0162602. doi: 10.1371/journal.pone.0162602 (PMC5051900; doi:10.1371/journal.pone.0162602)

**Supplementary Note 1**

**Building Instructions.**

**Overview:**The building of the LudusScope is split in 4 parts. Each section has separate section in this document:

1. Overview & Hardware: This document. Use 3D printed parts. Page 2.
2. Circuit: All electronics and LEDs. Page 27.
3. Mini-aquarium: Microfluidic chip for the Euglena. Page 33.
4. Final assembly and focusing. Page 37.

**Building Instructions 1: Hardware**

**Hardware: Table of contents**

1. Preassembly
2. Assembling the base
3. Sliding platform and illumination
4. Assembling focus knob
5. Assembling optics tube
6. Putting it all together
7. Assembling phone holder
8. Optical components
9. Building finished

**Parts and Tools:**

**
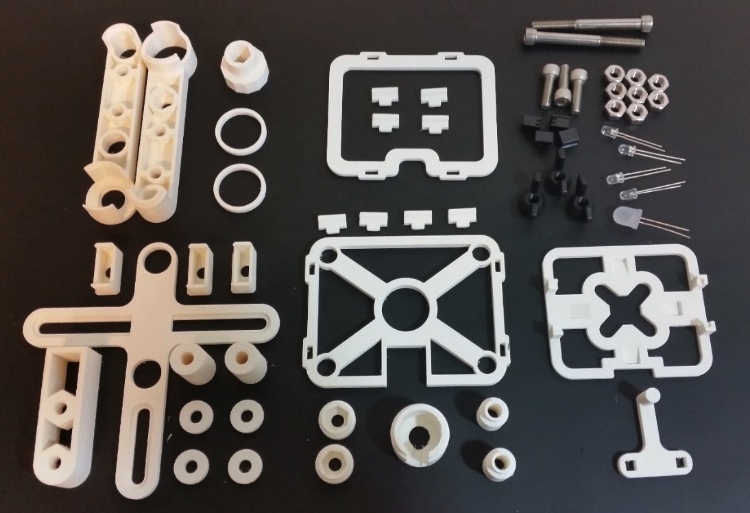
**

- **
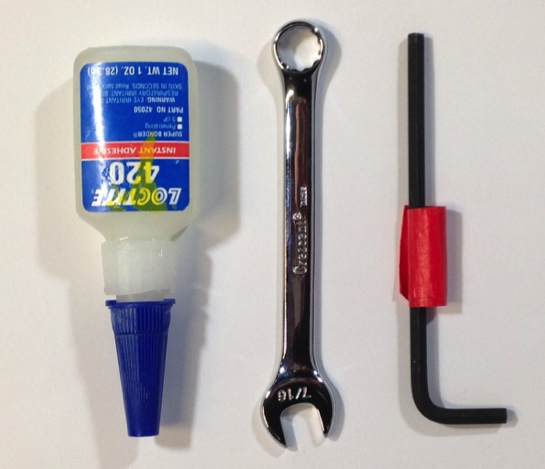
**3/16” Hex
- 7/18” wrench
- Superglue

1. **Preassembly:**

Glue at red arrows.

**
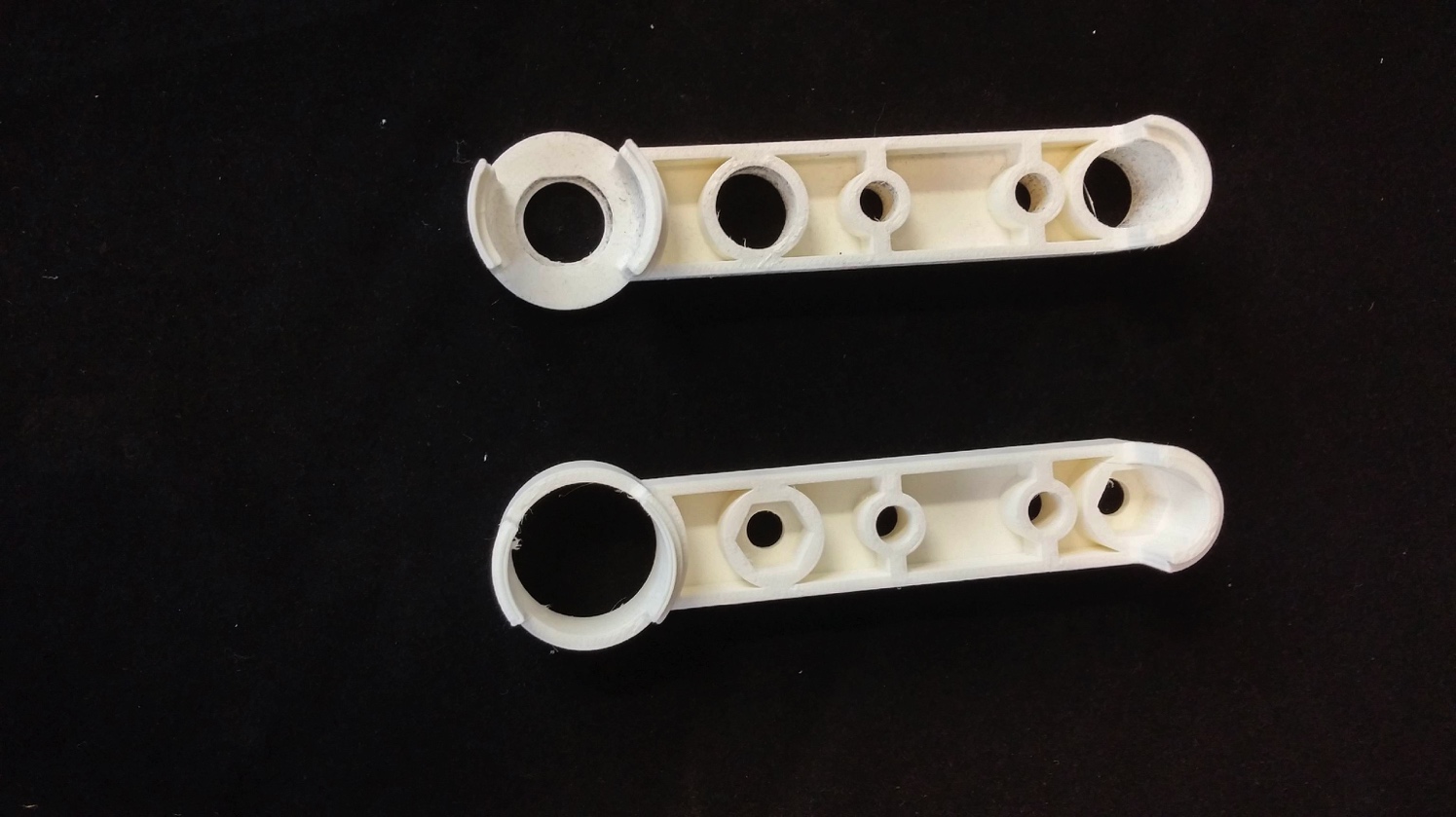
**

i

**
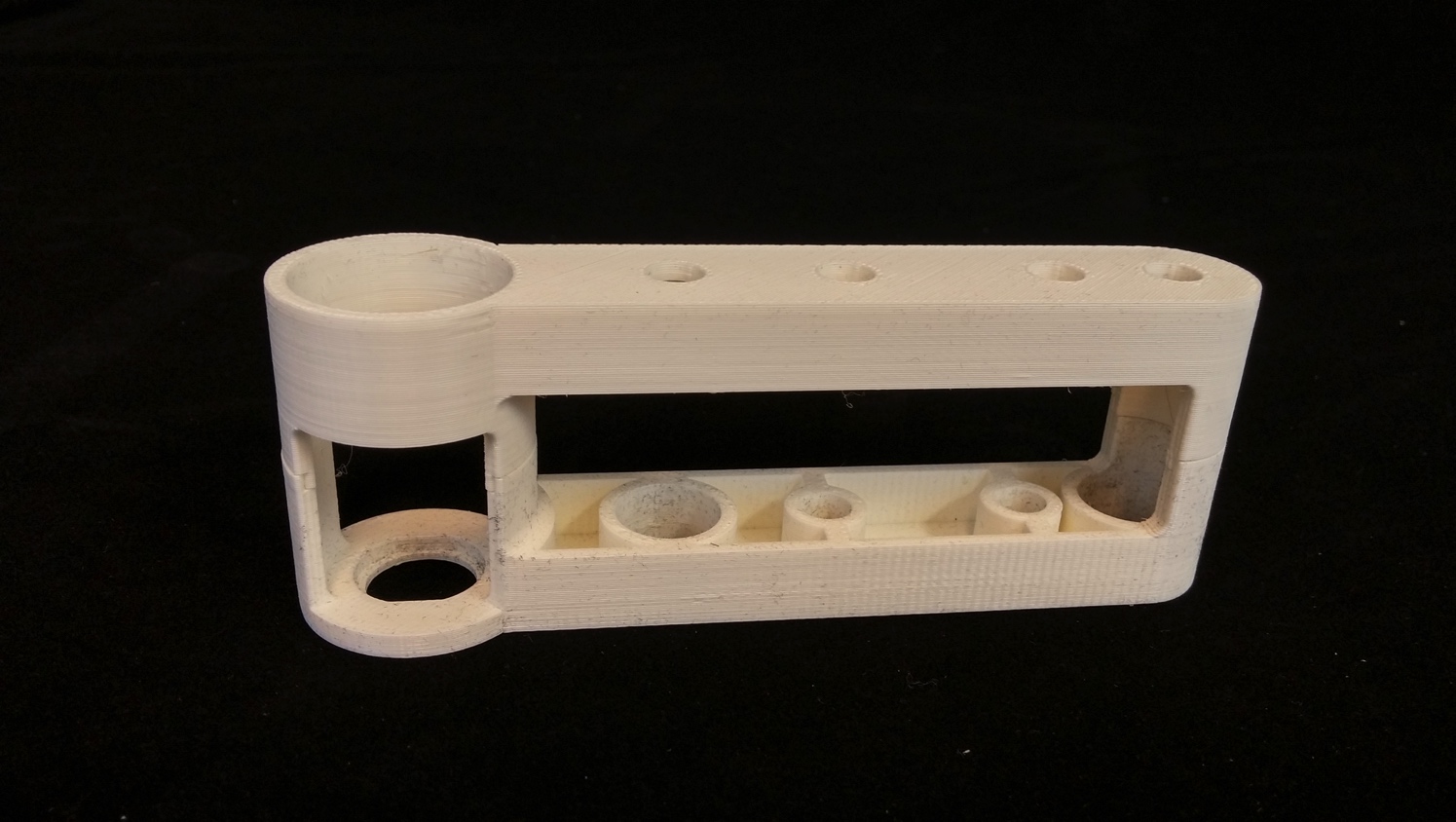
**

ii

**
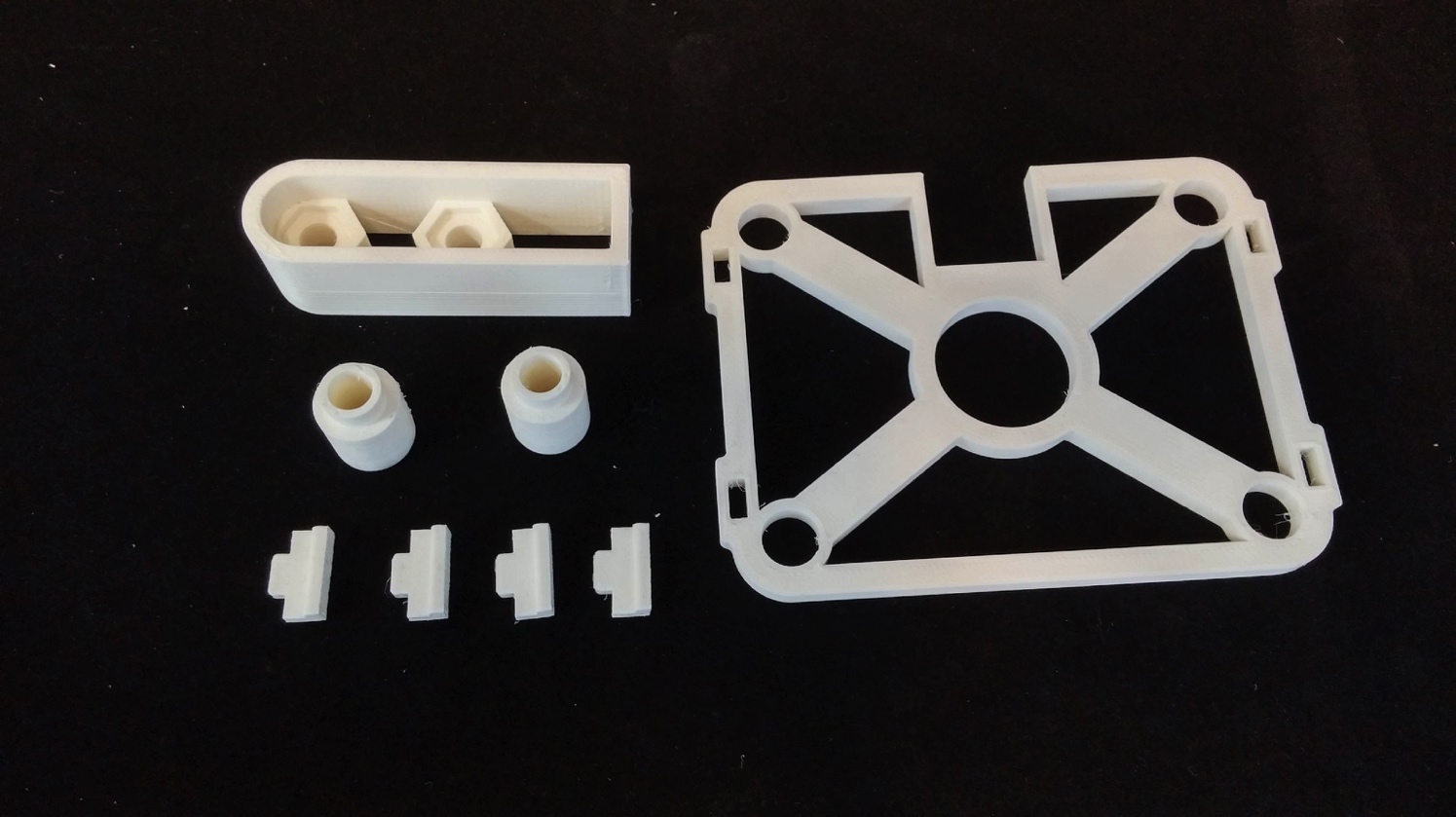
**

iii

**
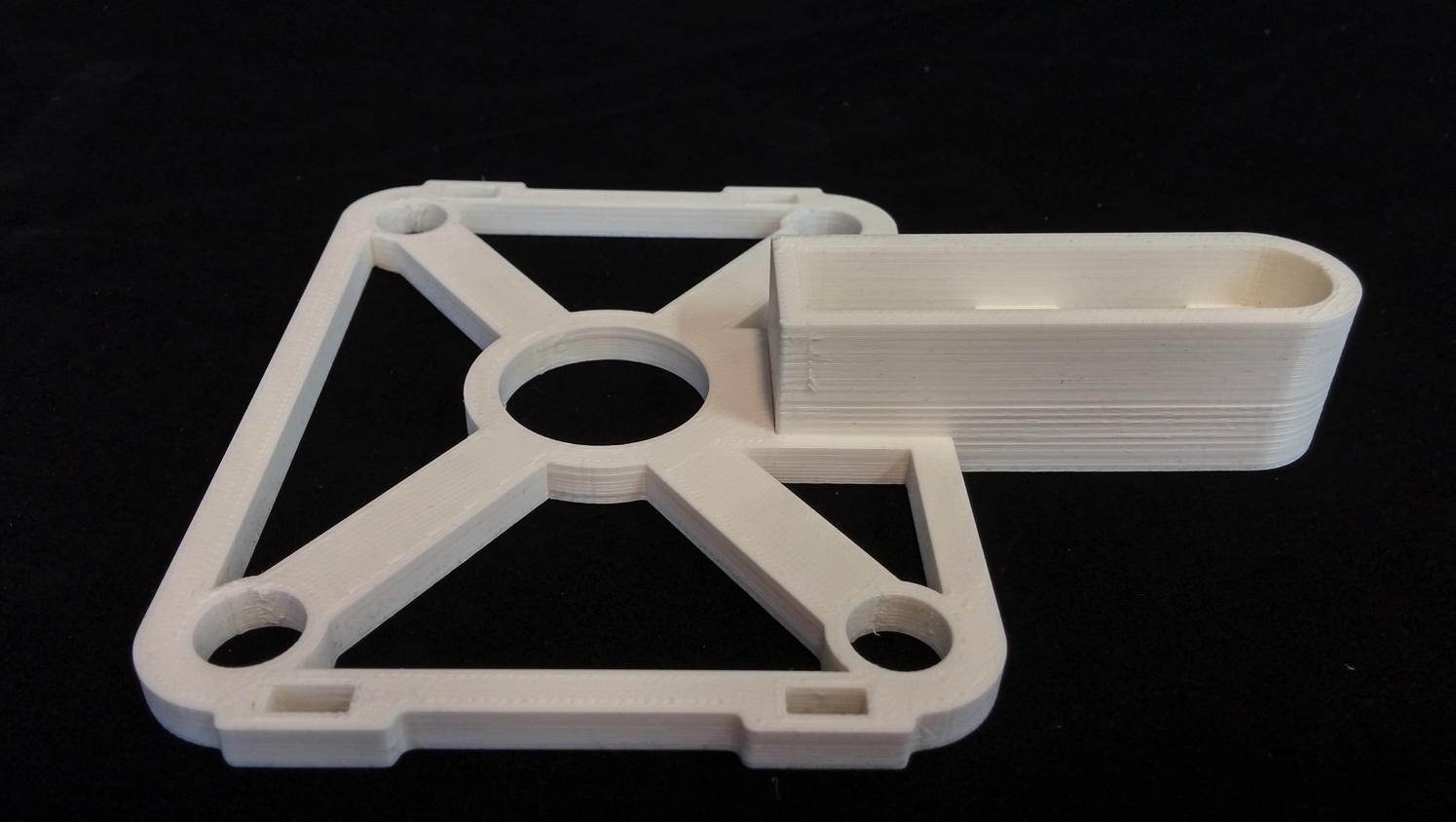
**

iv

**
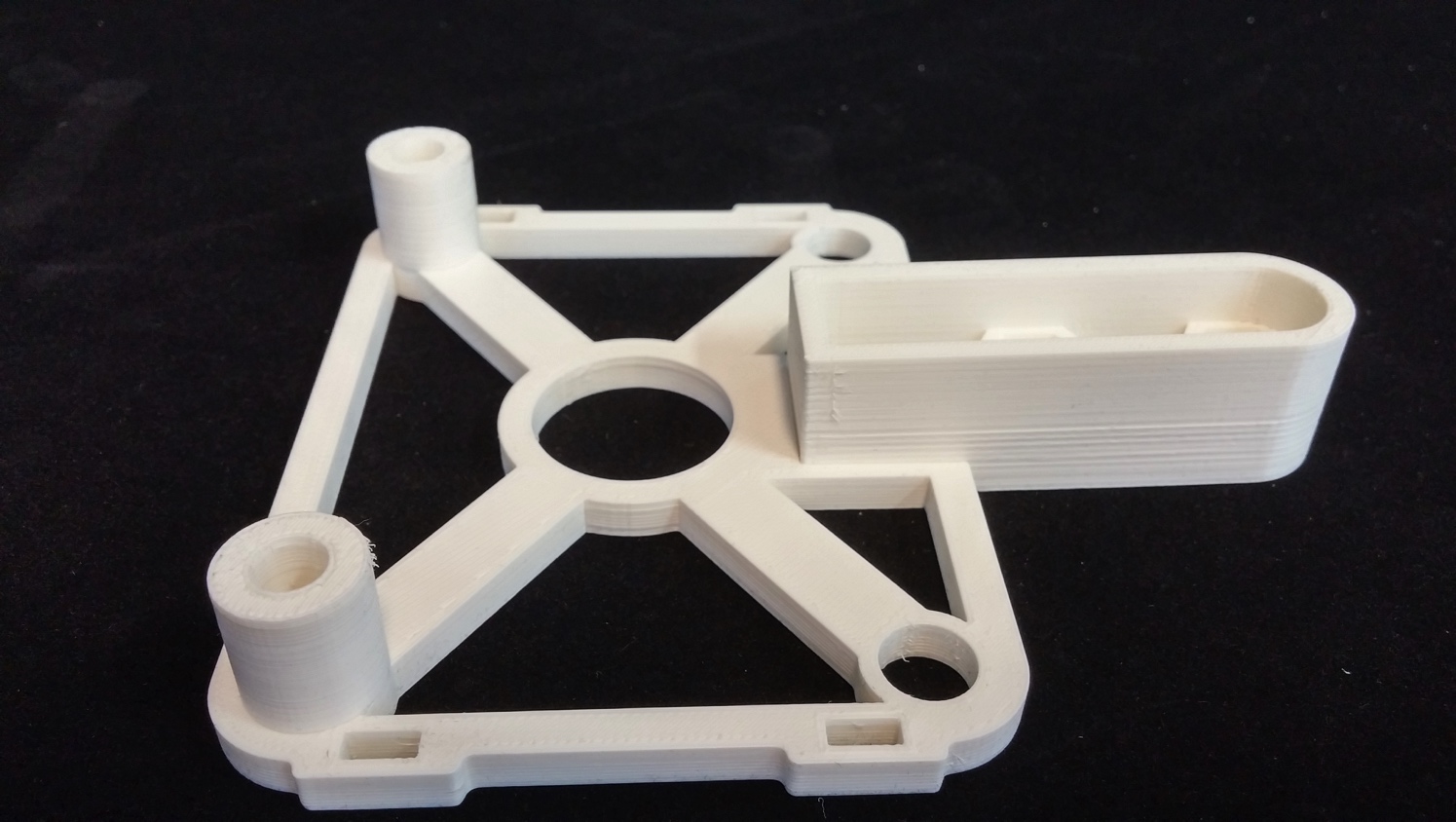
**

v

**
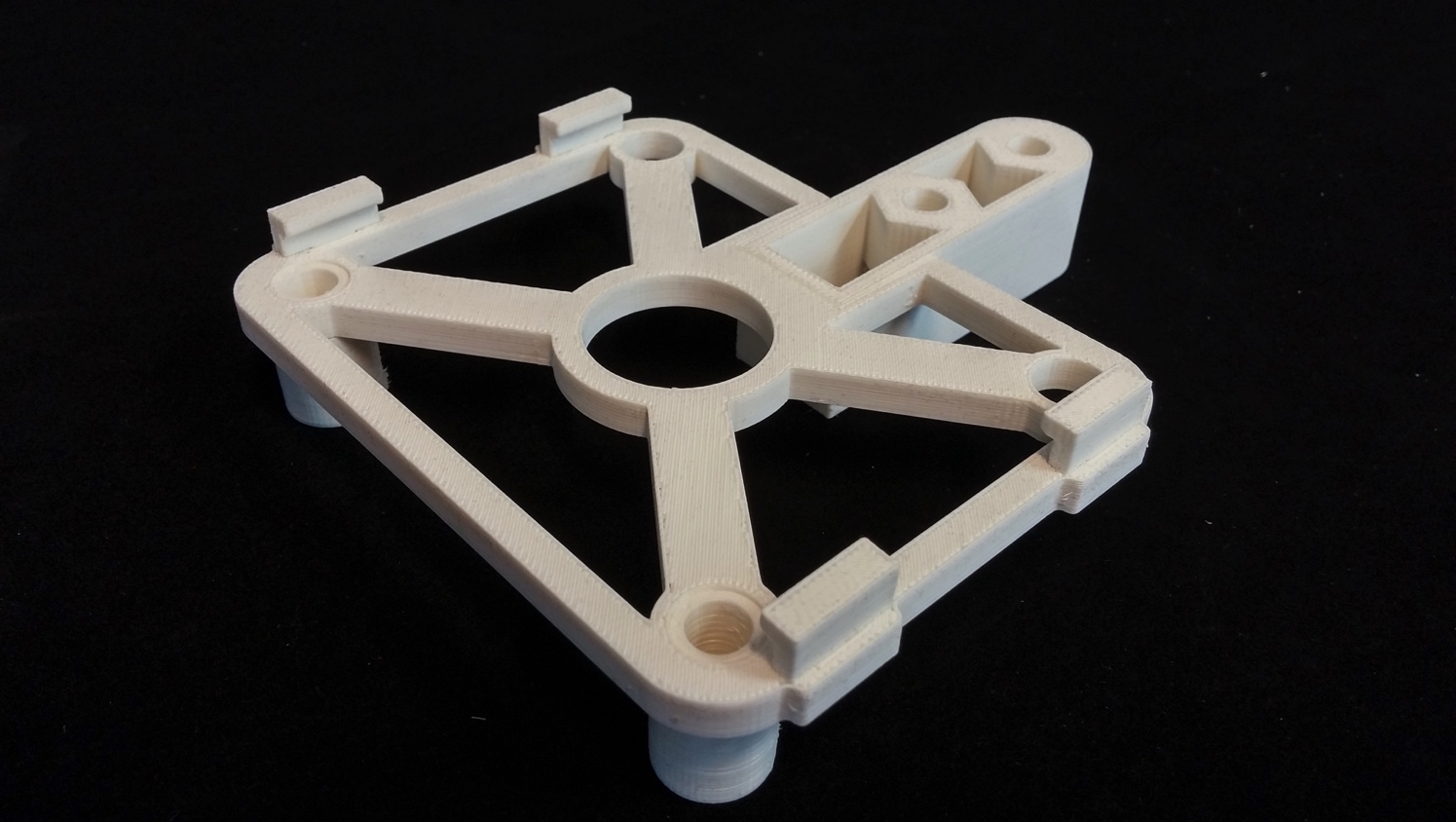
**

vi

**
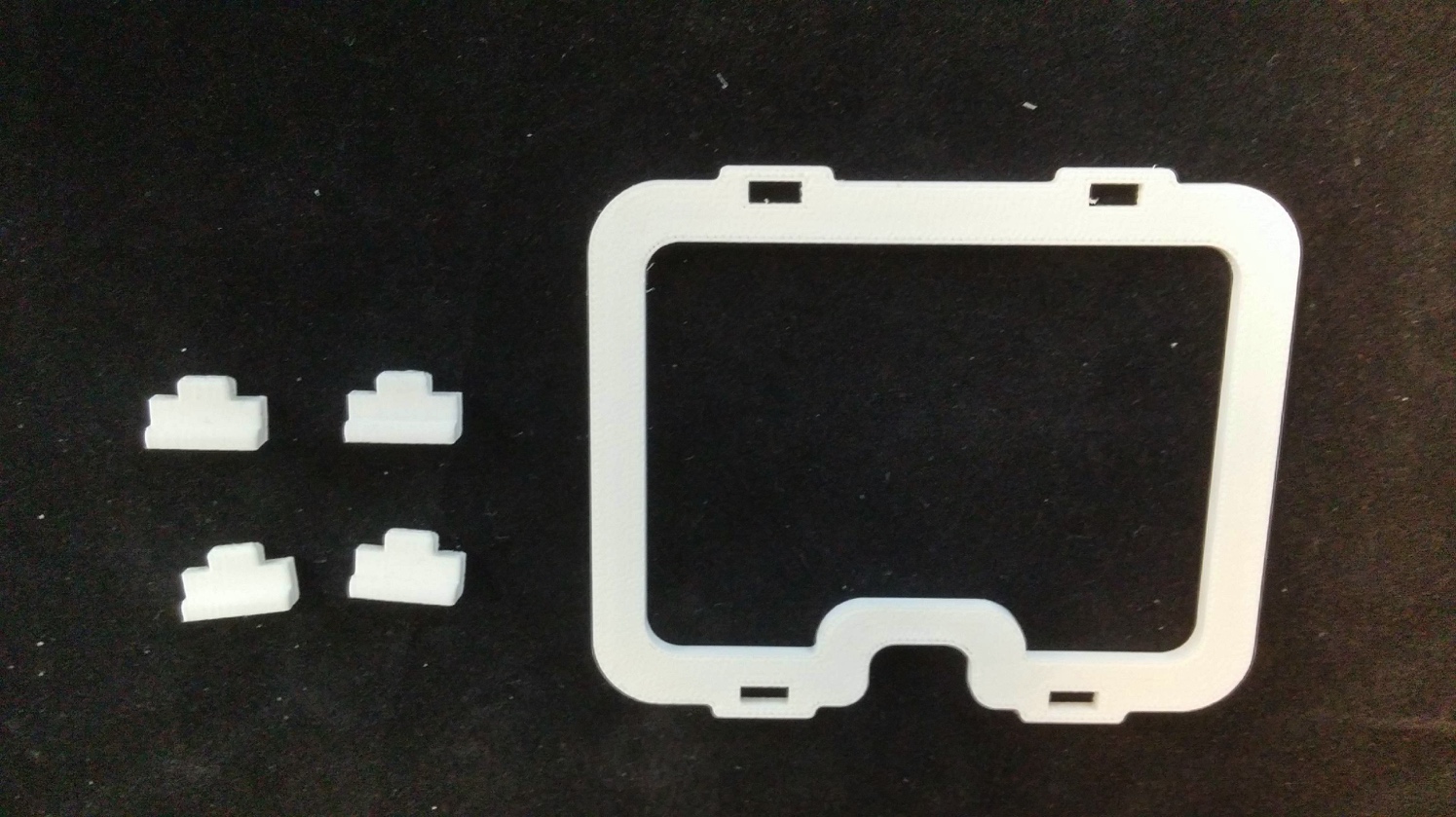
**

vii

**
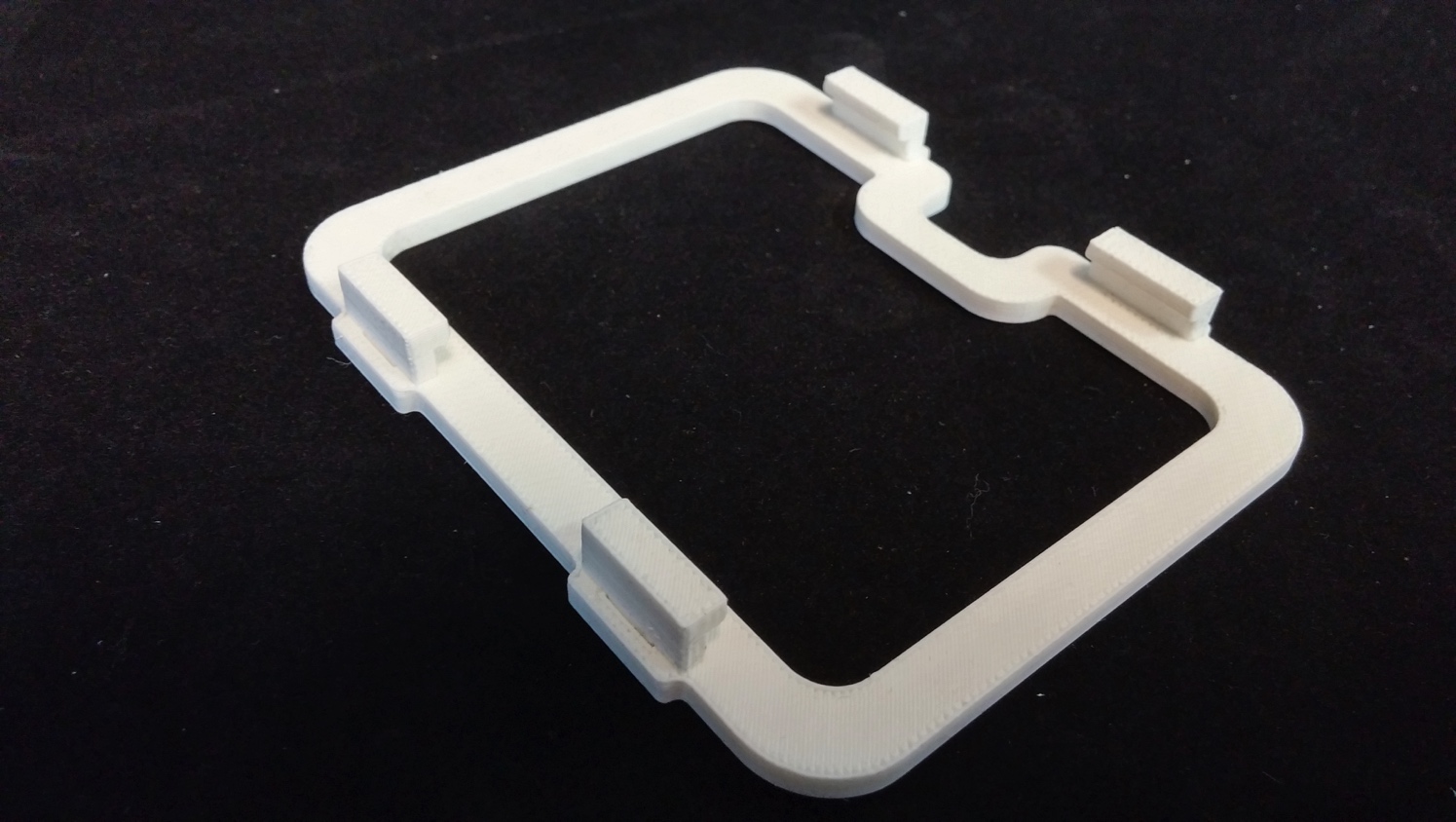
**

viii

**
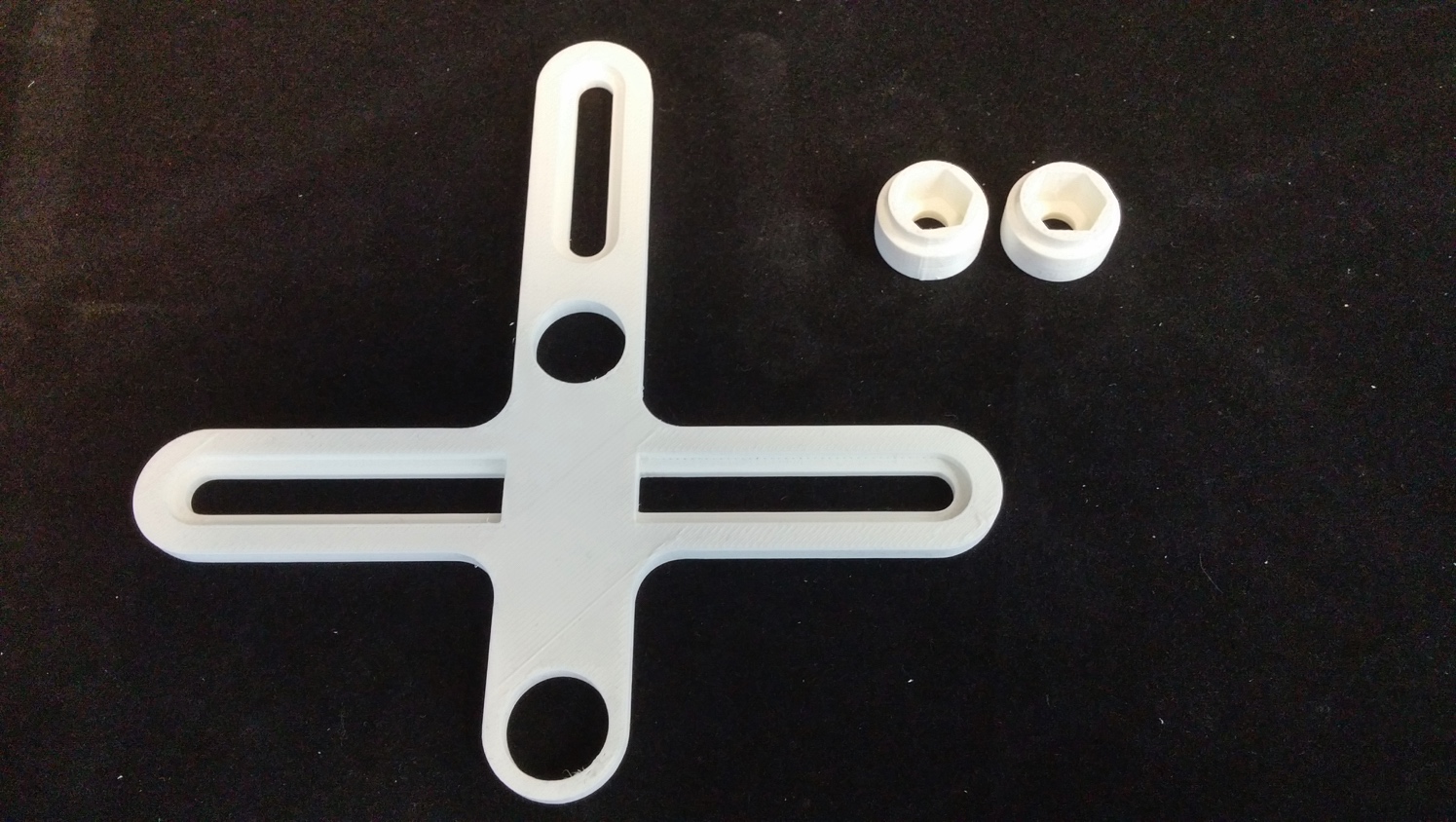
**

ix

**
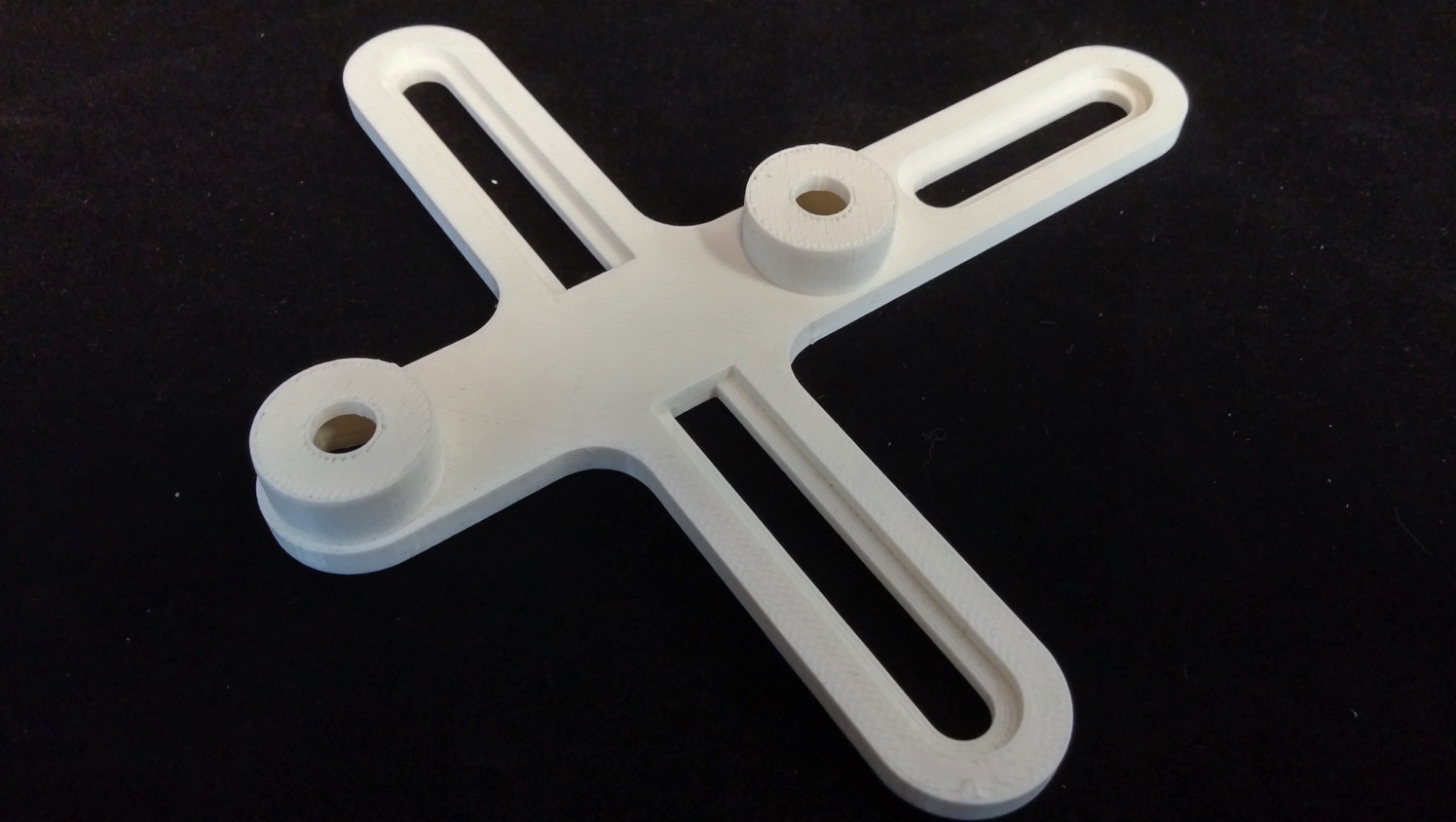
**

x

1. **Assembling the base:**

1


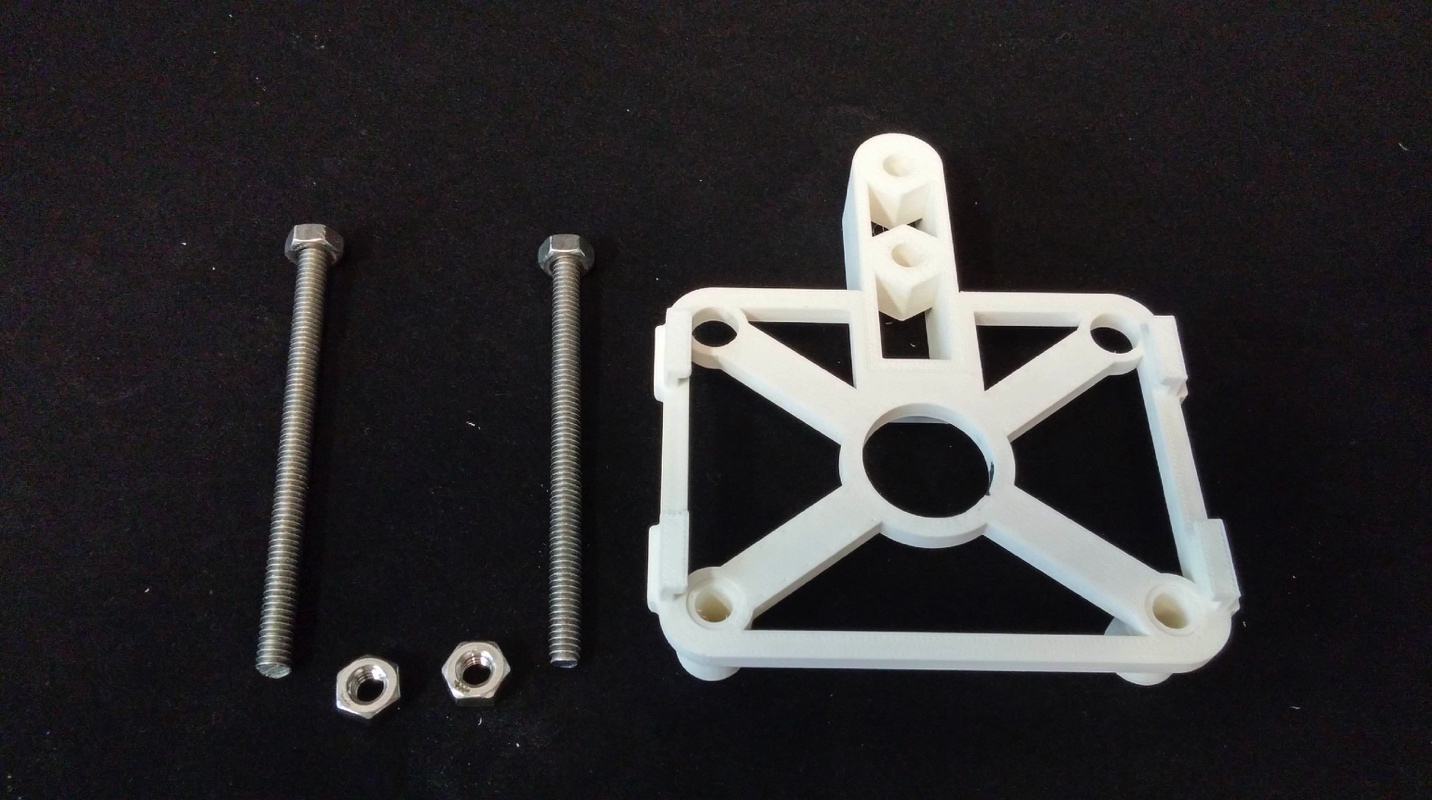


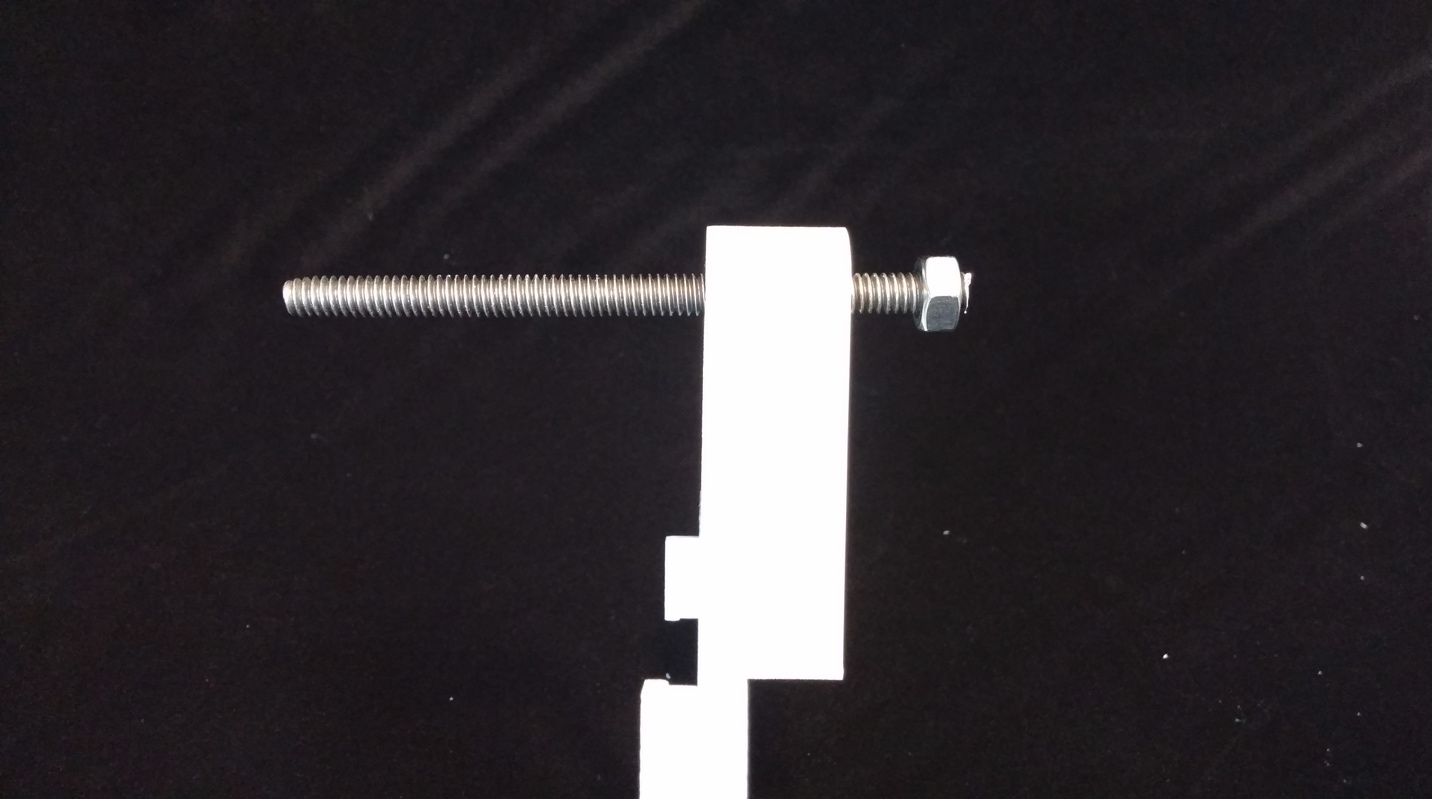


2


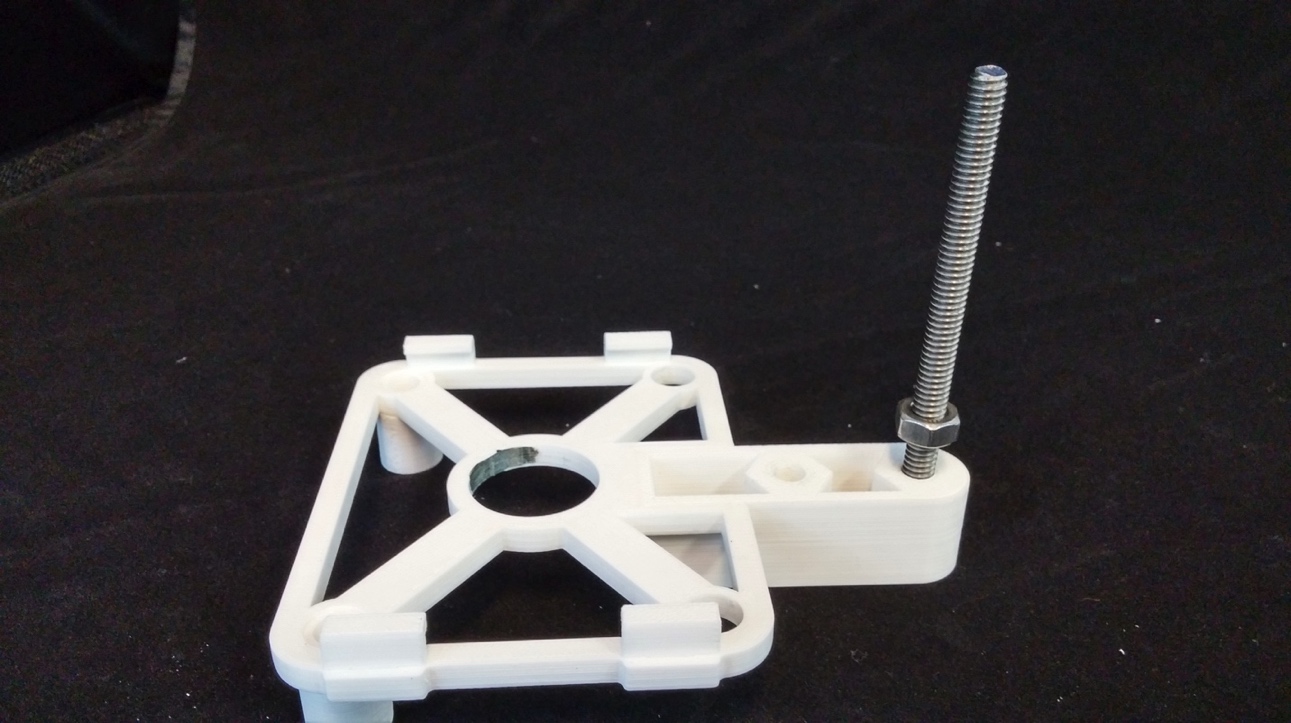


3


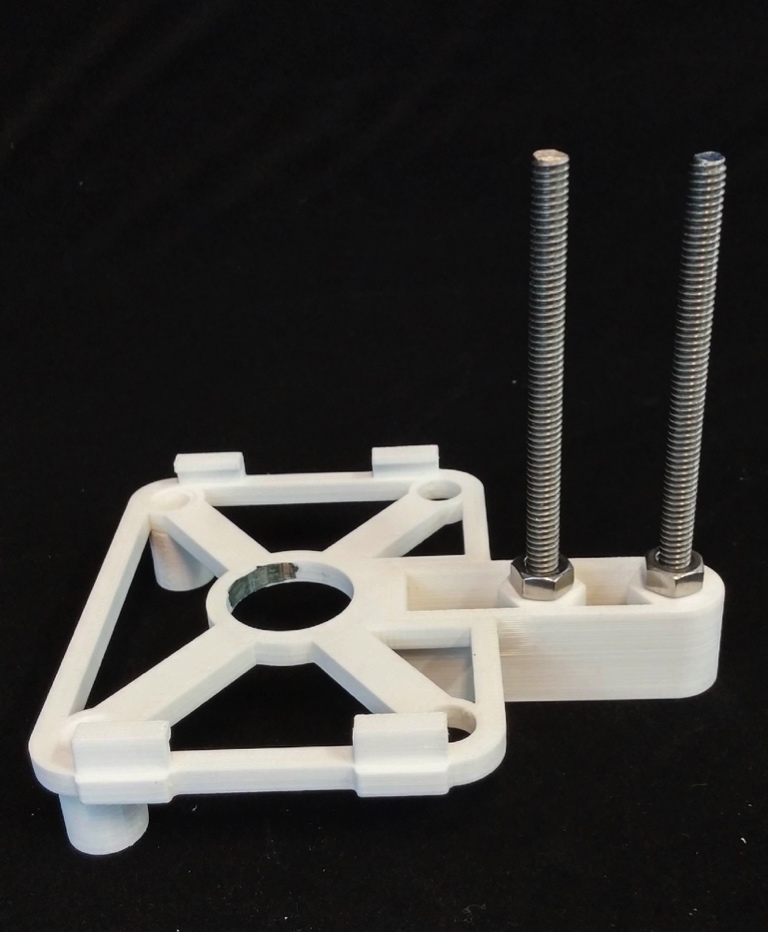


4

Make sure that the screws do not stick out at the base.

1. Sliding platform and illumination:

5


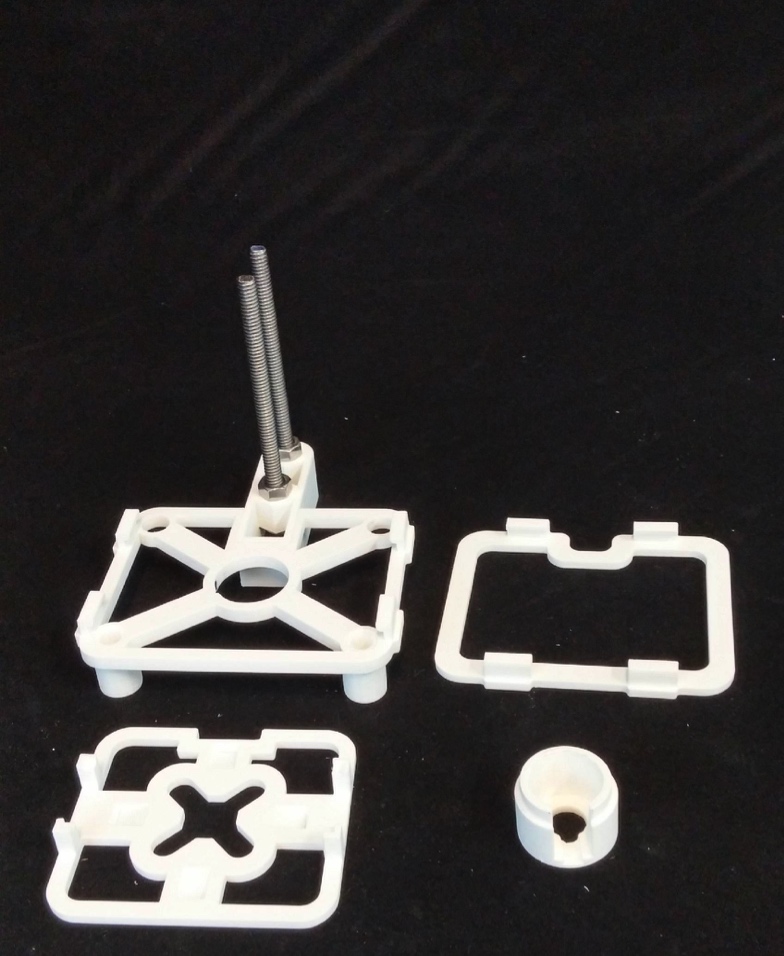


**LED holder**This part is also used in the circuit building instruction. If the circuit was built first, the LEDs are already attached to this piece.


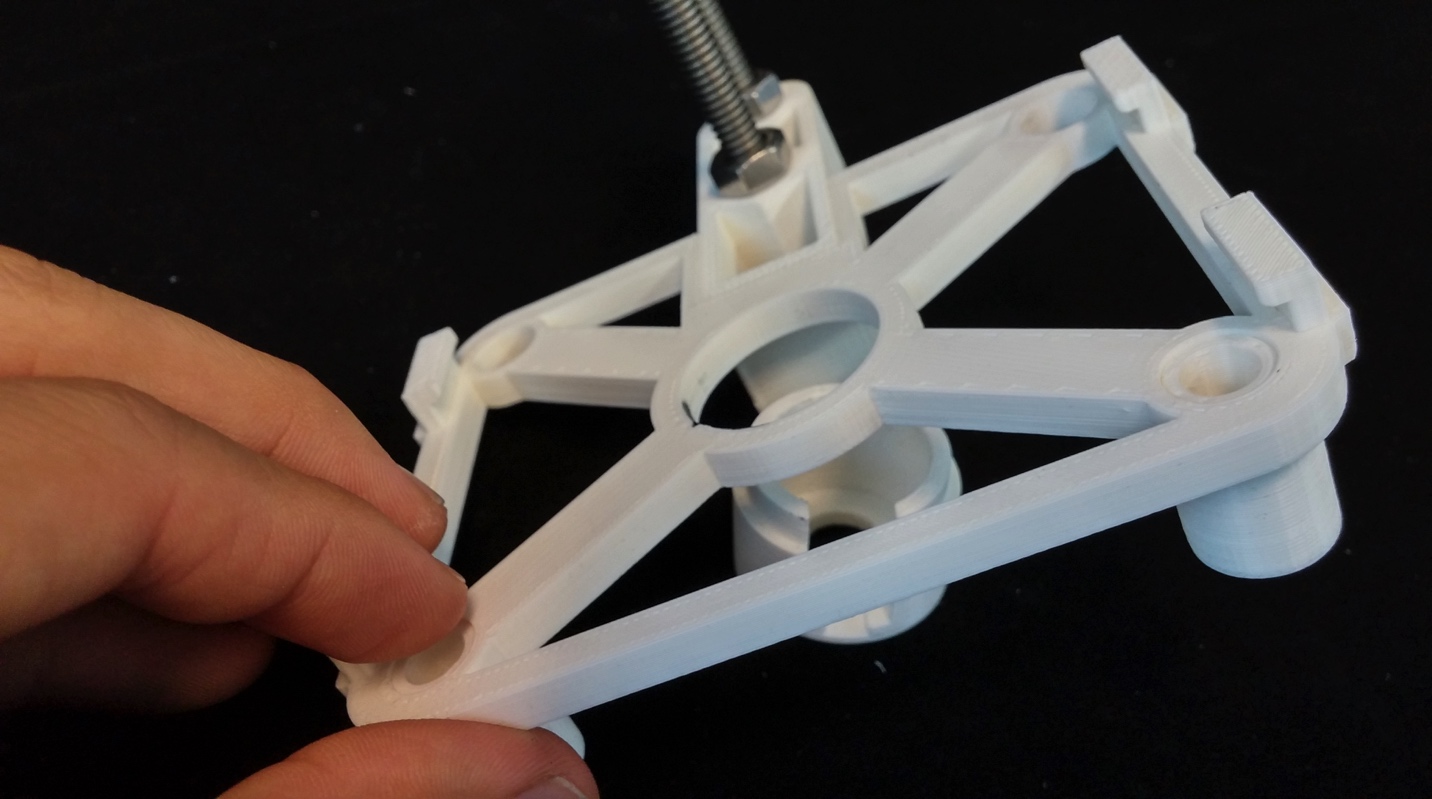


7

6


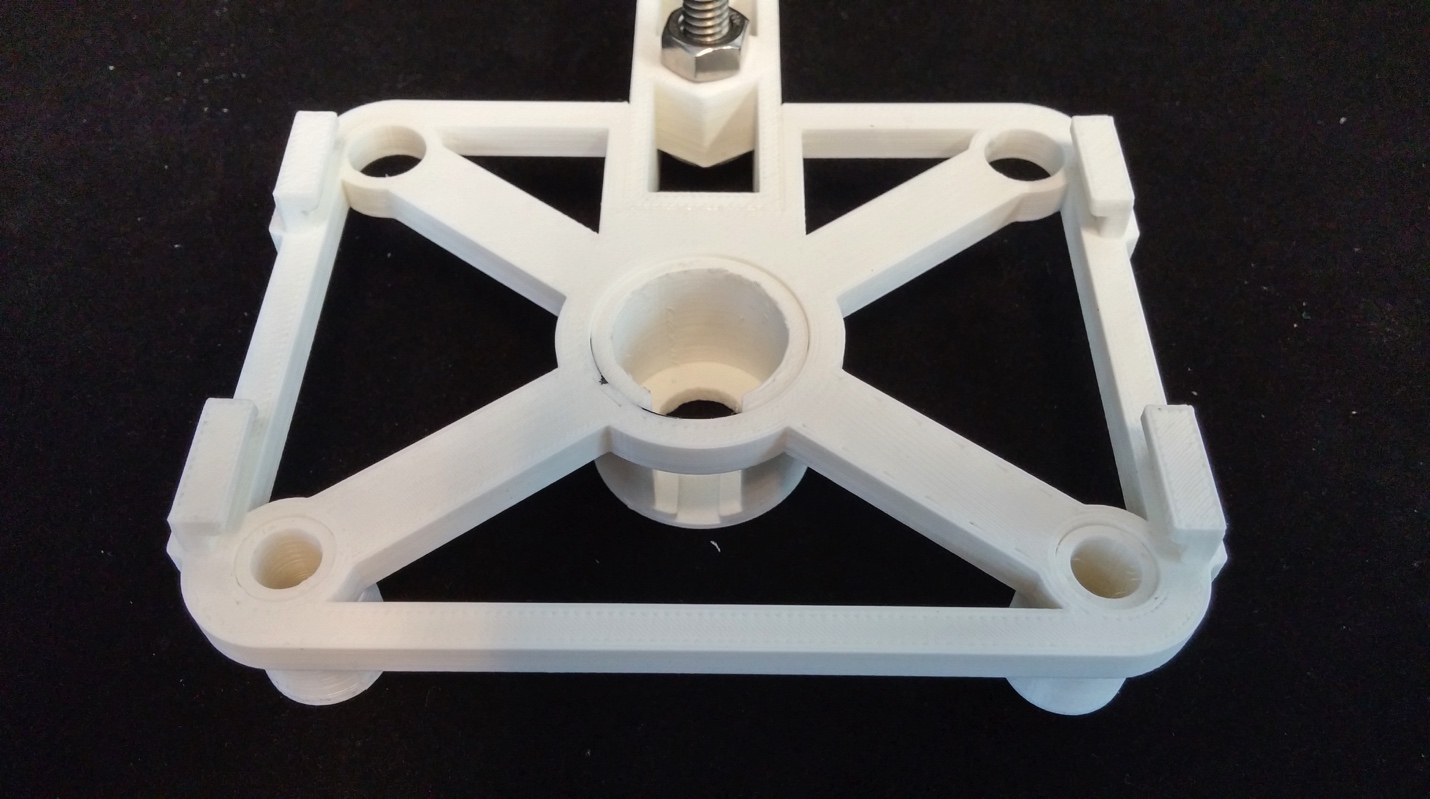


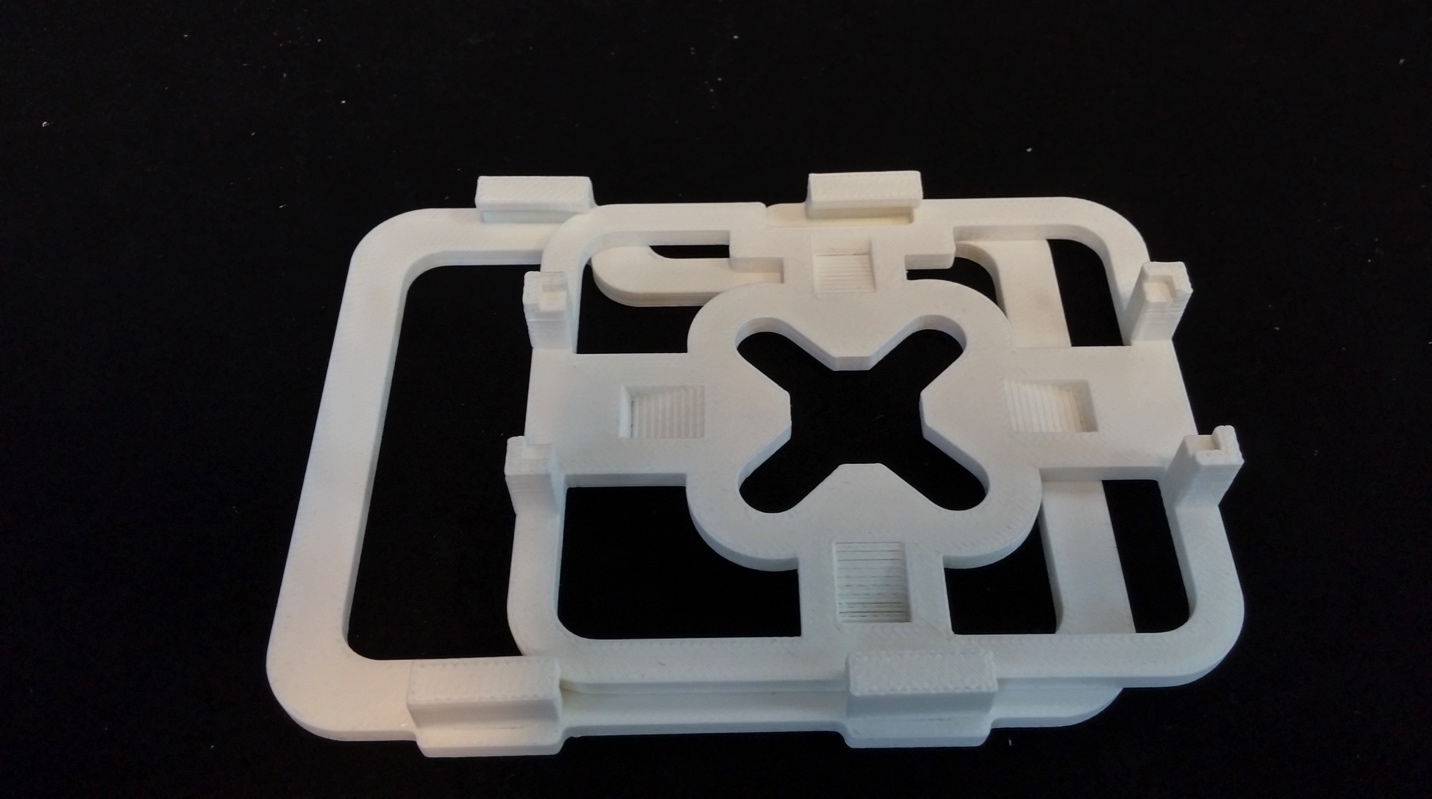


9

8


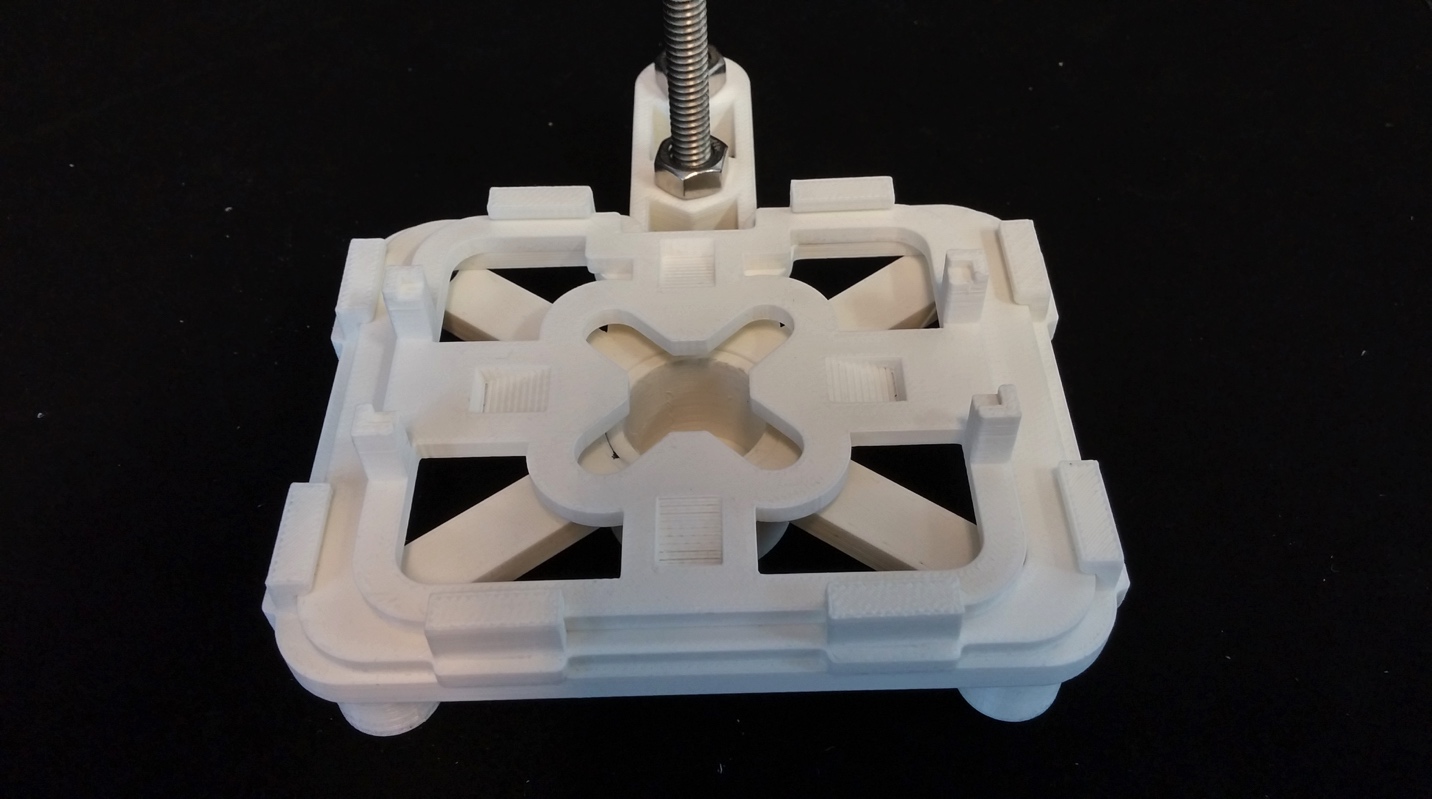


1. Assembling focus knob:

10


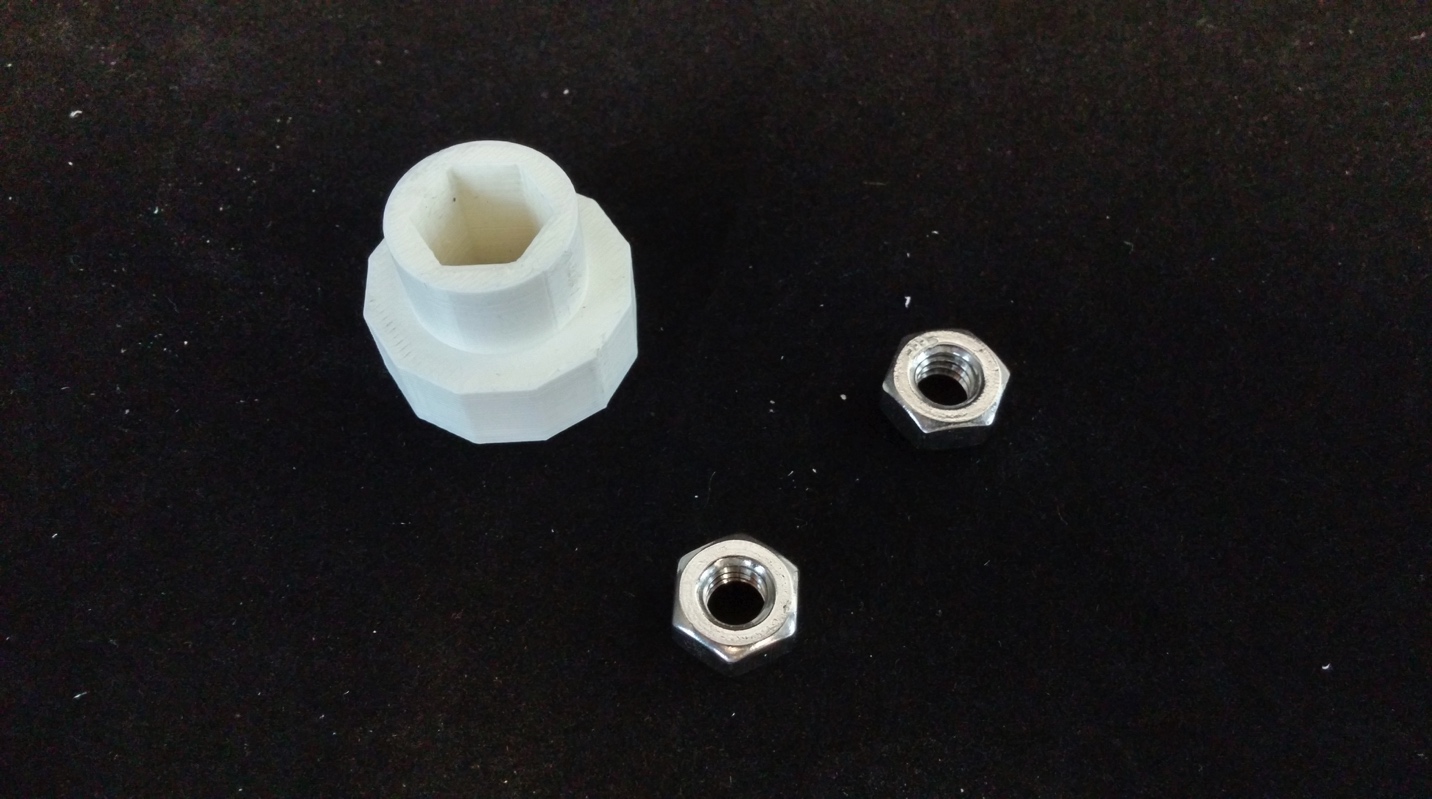

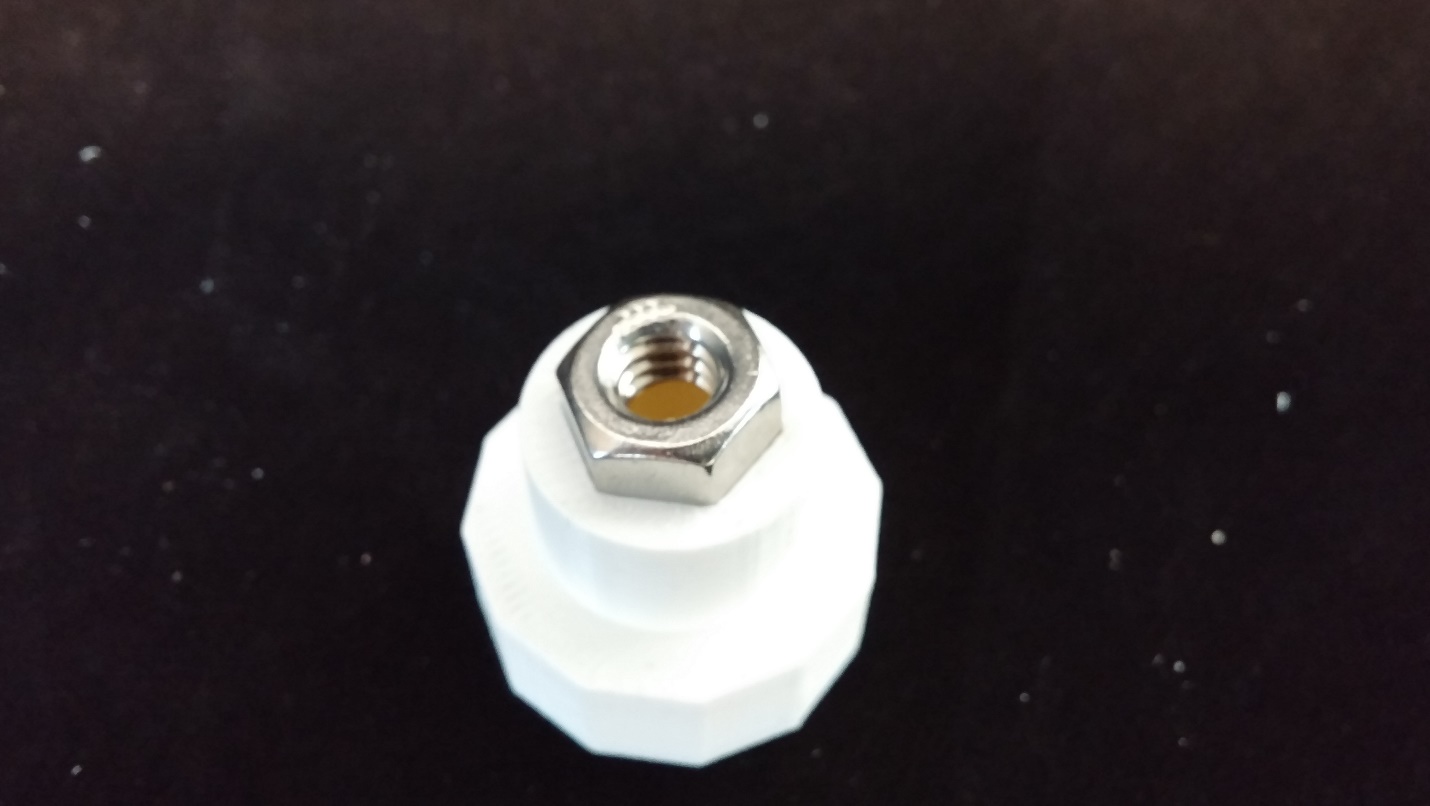


11


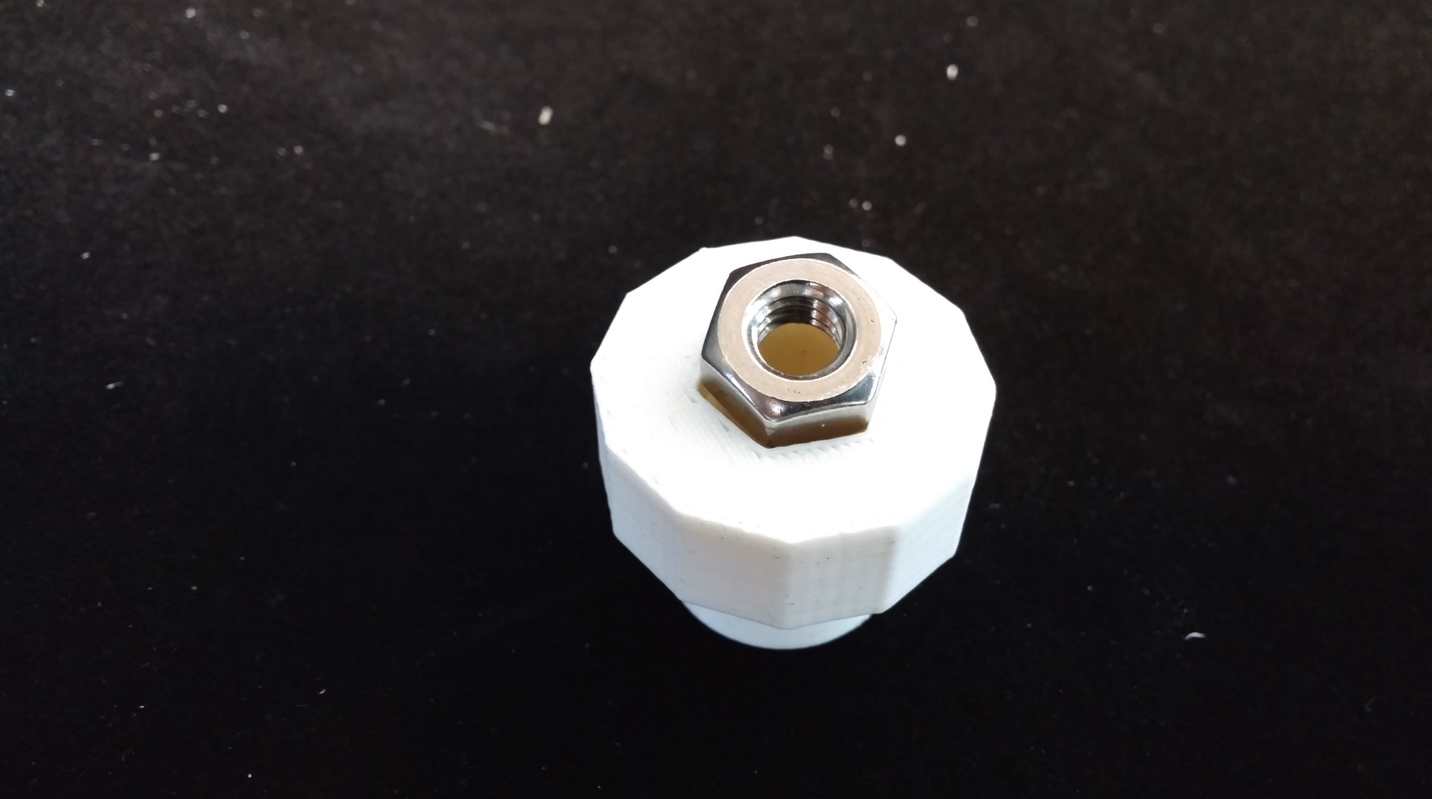


12

Press in the two nuts. After inserting the two nuts, they should be flush with the surfaces.


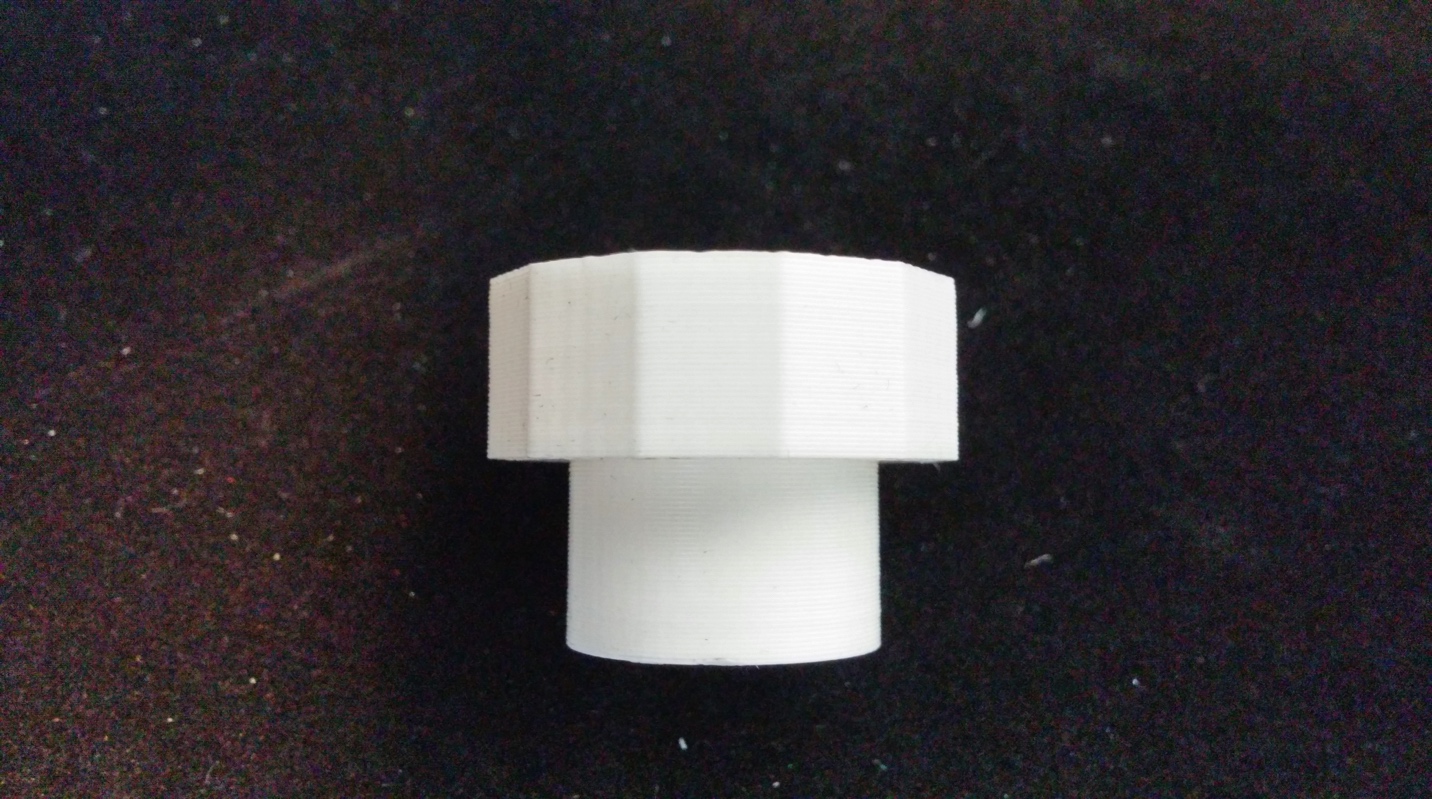


13

1. Assembling optics tube:


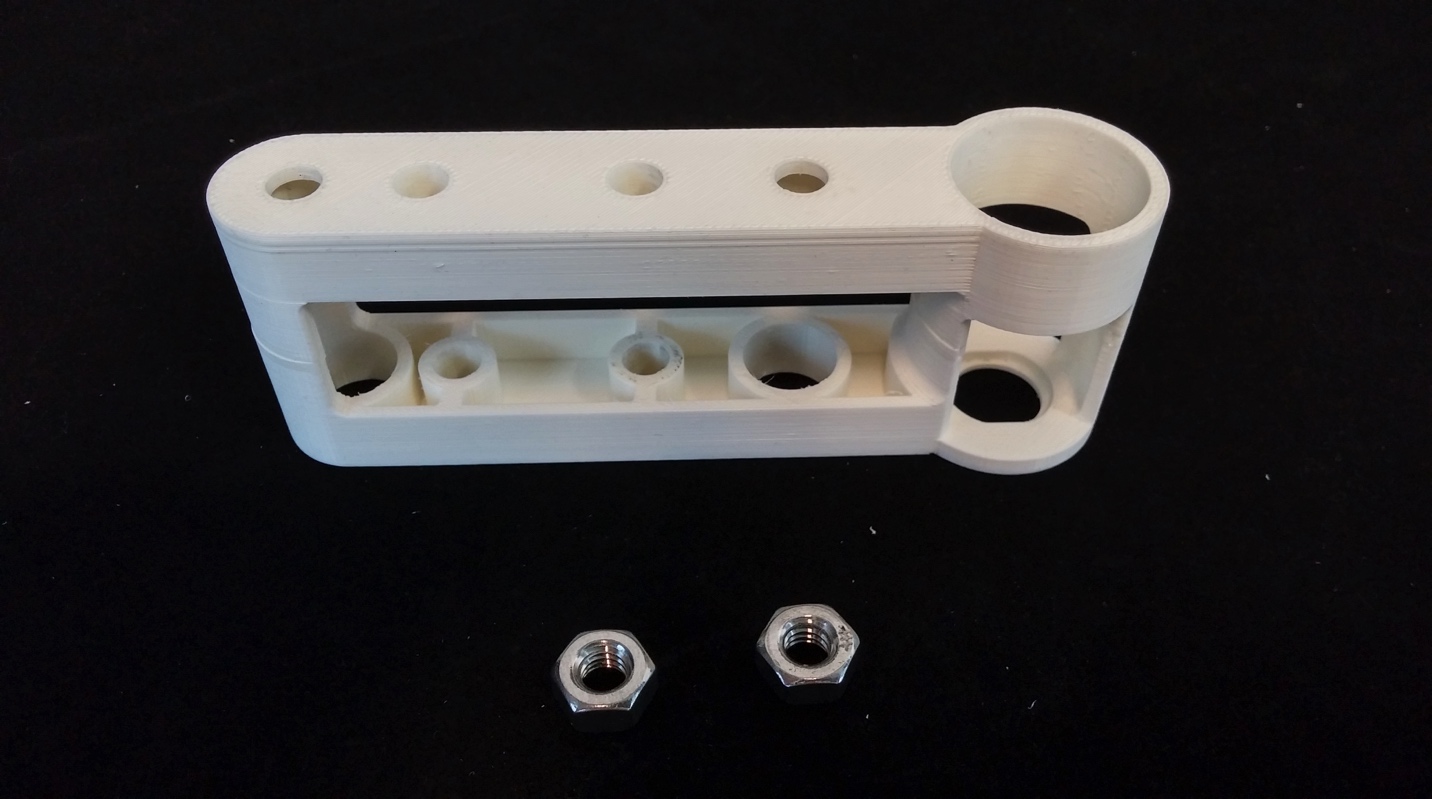


14


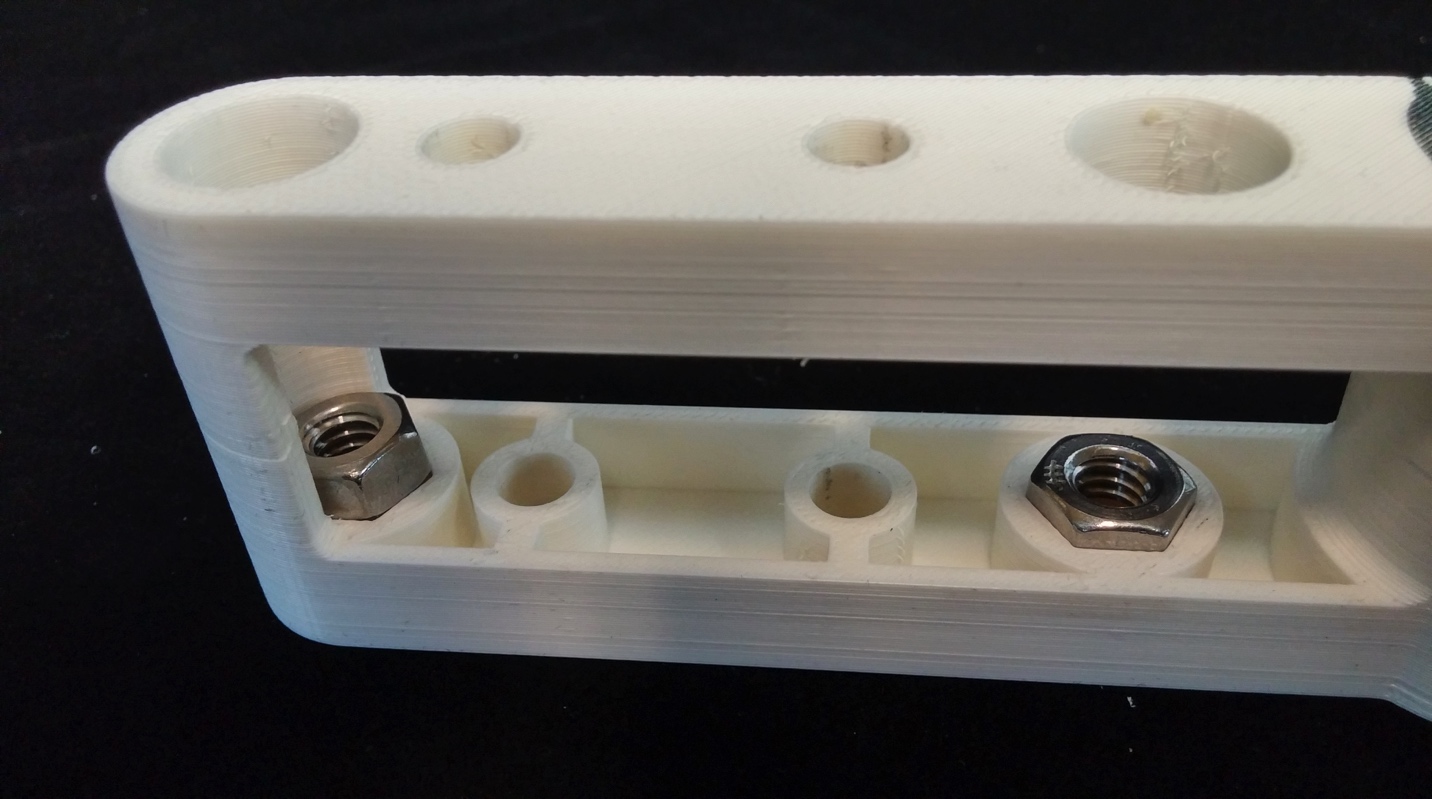


15


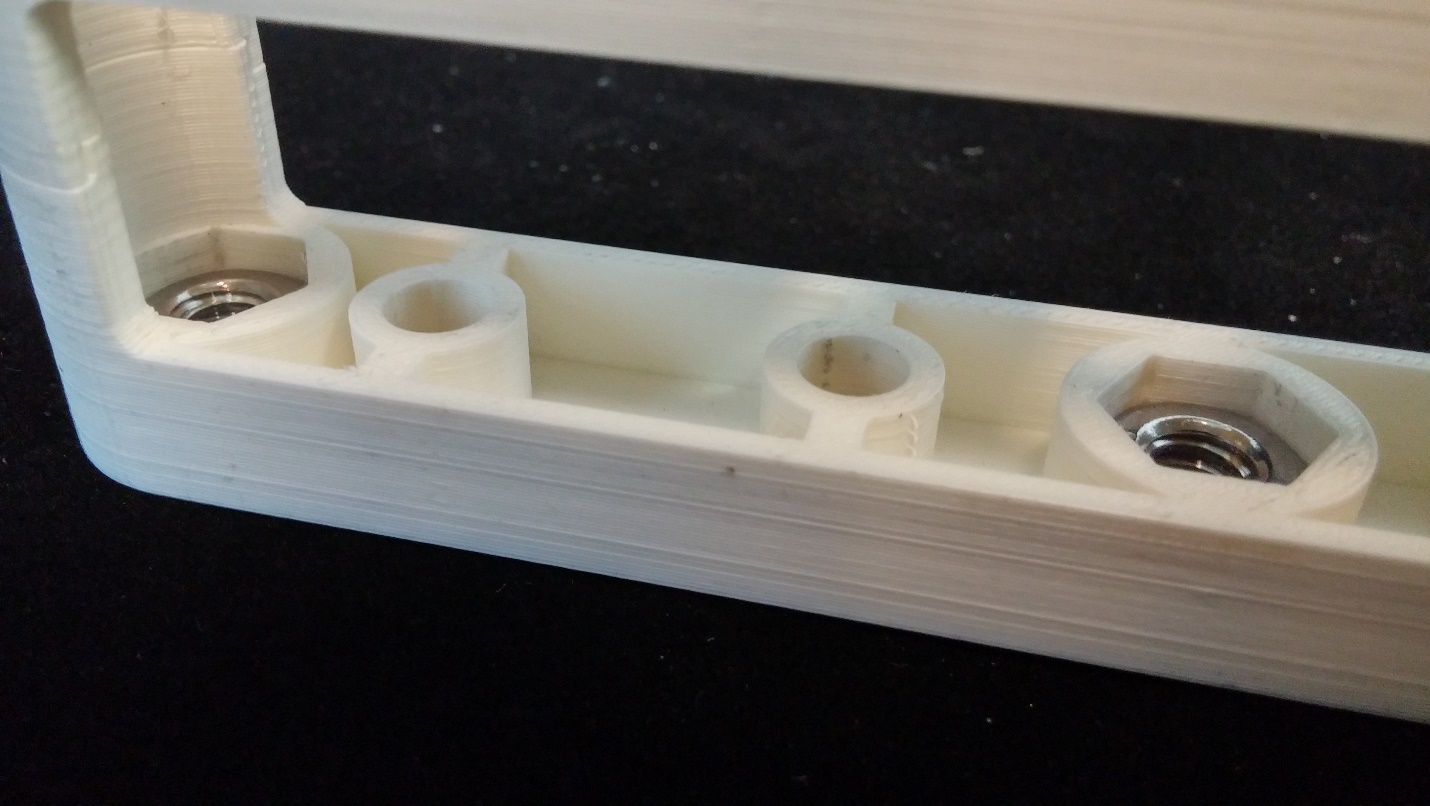


16

1. Putting it all together:


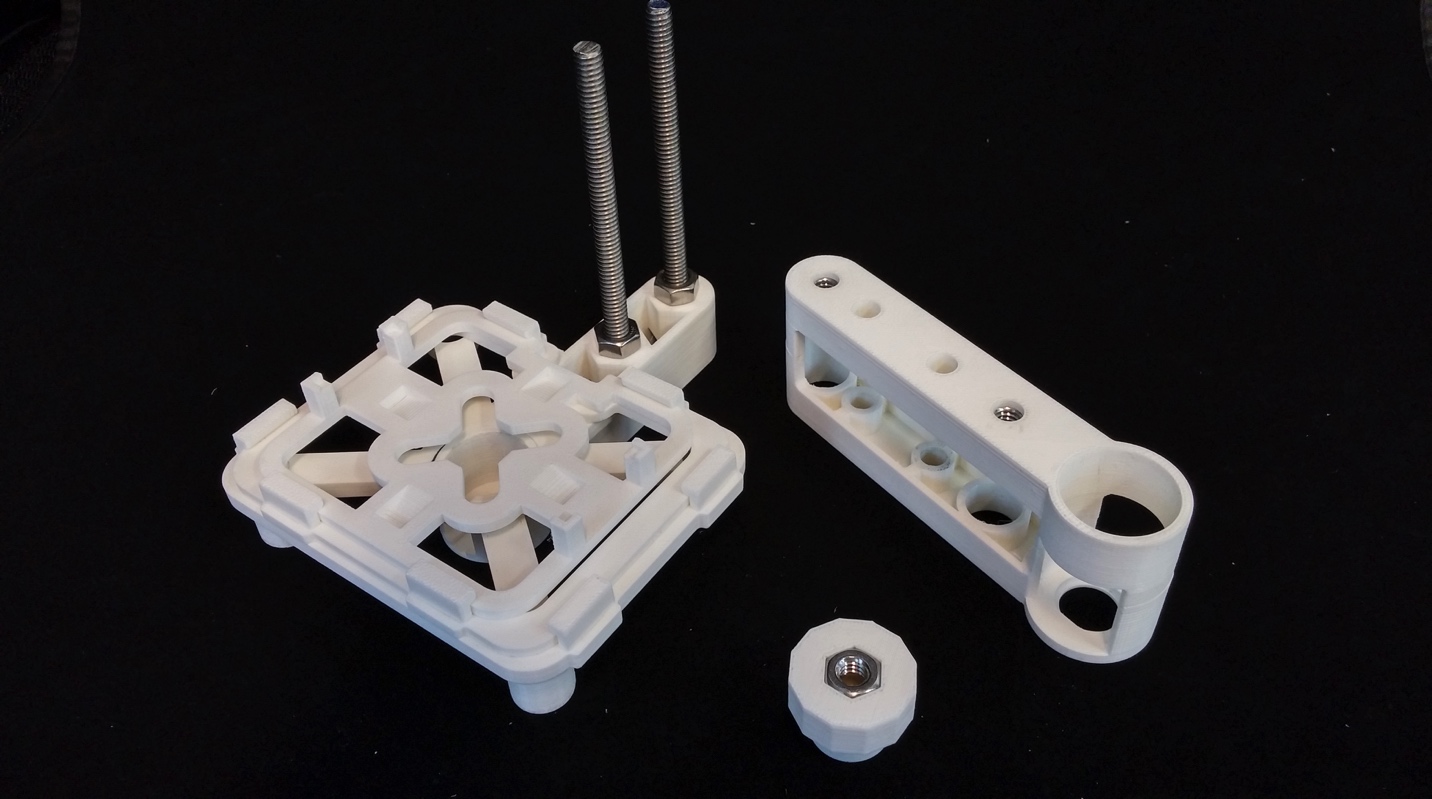


17


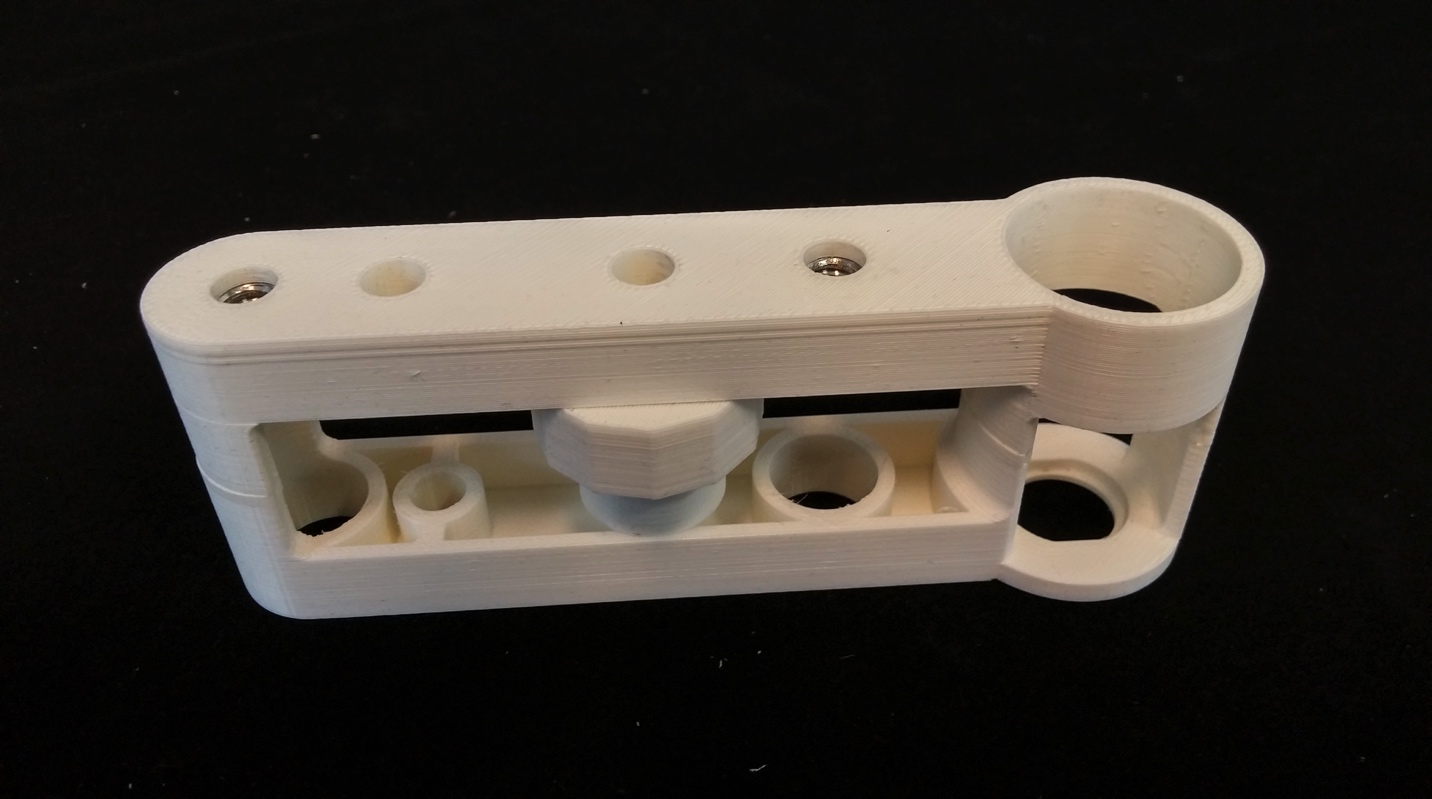


18


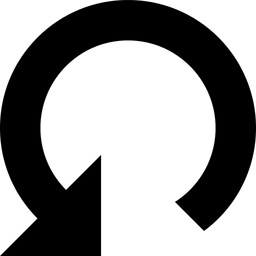

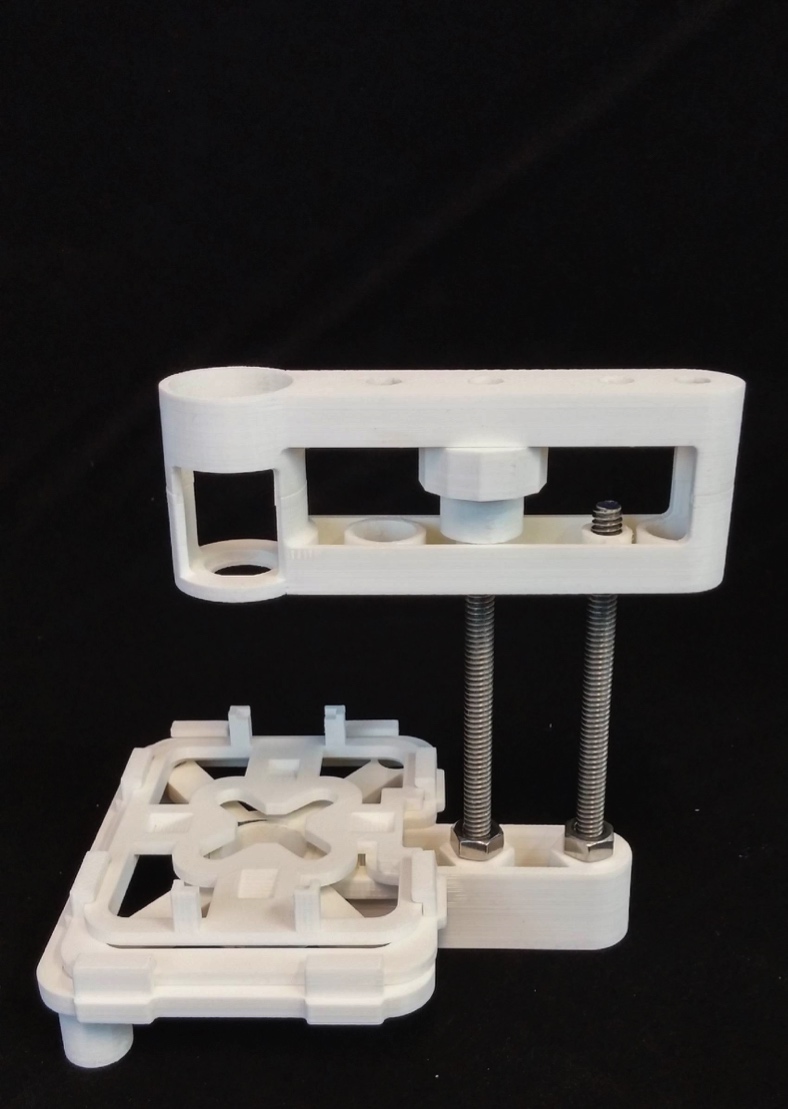


Turn knob clockwise (from top) to screw the parts together until both screws come out on top. (If this is hard, the two nuts in the knob might not be at optimal distance – see image 12. In that case adjust the distance between the nuts.)

19


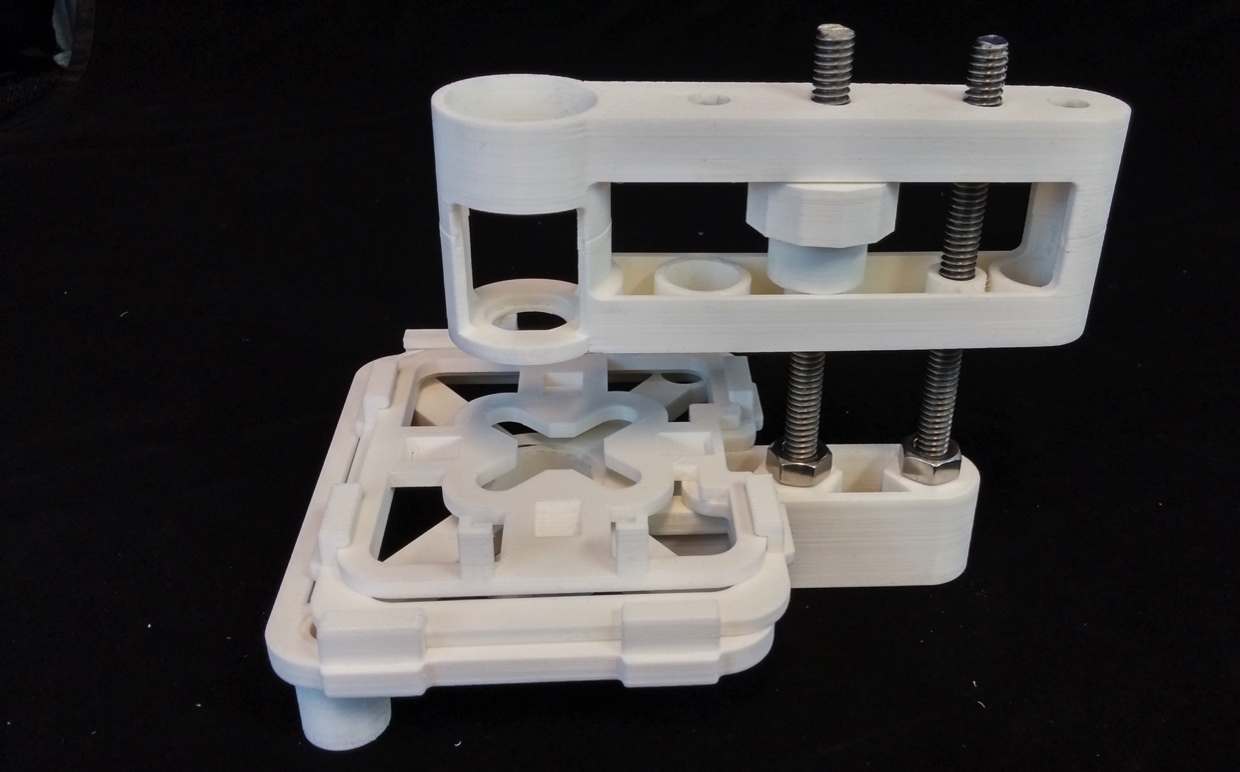


20

1. Assembling phone holder:

21


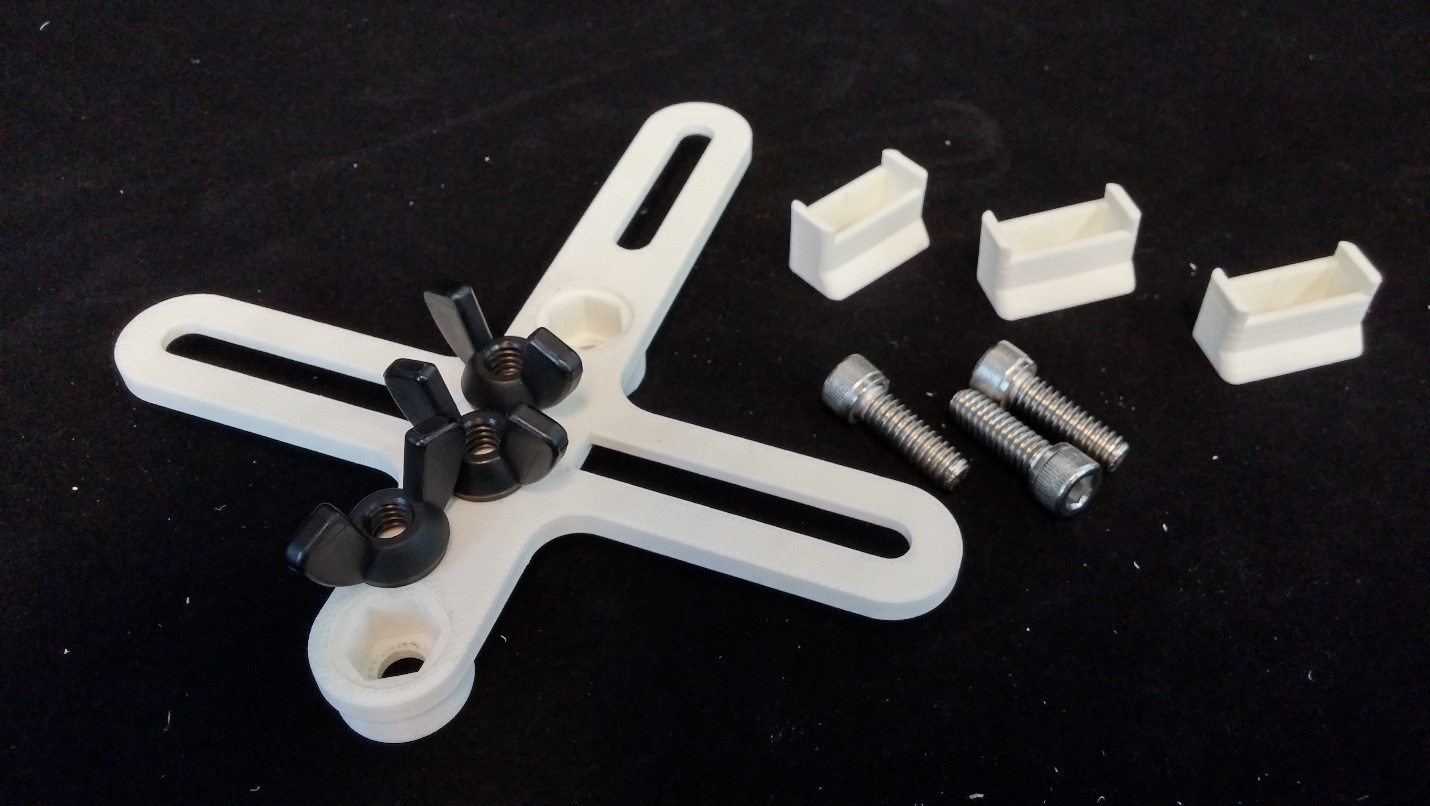


22


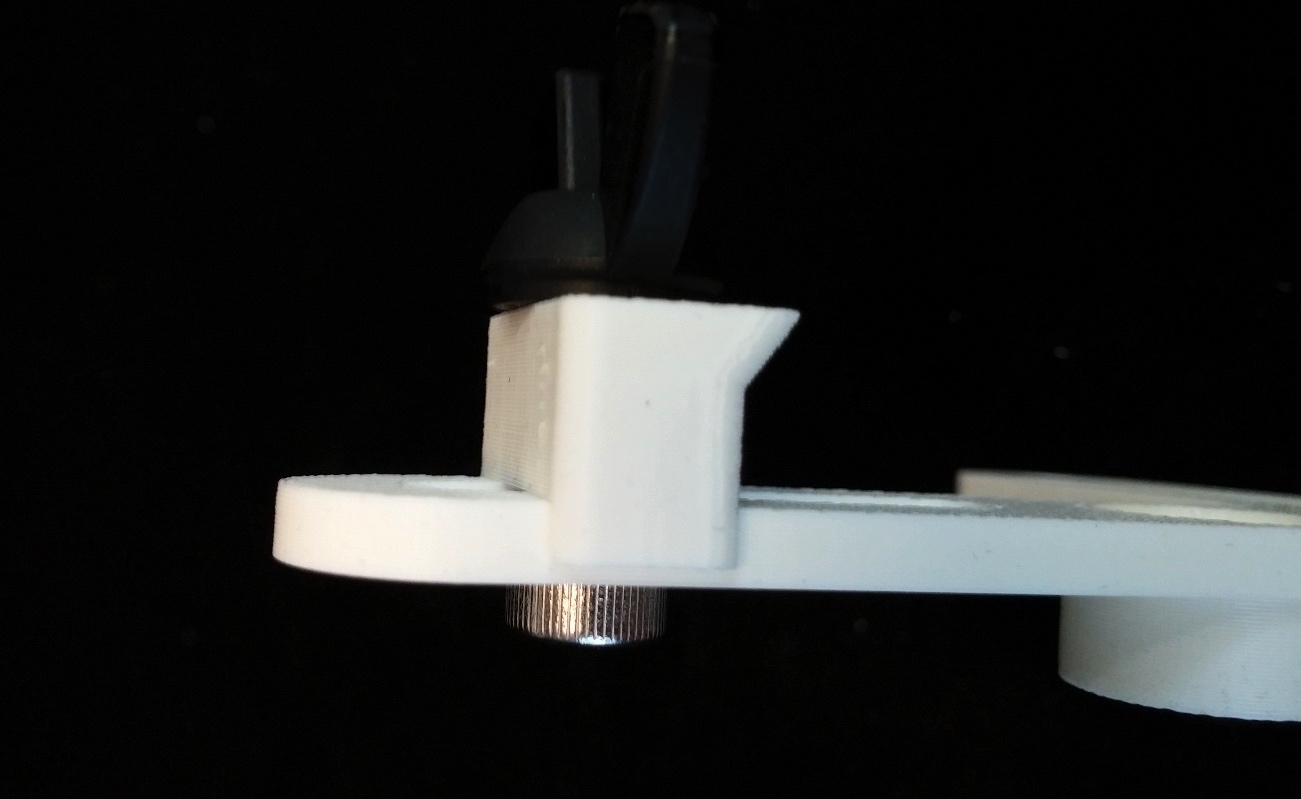


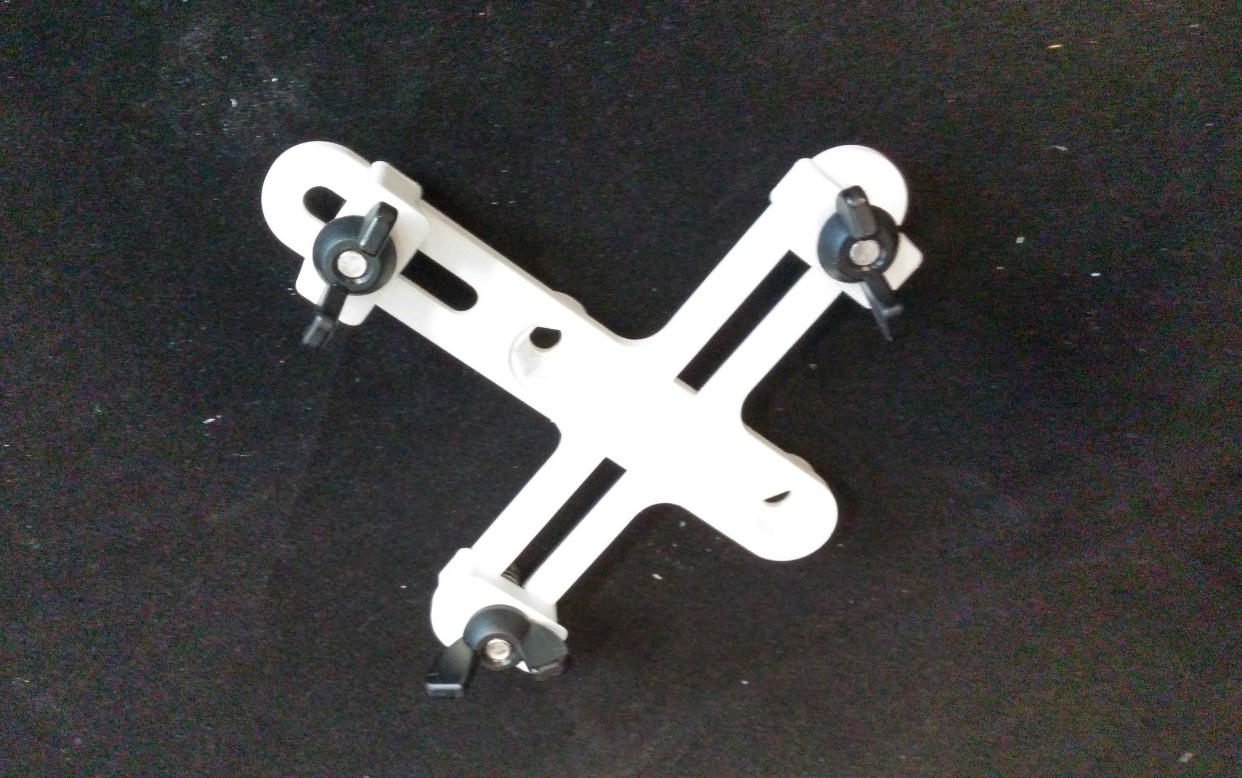


23

24


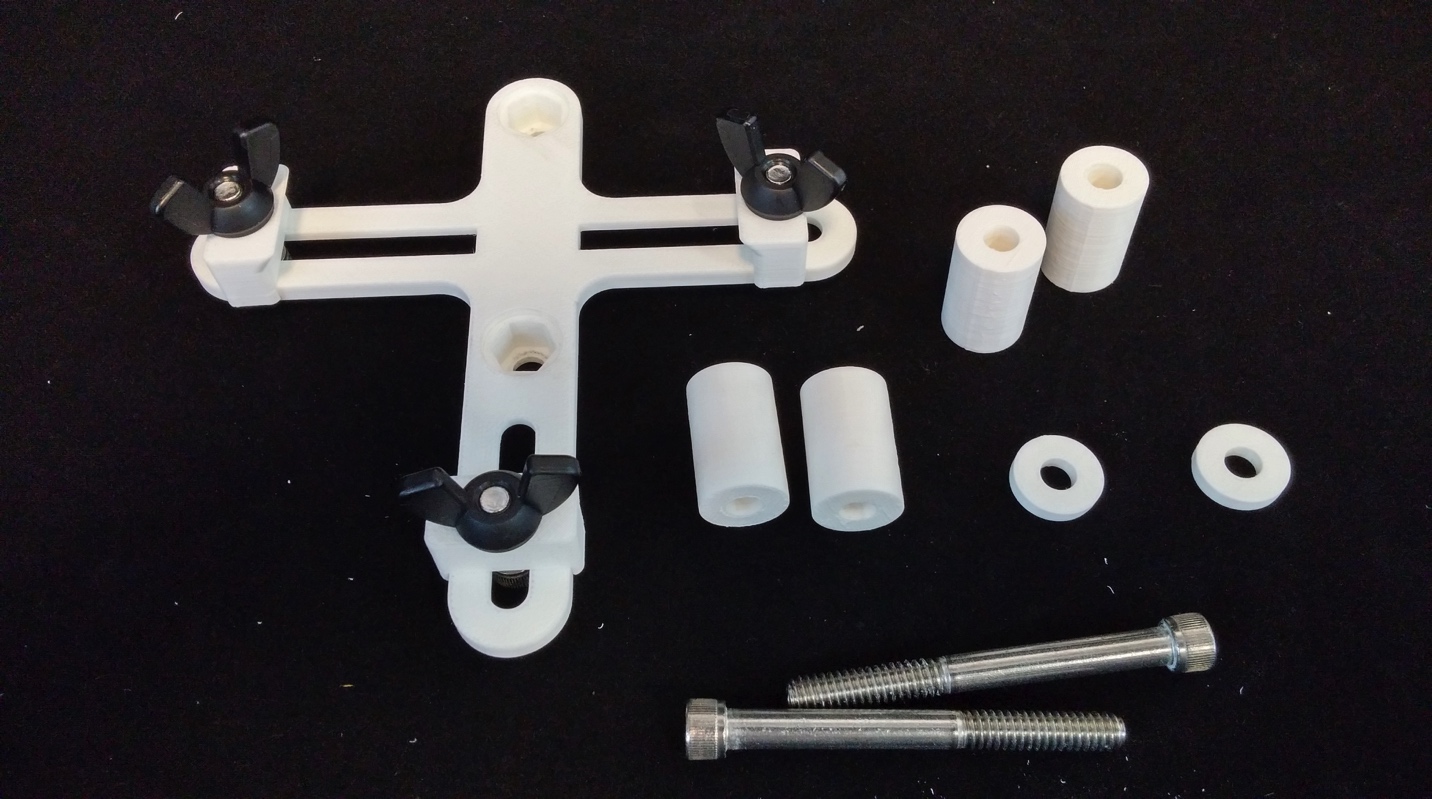


**Spacer Tubes**


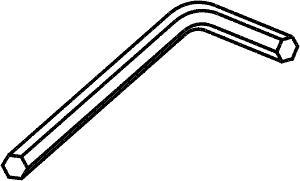

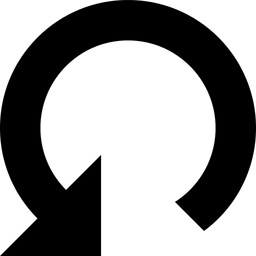

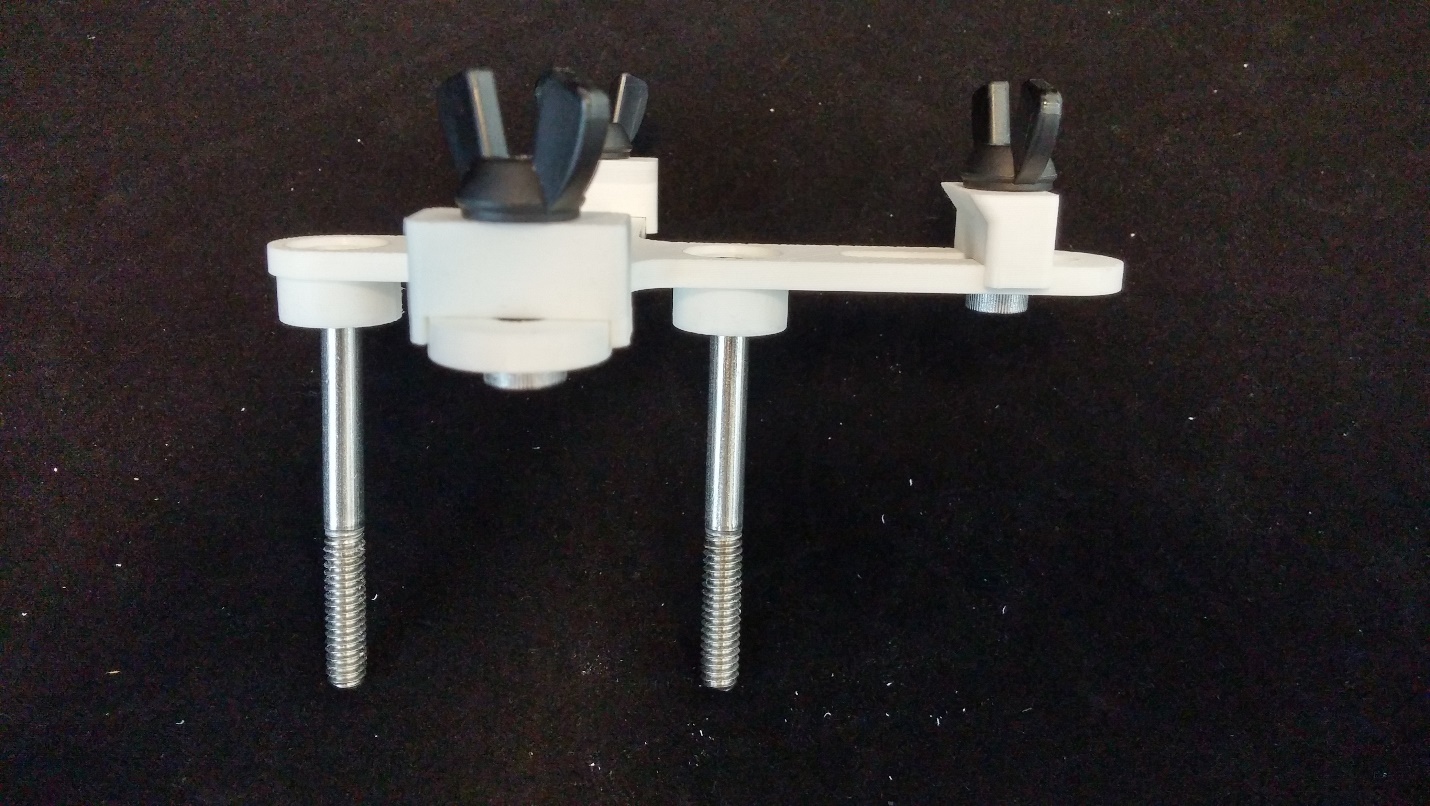


2x

Slide 2 tubes over each screw and place the phone holder on top of the base part. Use this tool to fix the screws.

25


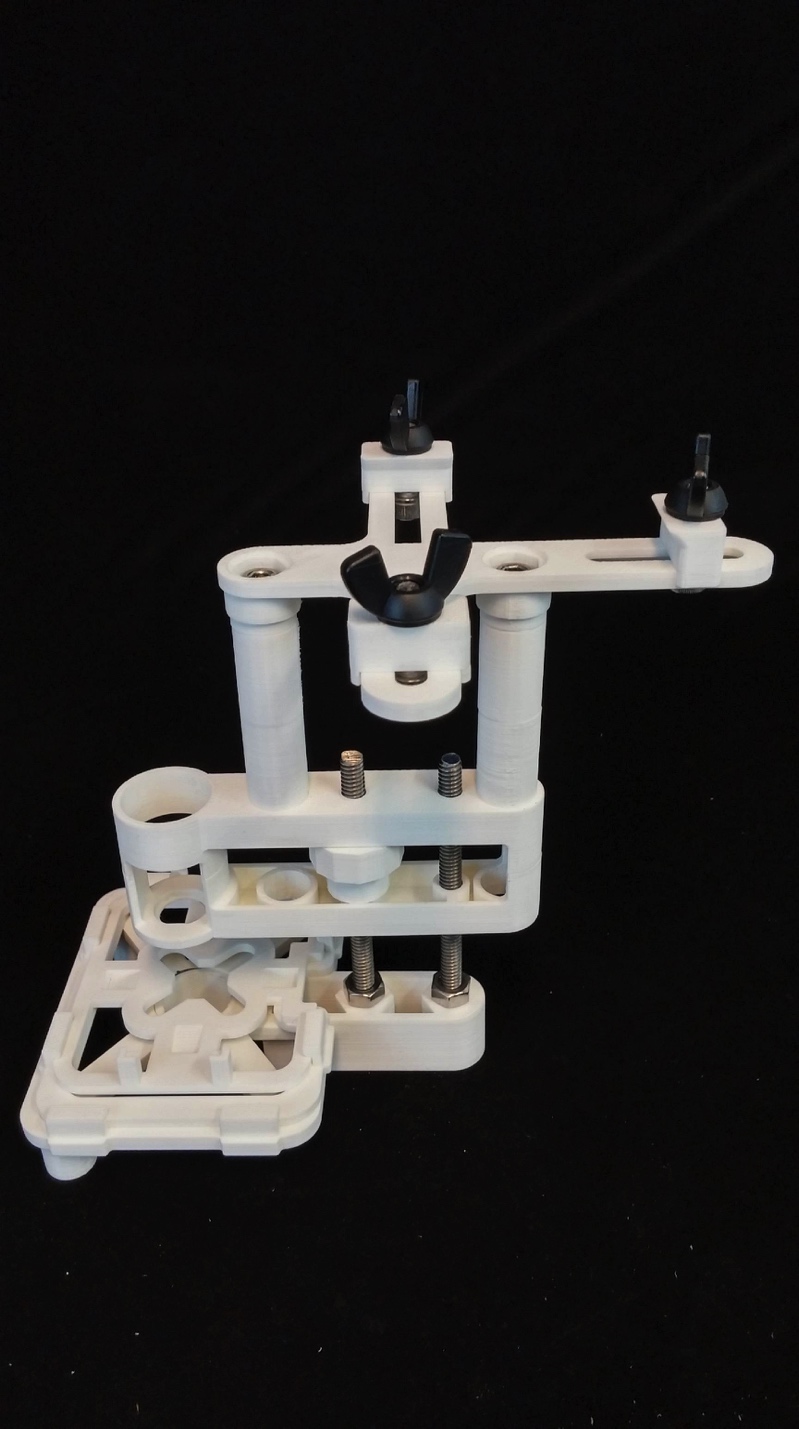


26

1. Optical components:


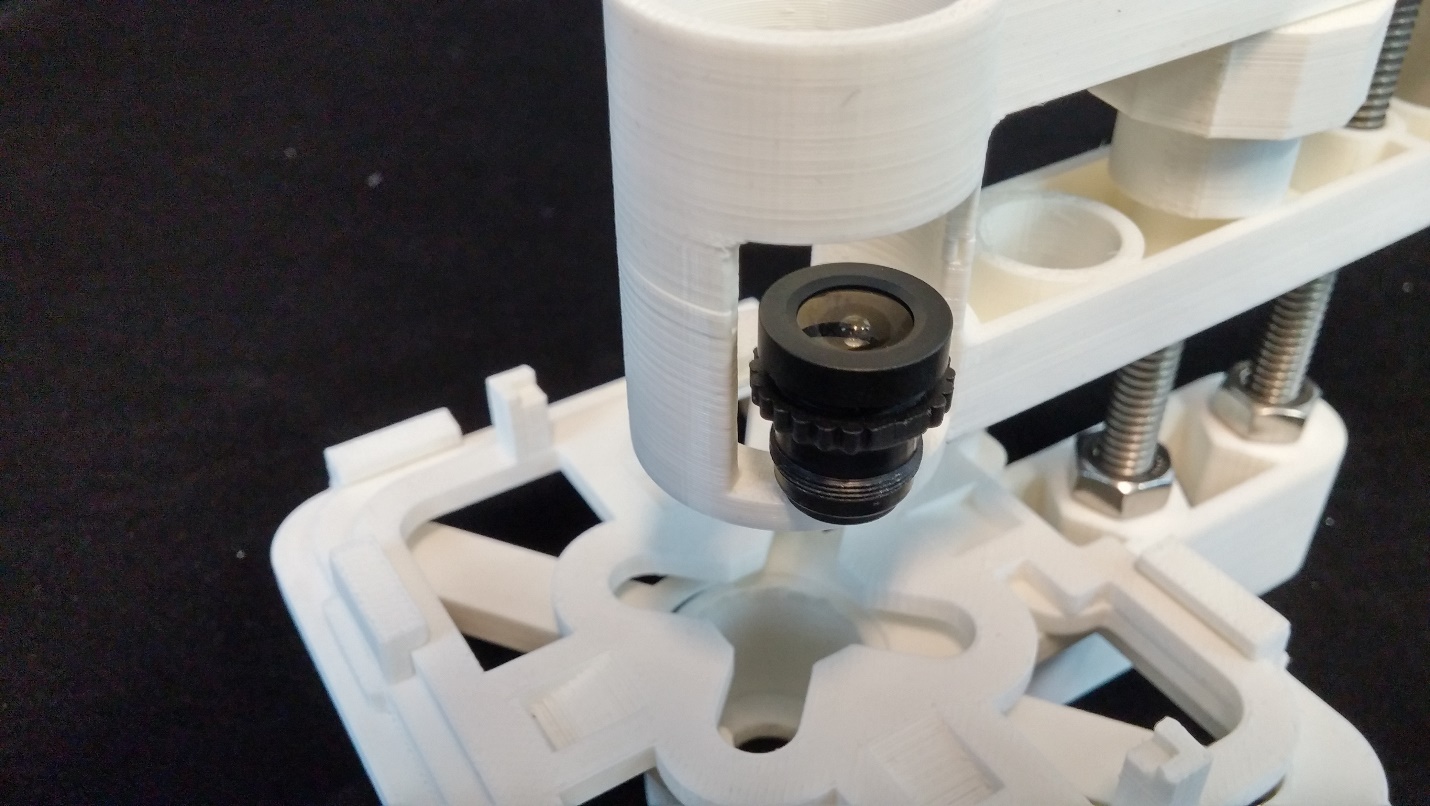


27


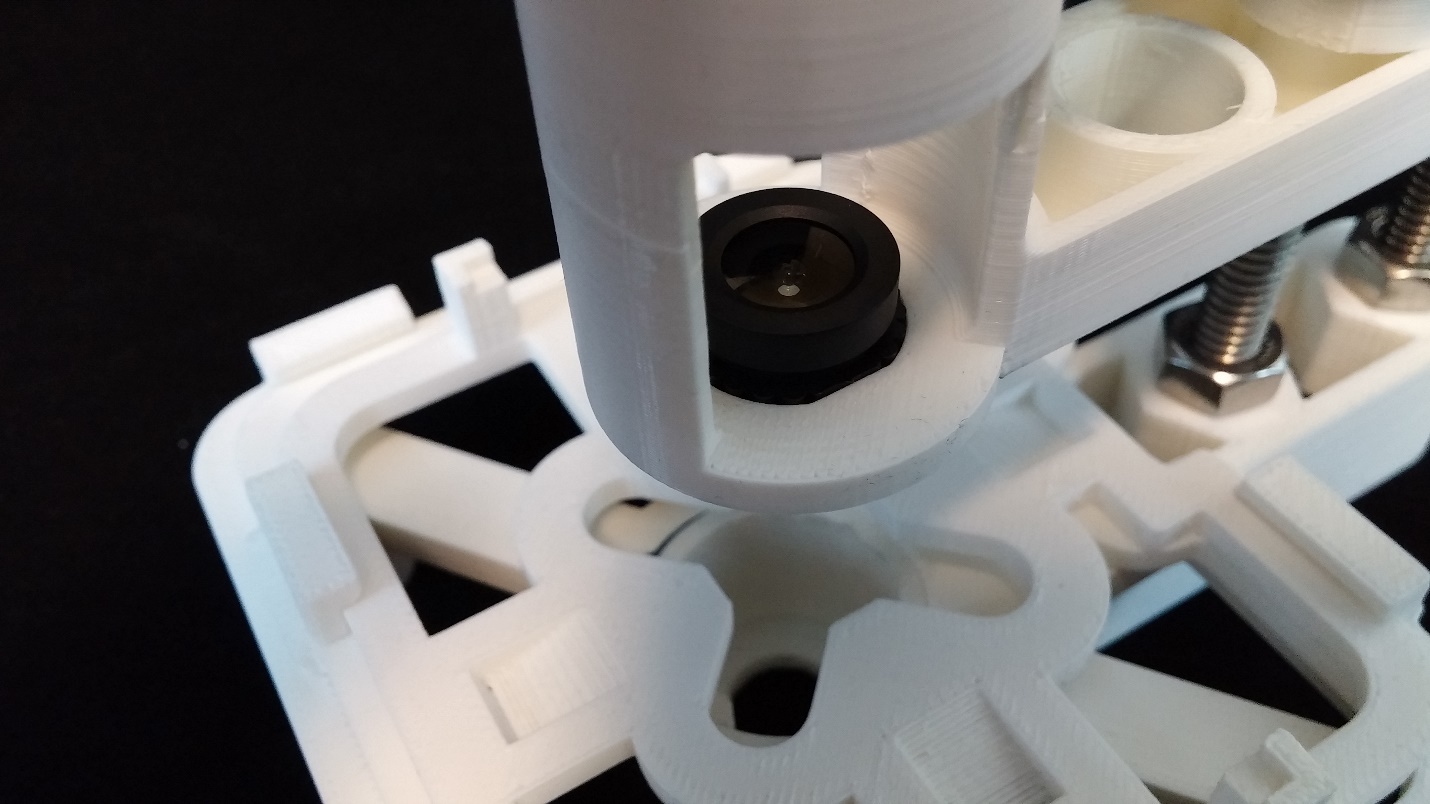


28

Make sure the bottom lens sits tight in the indention.


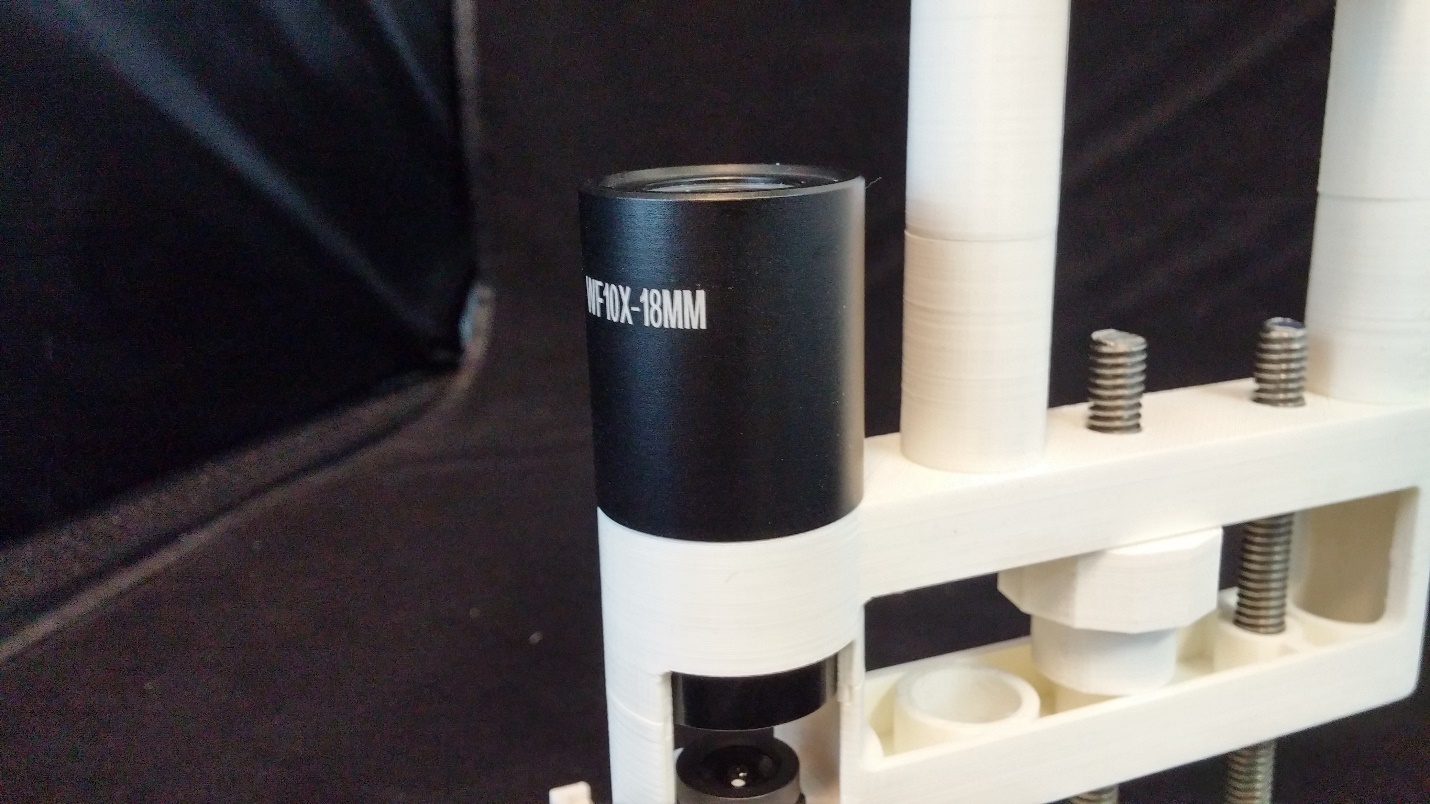


29

Place spacers here if needed to adjust the spacing between the objective and eyepiece

1. Building finished

30


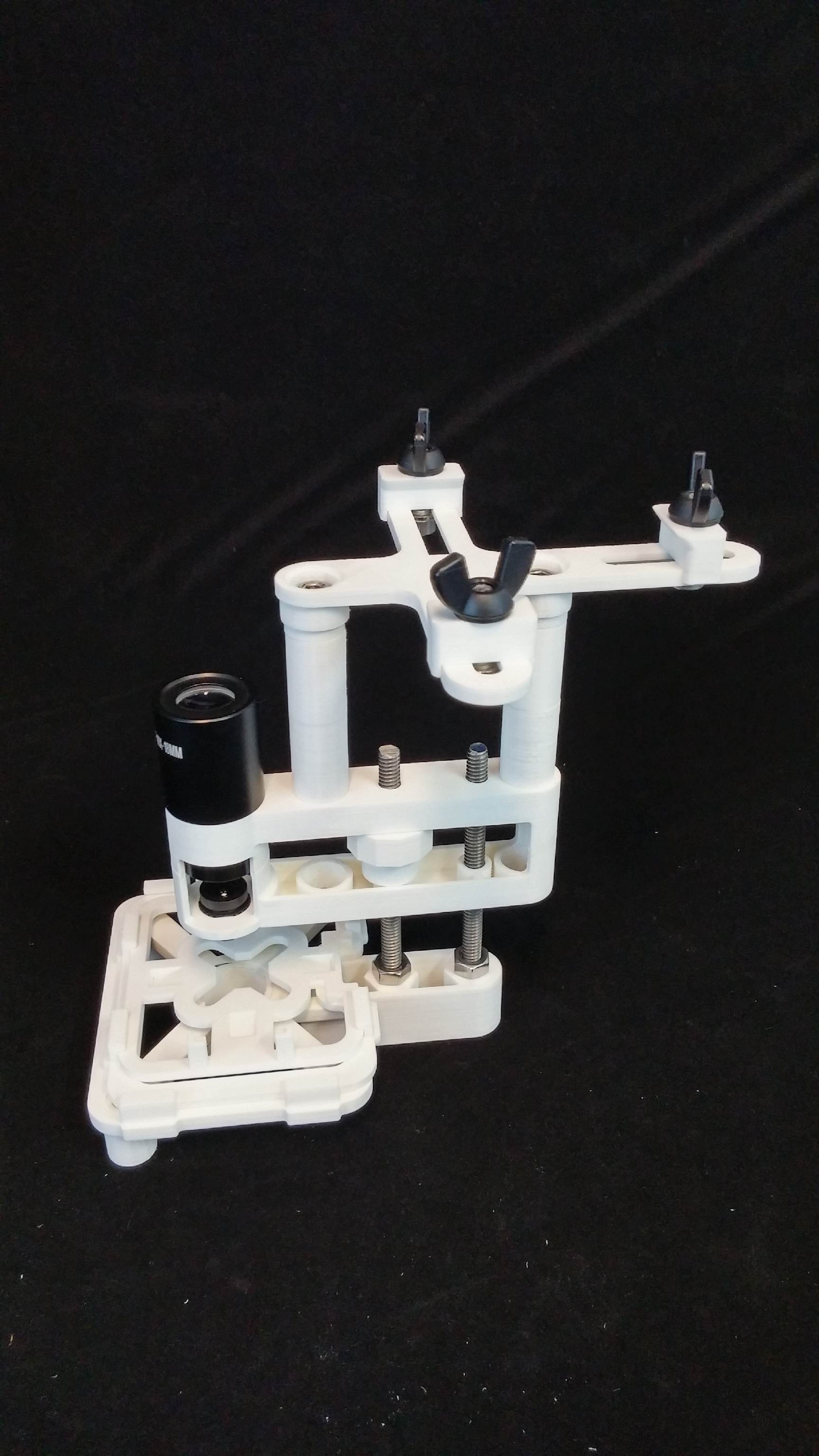


Check again that all screws are tight.

**Building Instructions 2: Circuit**


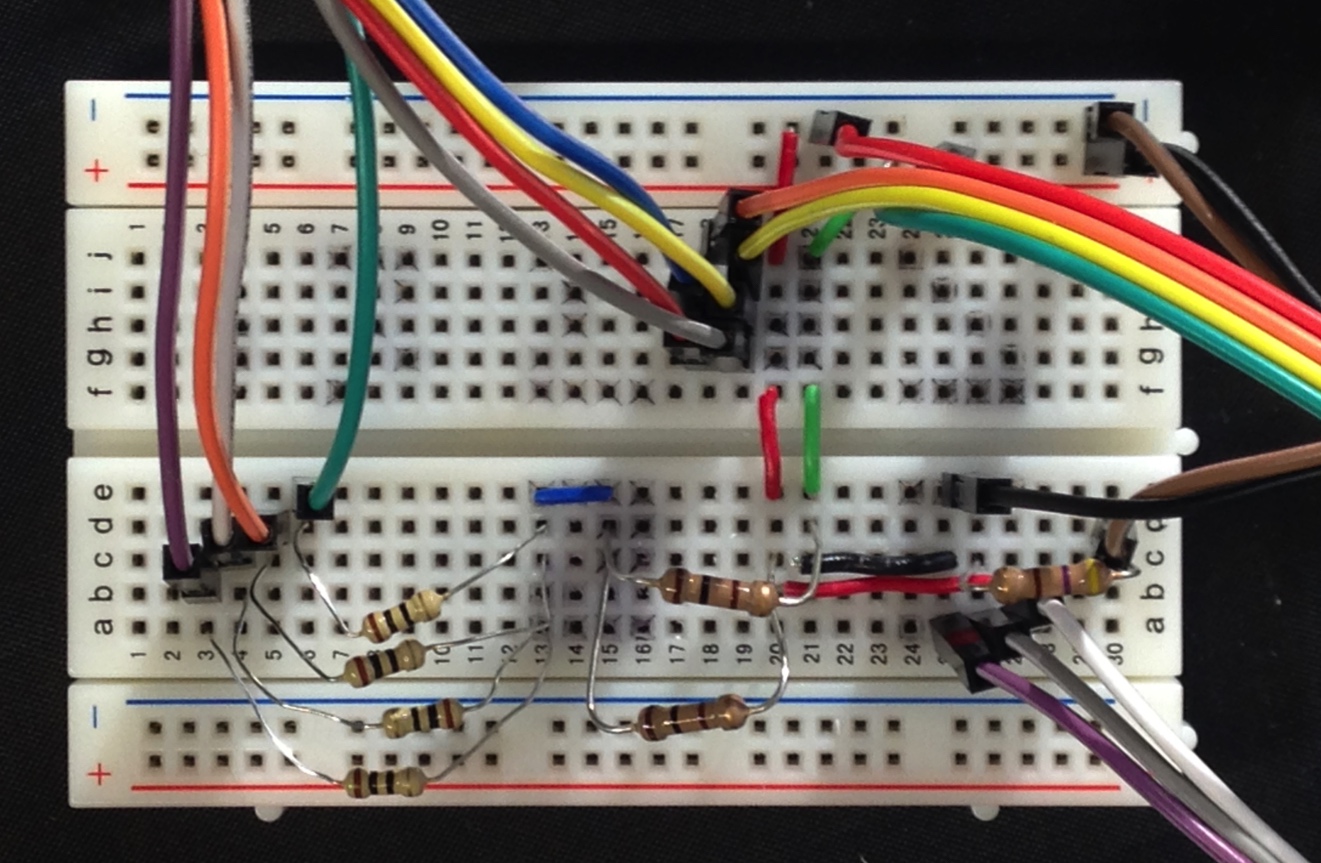


Parts:

| **Label** | **Part** | **#** |
| --- | --- | --- |
| **A** | Illumination LED | 1 |
| **B** | 9v power outlet | 1 |
| **C** | Barrel jack | 1 |
| **D** | Potentiometer (10kohm) | 1 |
| **E** | Directional LEDs | 4 |
| **F** | Joystick | 1 |
| **G** | 500 0hm 1/4 Watt resistor (Yellow, Purple, Brown) | 1 |
| **H** | 100 0hm 1/2 Watt resistor ­­­­(Brown, Black, Brown) | 2 |
| **I** | 10 ohm 1/4 Watt resistor (Brown, Black, Black) | 4 |
| **J** | Connectors | 7 |
| **K** | Breadboard | 1 |


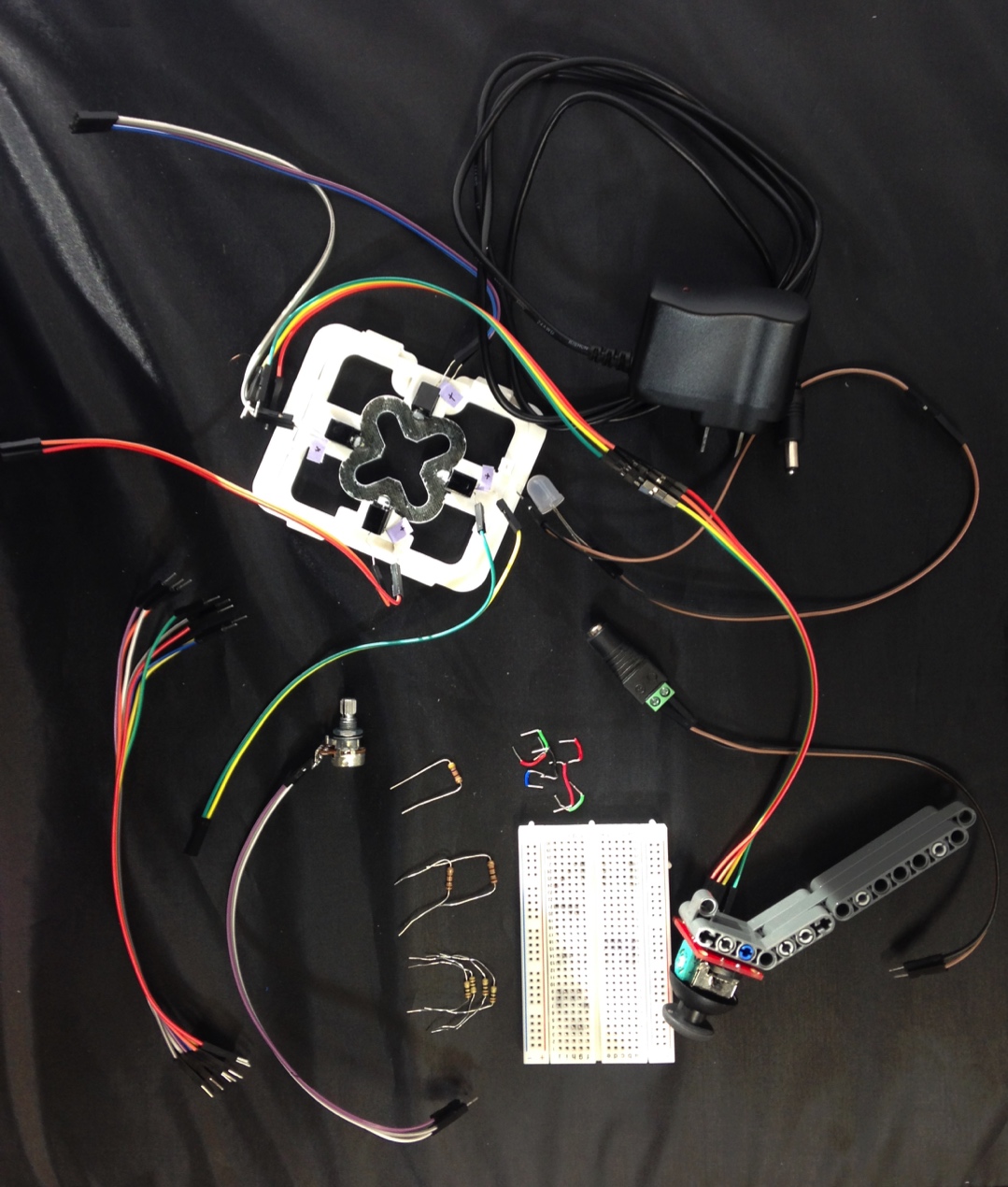
­

**K**

**F**

**J**

**I**

**H**

**G**

**D**

**C**

**E**

**A**

**B**

**LED extension cables**

**Step 1:**

Assemble the base illumination. The longer leg of the LED is the positive leg. Be careful to choose the correct resistor – match the band colors!

After this step, you should be able to see the illumination LED turn on: Plug the power cable into the base and wall outlet. Try twisting to potentiometer to adjust the brightness. Try swapping wires on the LED if there is no light.

**Pay attention to colors and orientation.**


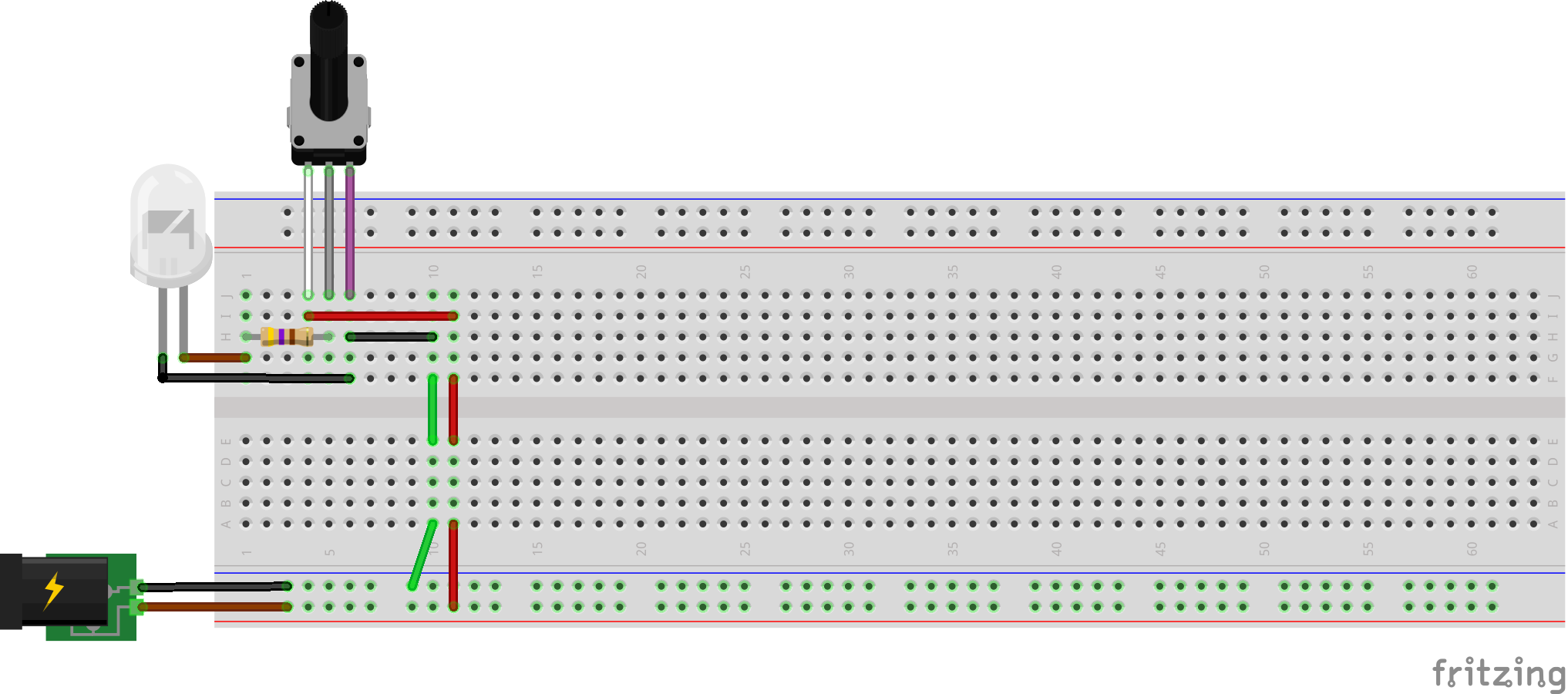


**Step 2: Assemble single directional LED**

After this step, the base illumination should still be on. Furthermore, the directional LED should turn on when the joystick is pushed in the corresponding direction. If directional LED does not turn on, first try swapping positive and negative on the LED legs before trouble shooting rest of circuit.


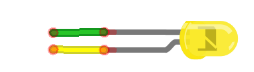

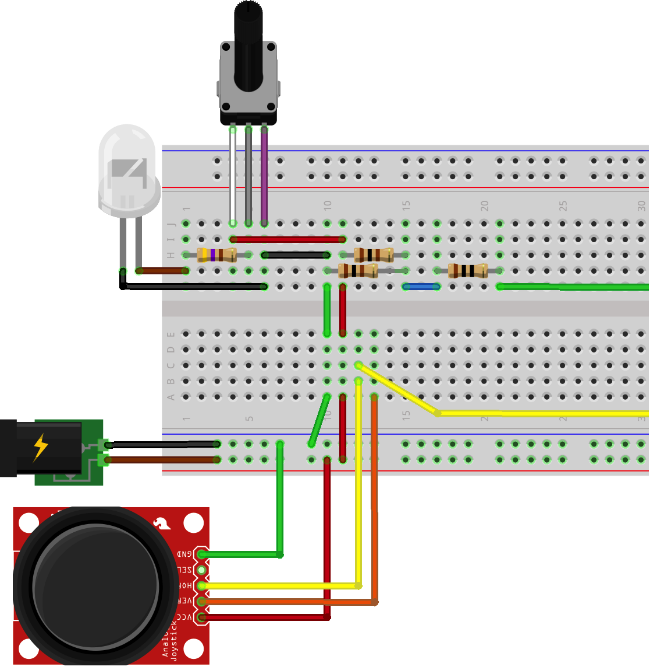


**Pay attention to the orientation (+/-).**

**Pay attention to colors.**

**Long**

**Short**

**Step 3: Assemble all LEDs**

After this step, you have a fully functional circuit! Be careful that exposed resistor legs do not touch one another. Test if all 4 LEDs light up when the joystick is pushed to the specific side.


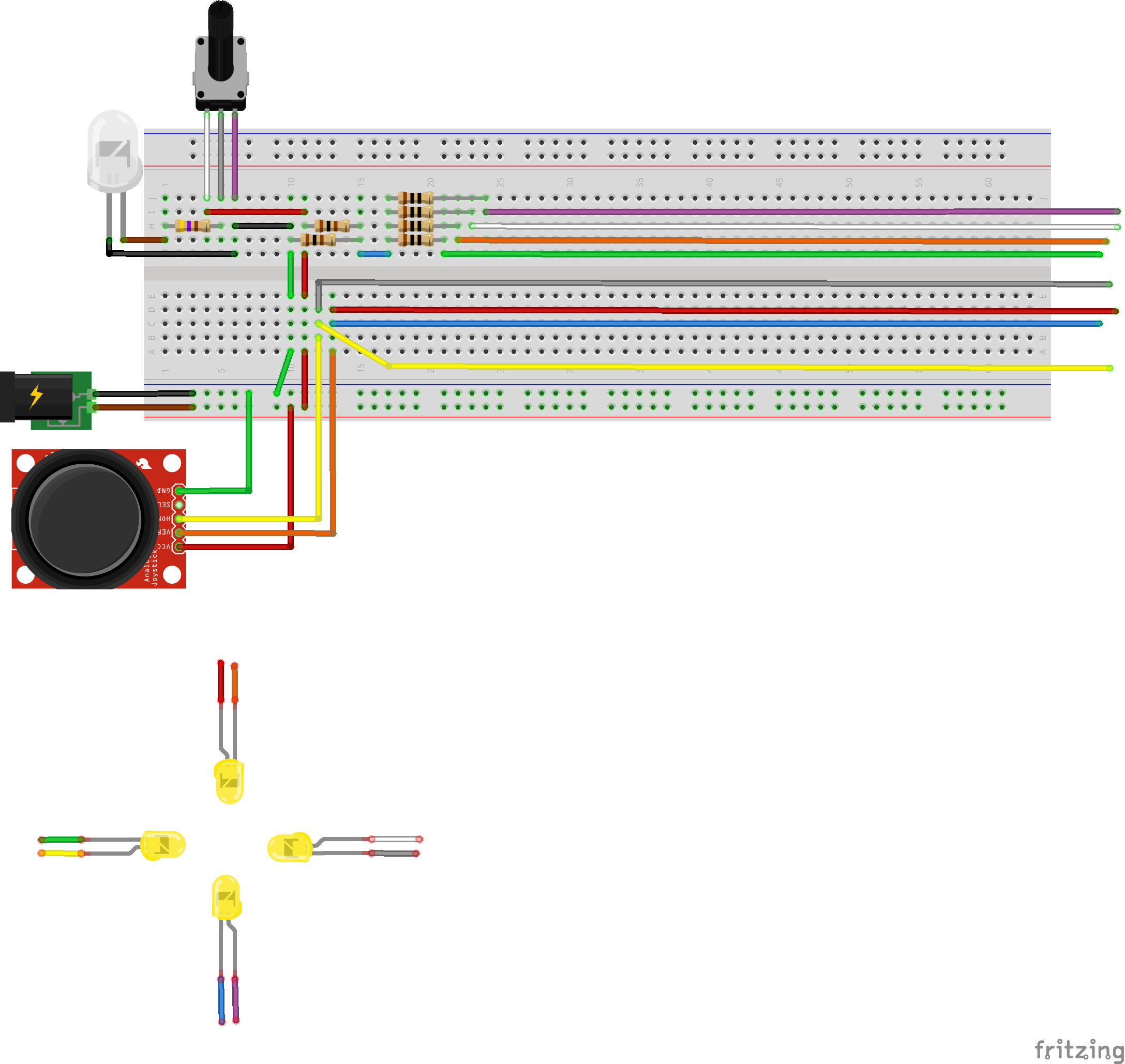

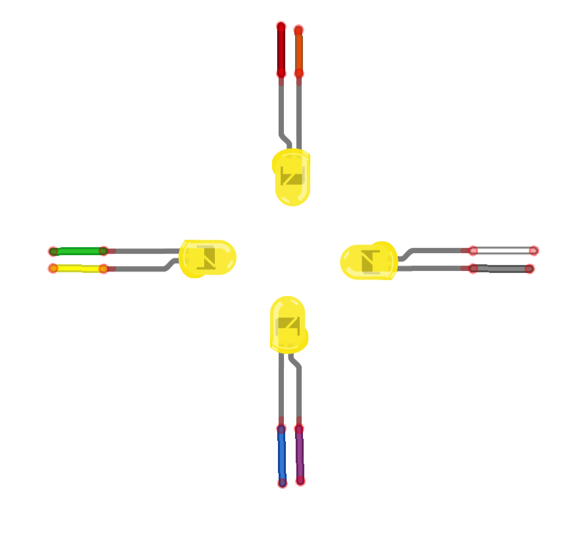


**Pay attention to the orientation (+/-).**

**Short**

**Long**

**Short**

**Long**

**Short**

**Short**

**Long**

**Long**

**Step 4: Final circuit**


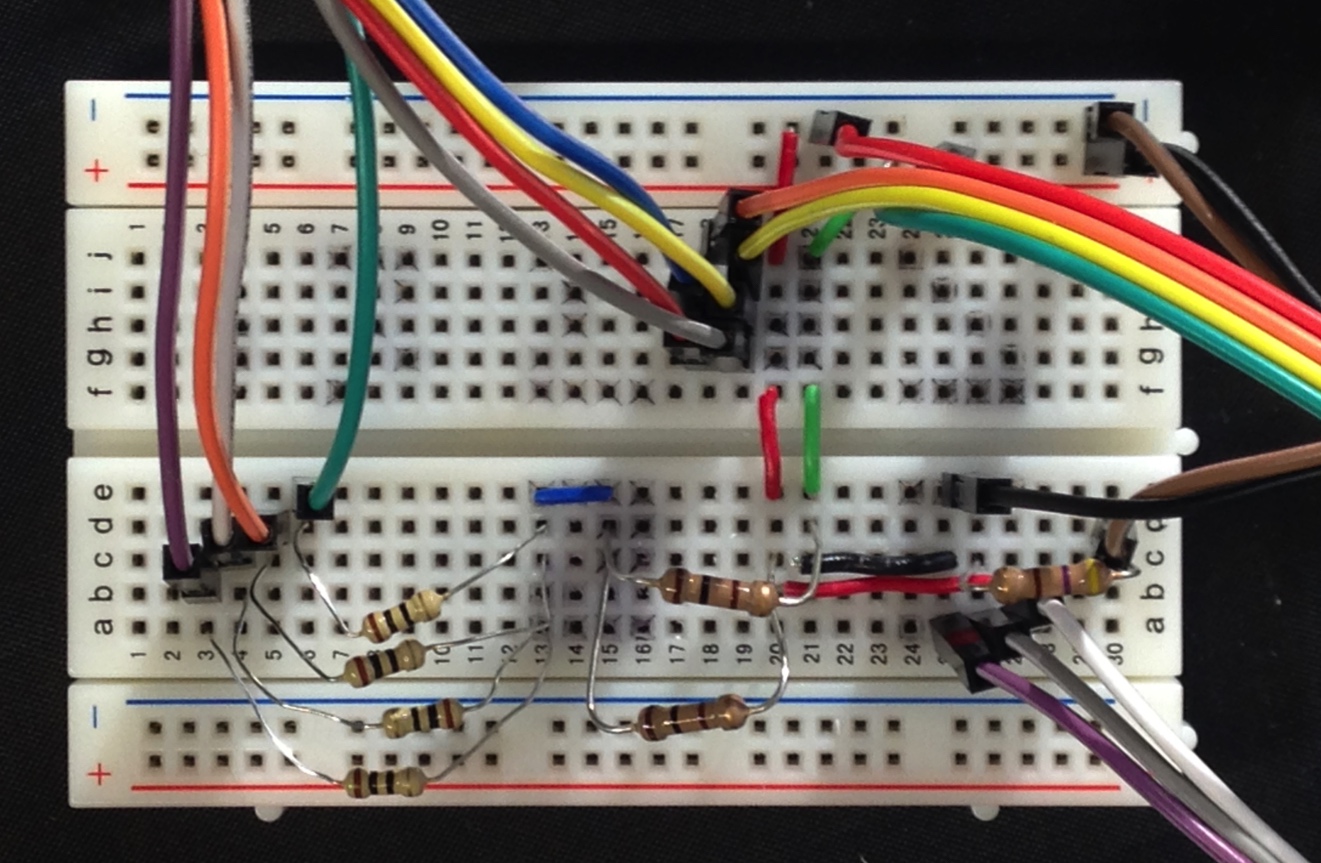


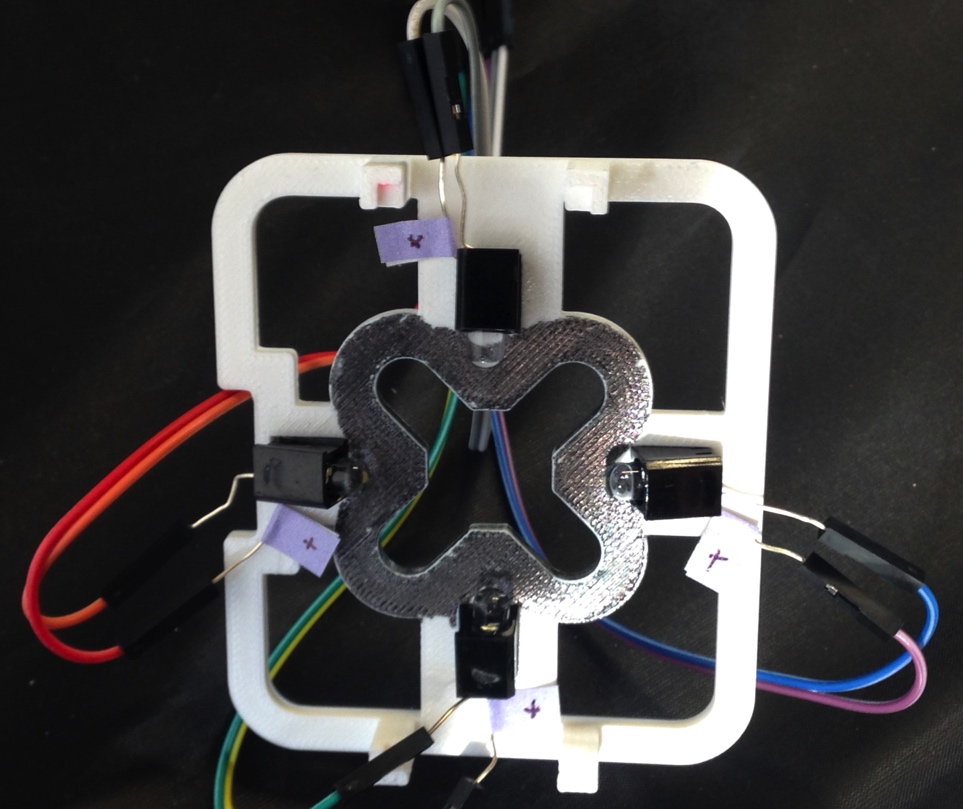


**Building Instructions 3: Mini-aquarium**


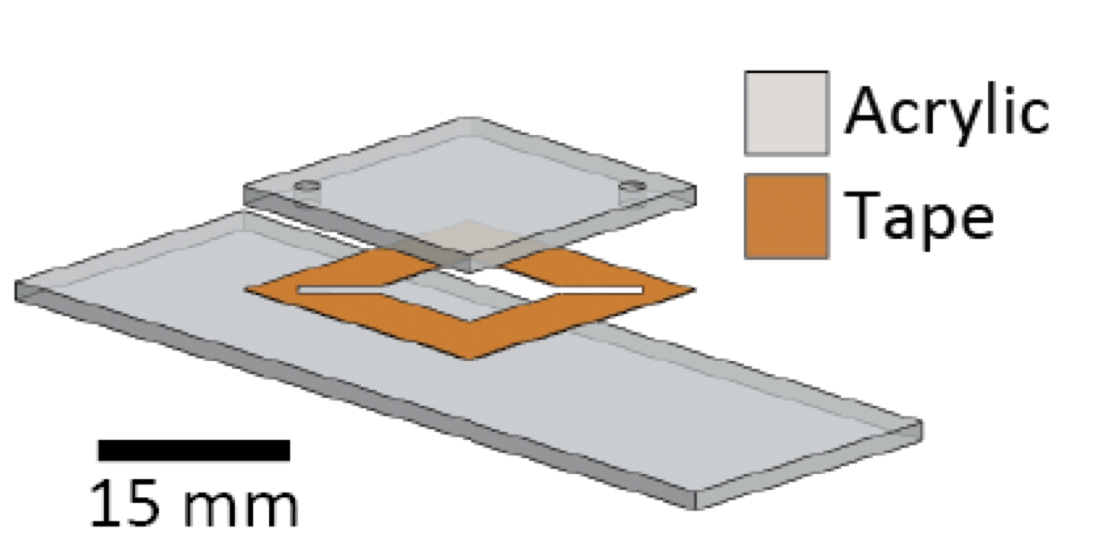
Parts:

|  | **Part** | **#** |
| --- | --- | --- |
| **A** | Base acrylic slide | 1 |
| **B** | Double-sided tape | 1 |
| **C** | Top slide with in- and outlet | 1 |
| **D** | Pipette | 1 |
| **E** | Euglena cells in vial | 1 |


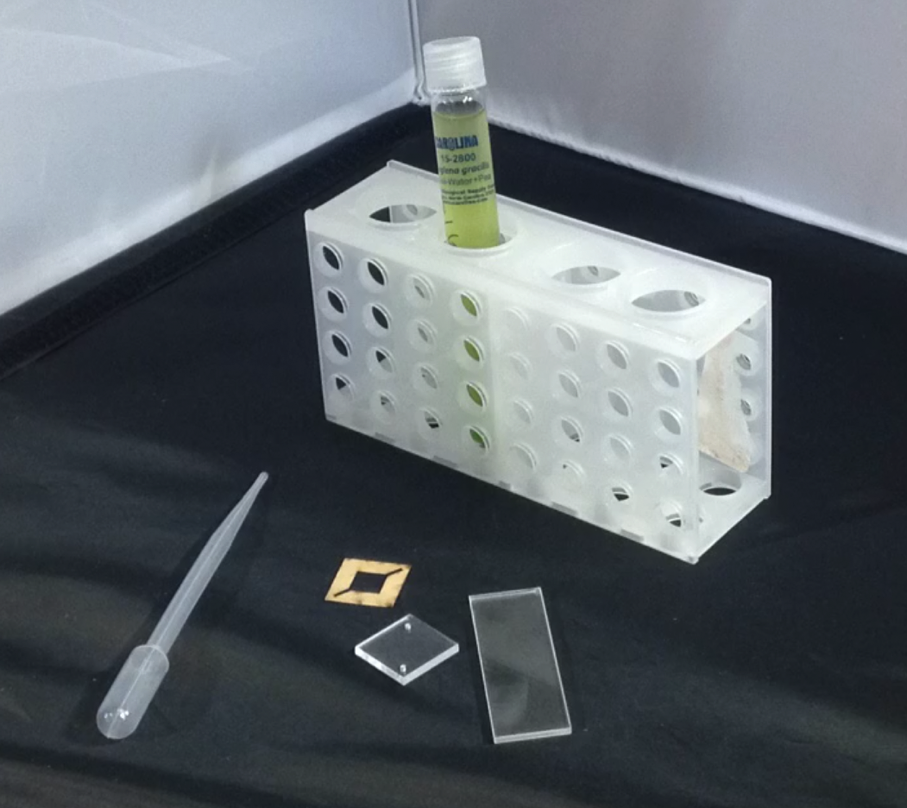


**A
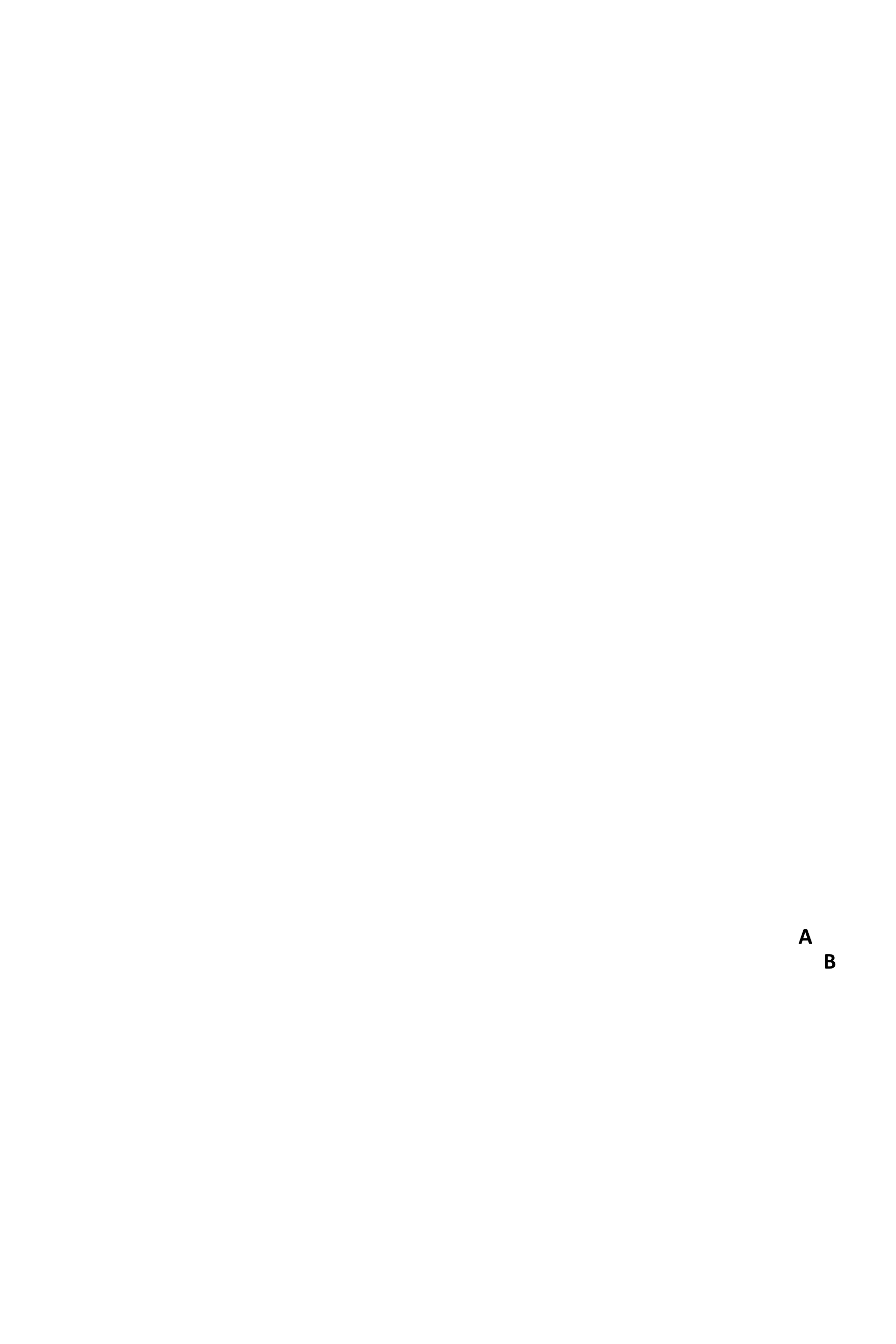
**

**B
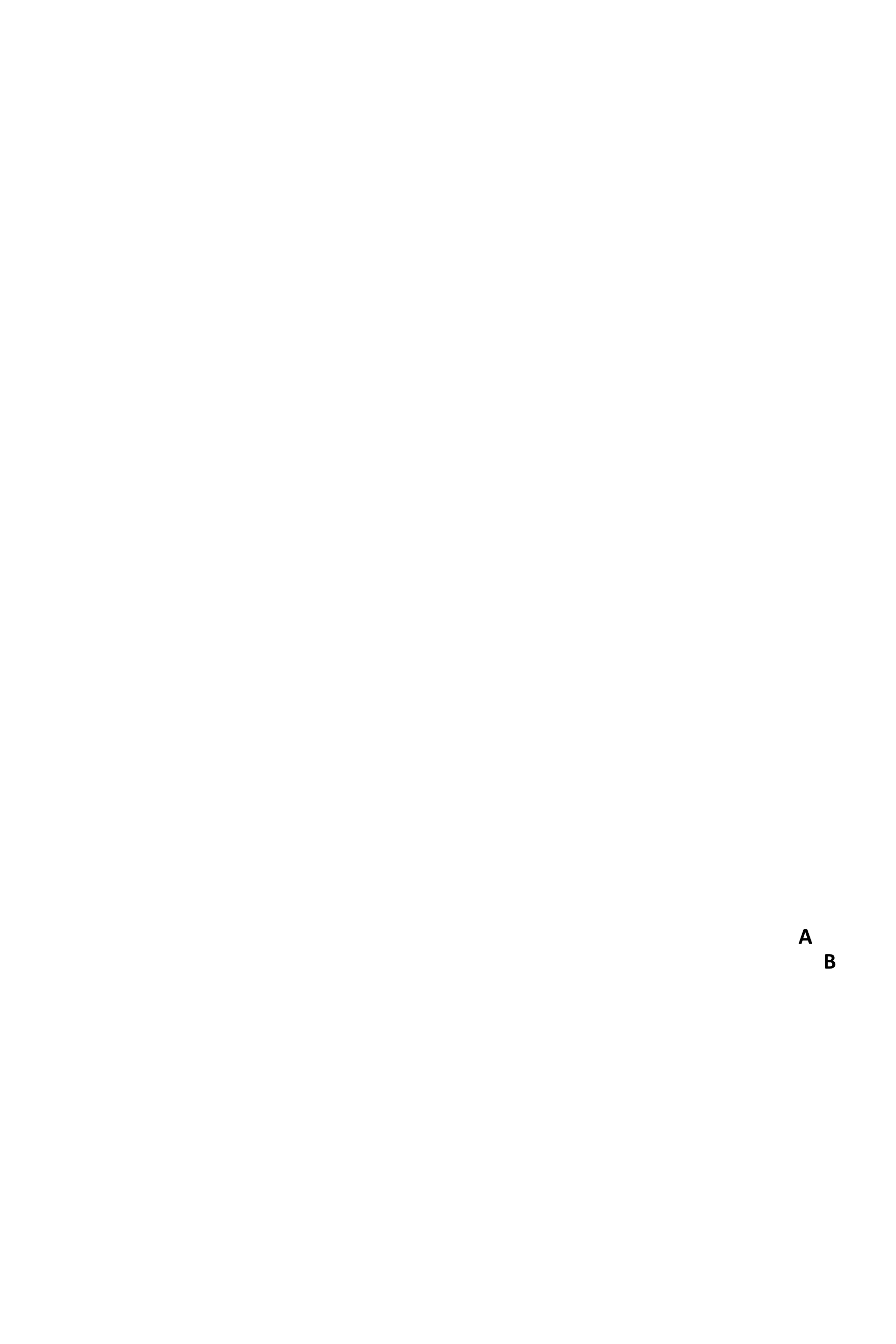
**

**E
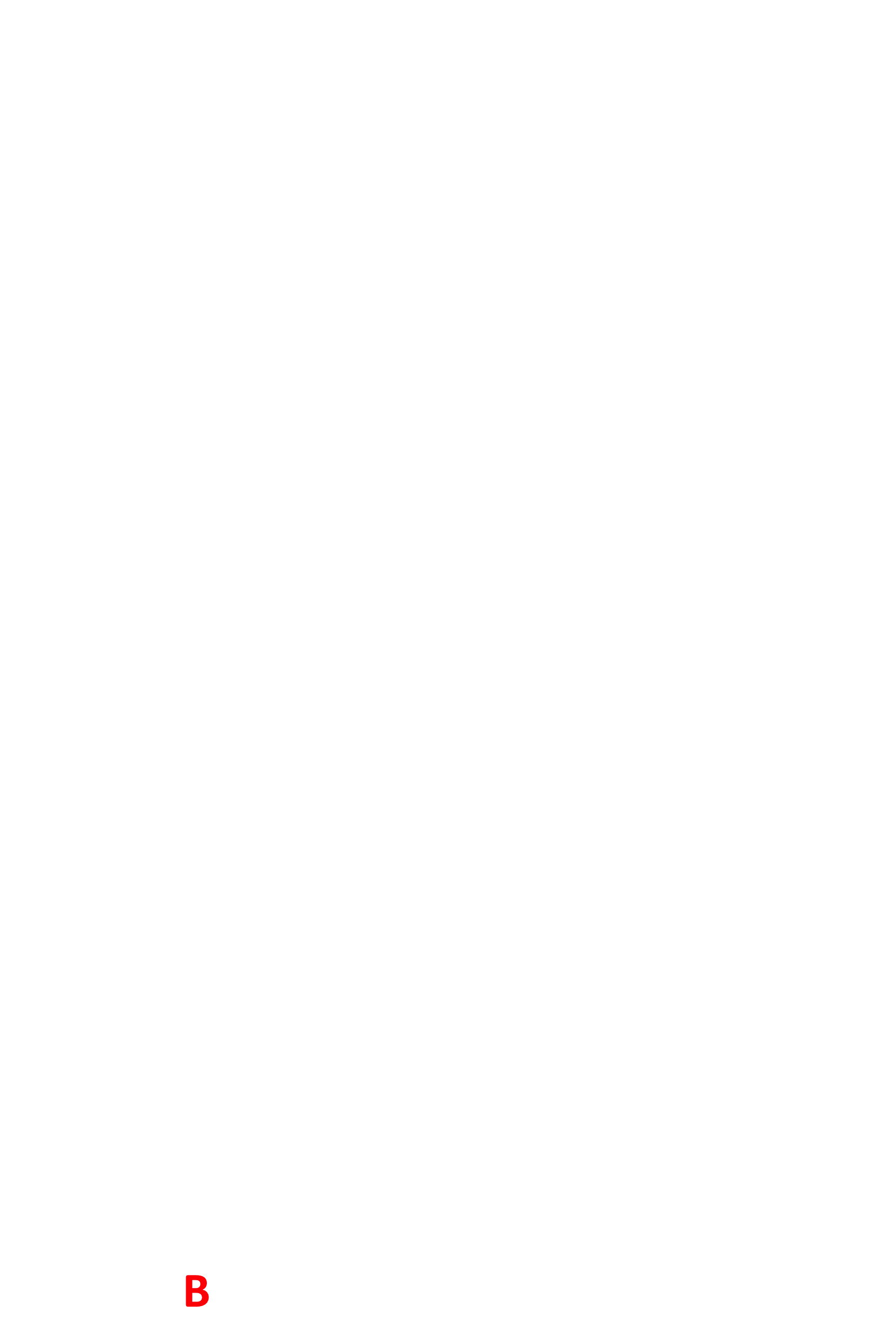

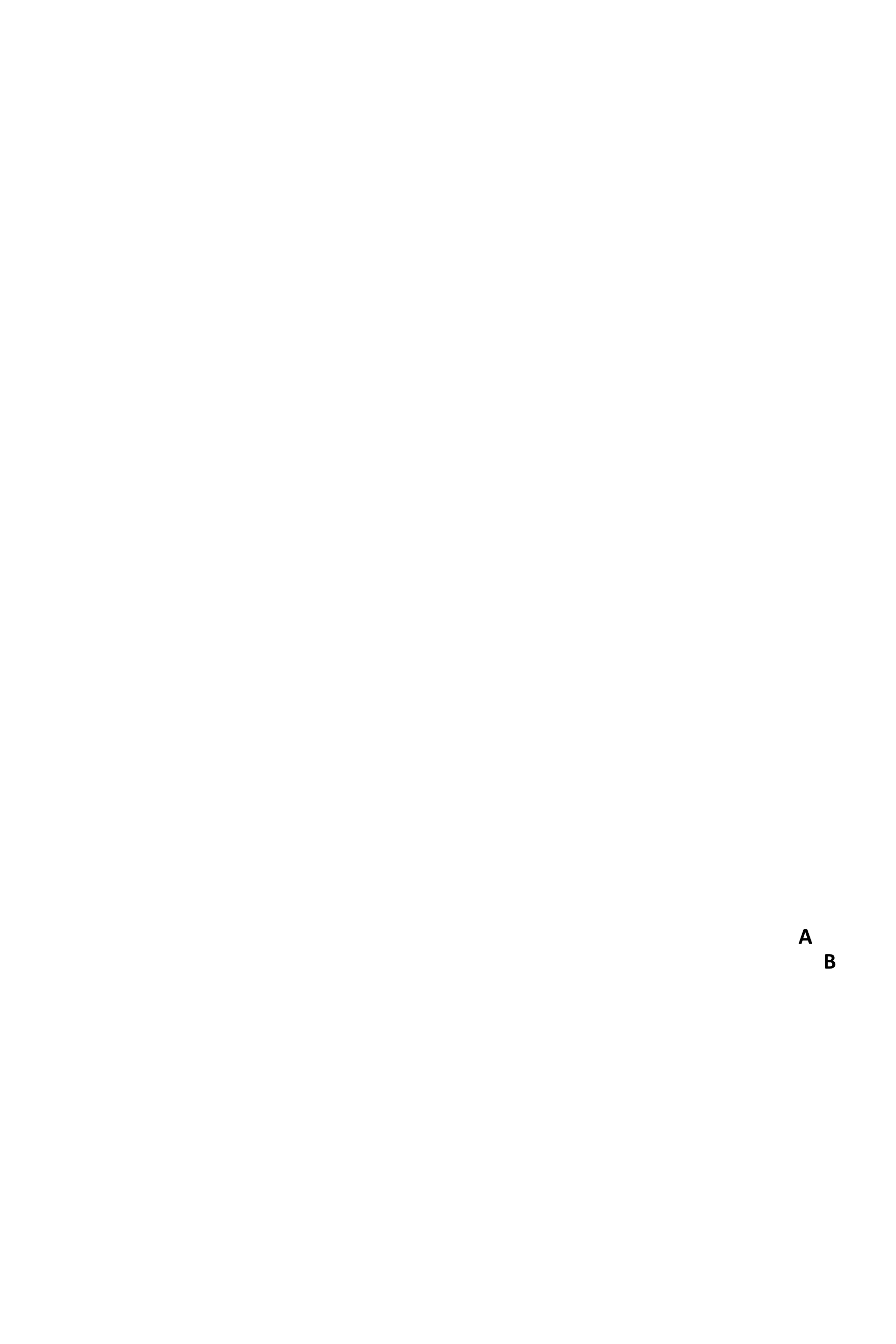
**

**D
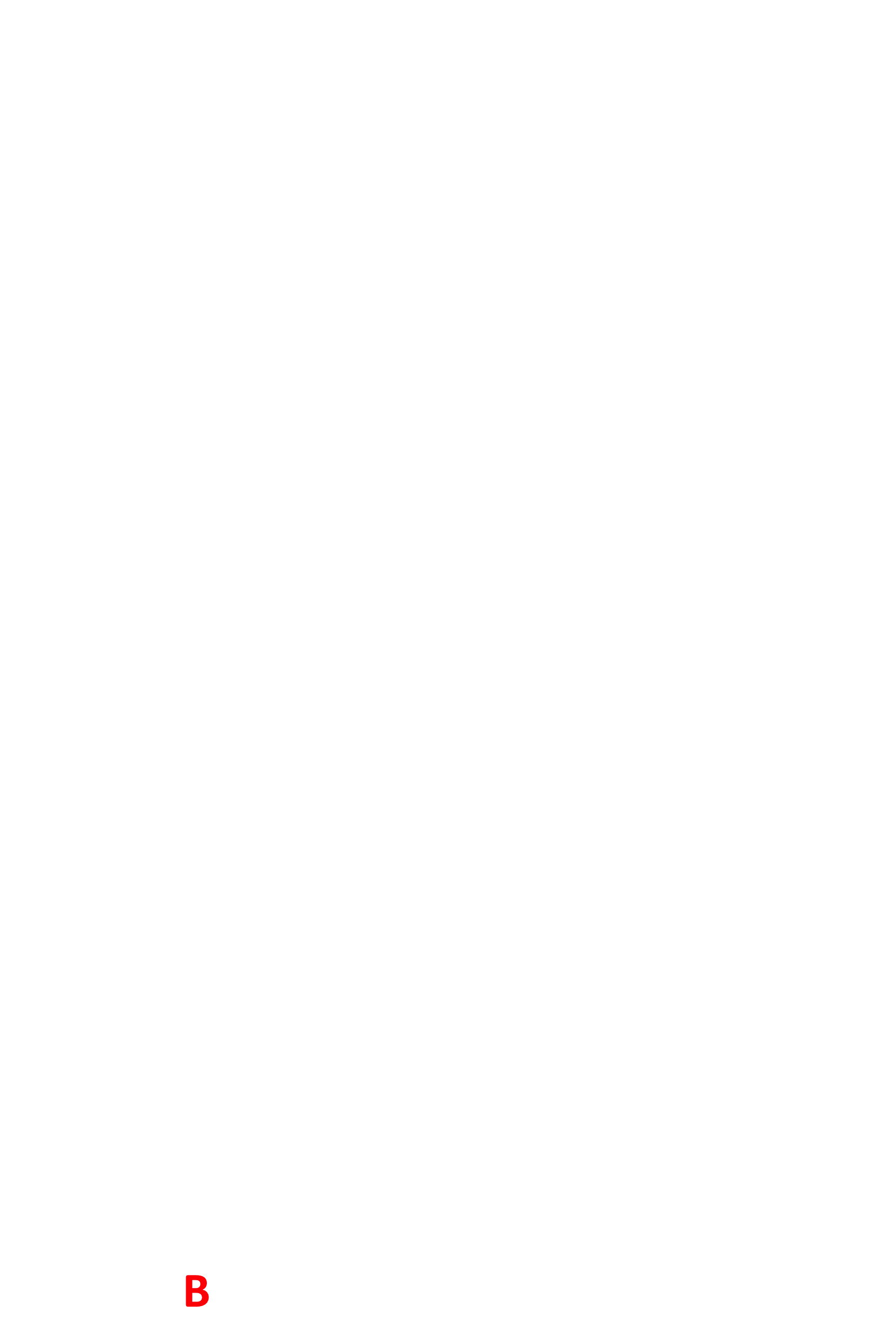

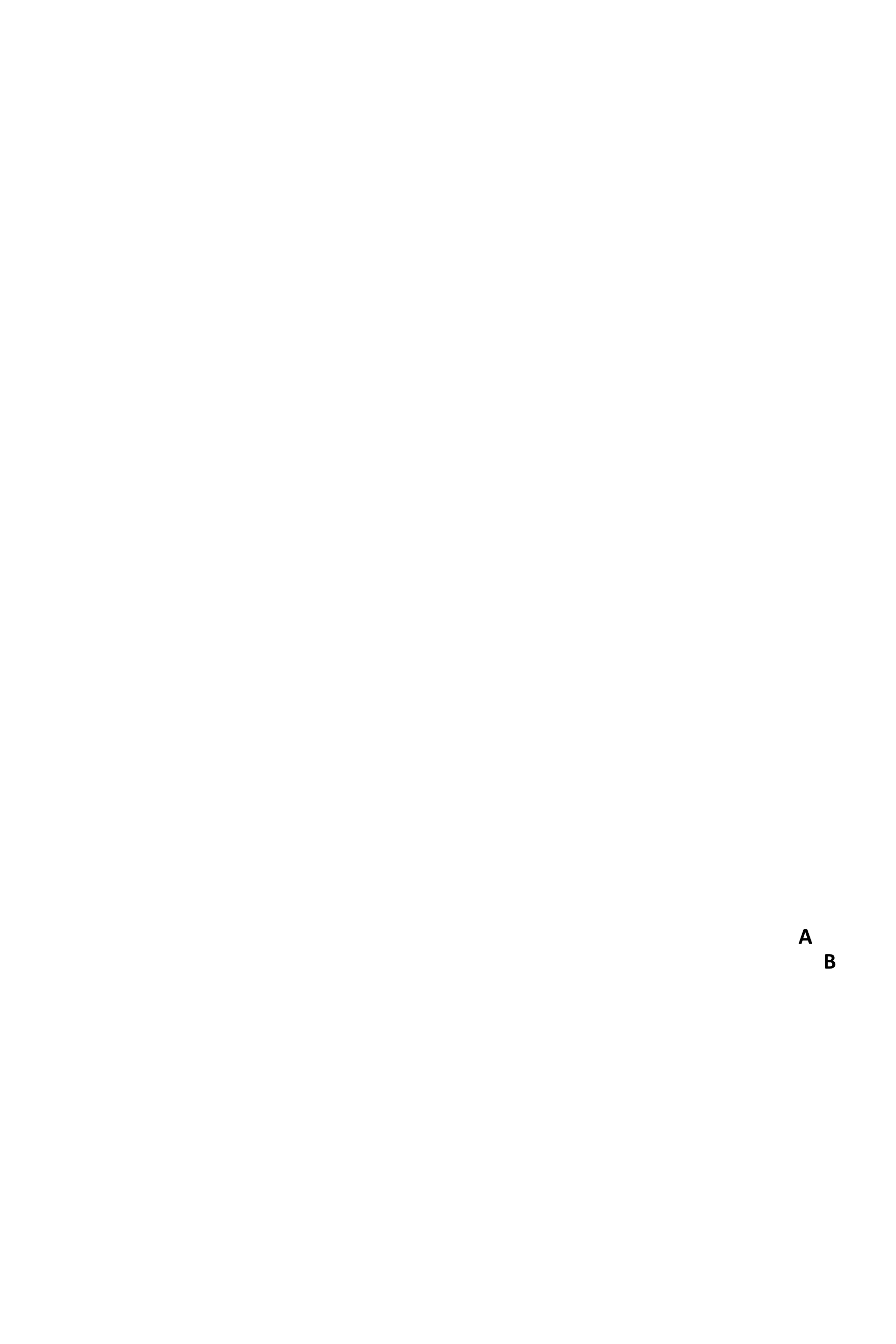
**

**C
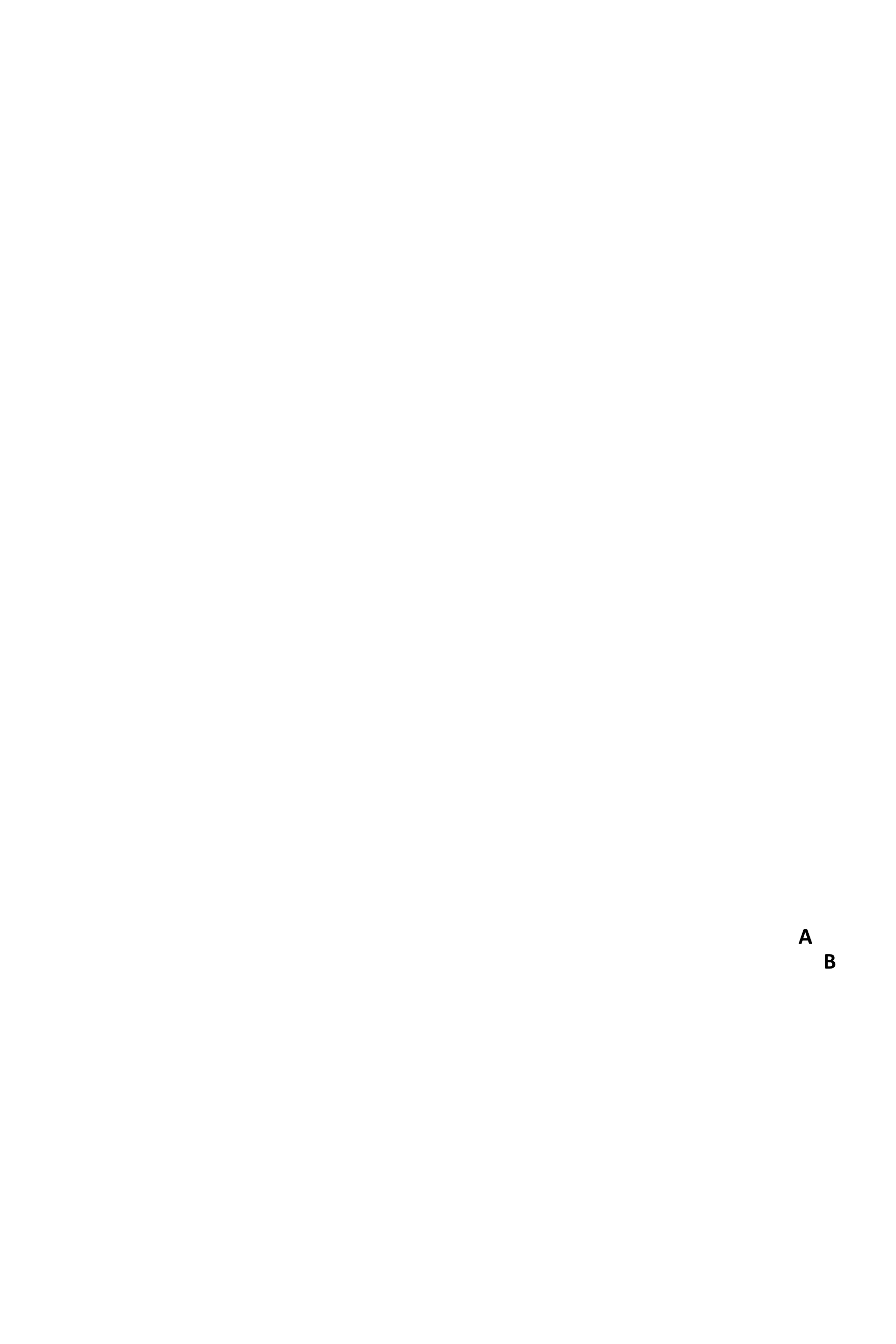
**

**Step 1:**

Peel off one protective paper from the double-sided tape. Stick it to the top layer while aligning the holes. Press firmly to make good contact.


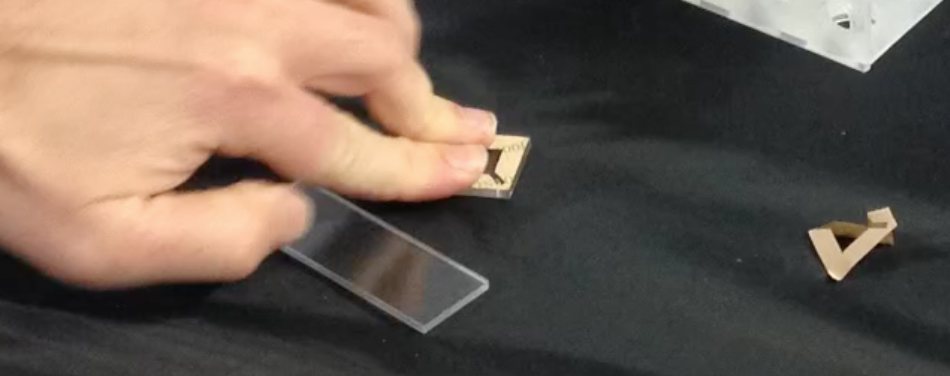


**Step 2:**

Peel off second protective paper from the double-sided tape. Stick it to the center of the base layer. Press firmly to make good contact.


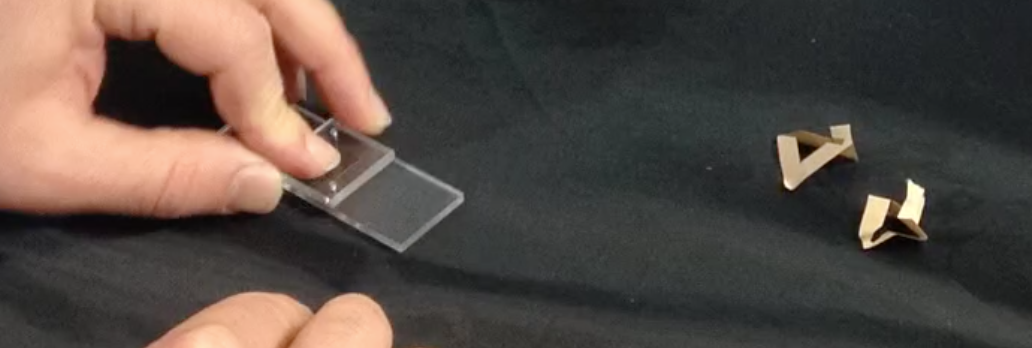


**Step 3:**

Open Euglena container and use pipette to suck up some of the Euglena. Place a small drop onto one hole. Observe how the fluid flows into the chamber. Excess liquid can be removed with the pipette or swiped off.

Now you are ready to put it under the microscope 🡪 See Building Instructions 4: Final assembly and focusing.
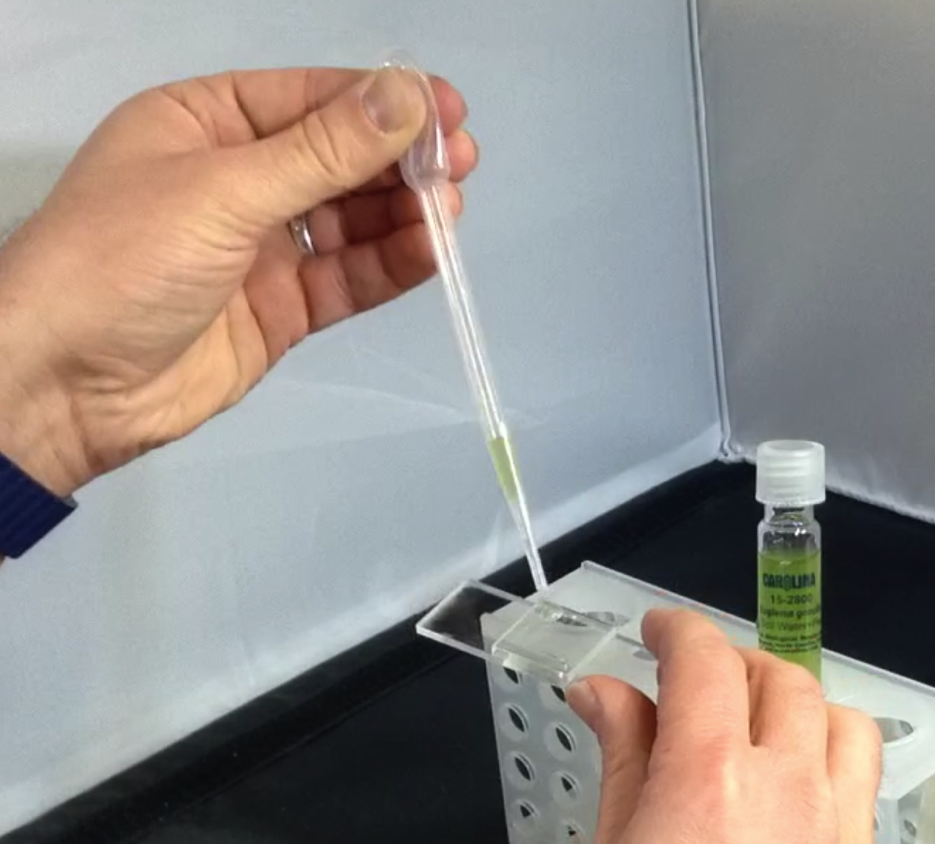


**Step 4: Optional**

For long term operation tubes can be attached to the holes. Like this the mini-aquarium can be flushed from a reservoir with fresh Euglena.


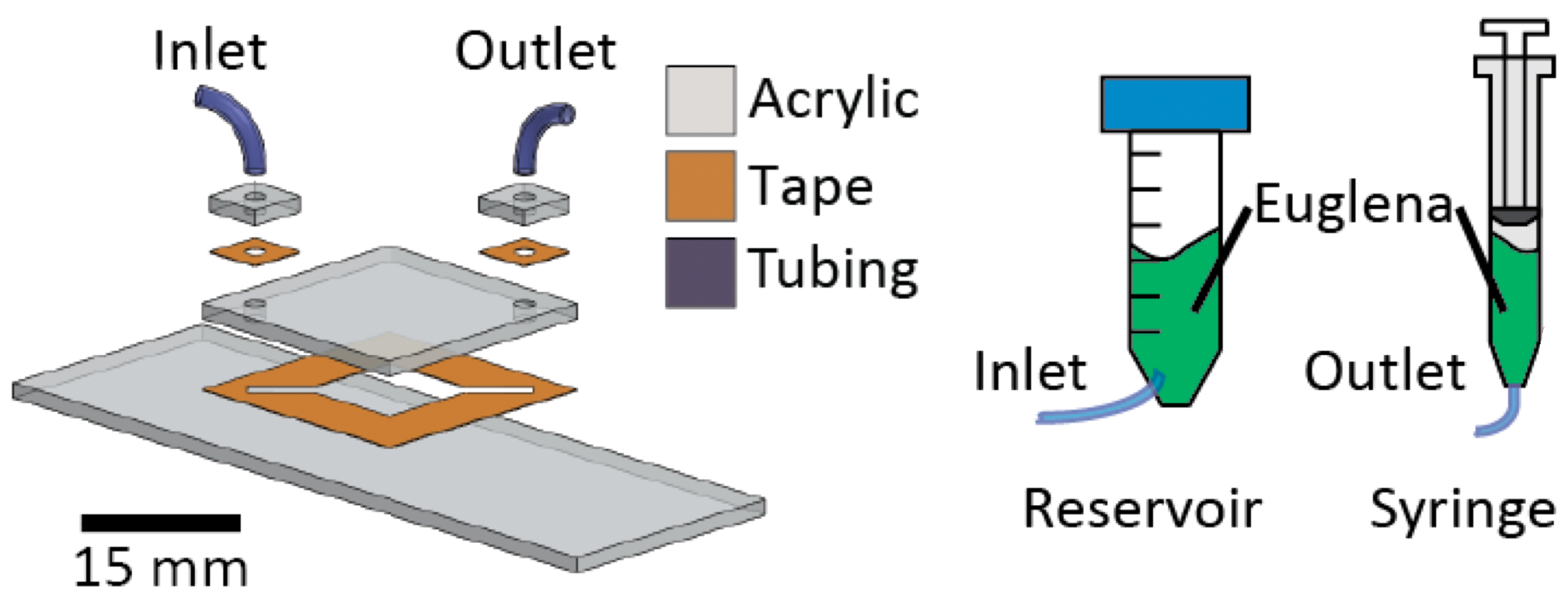


**Building Instructions 4: Final Assembly and Focusing**

1.
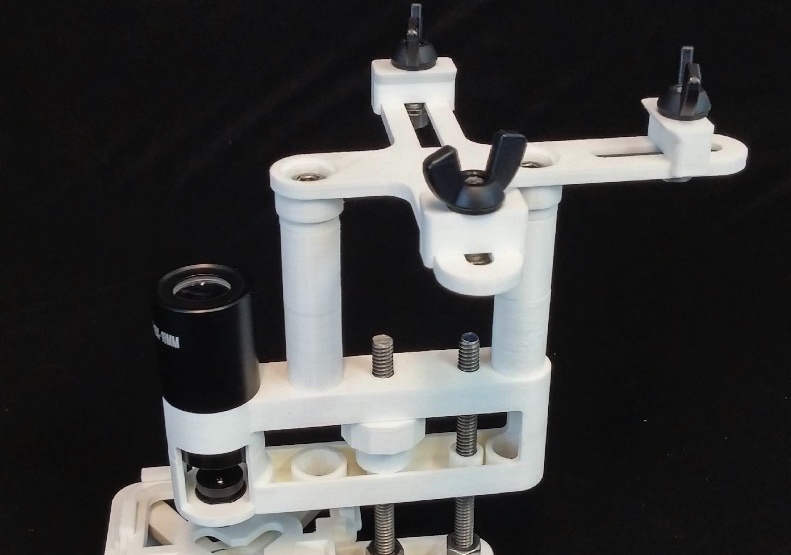
Loosen the wingnuts on the holder.

Rough focus knob

Fine focus knob

Wingnuts

1. Place your phone on the holder.
2. Turn on the illumination LED to highest brightness by turning the knob in the circuit.
3. Open the standard camera application.
4. Move your phone around until the bright white circle is centered. It appears like a bright flash when you are at the right spot.


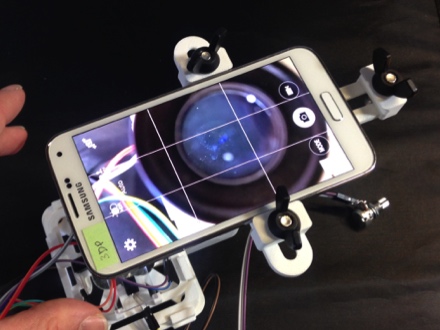

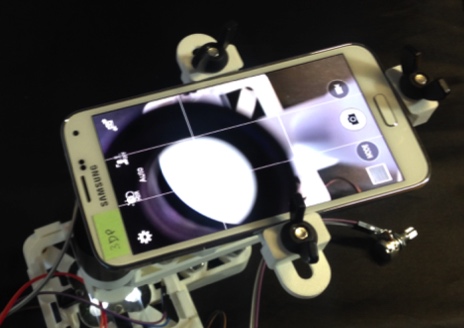

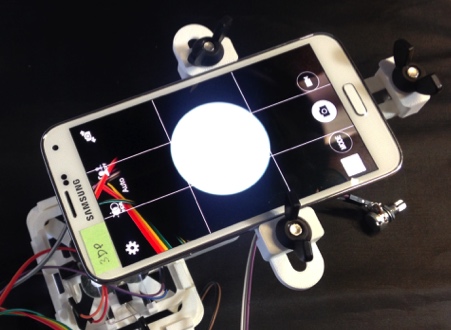


Not aligned. Almost... Perfect!

1. Close the sliders and tighten, being careful not to move the phone.
2. Place a slide with a clear pattern in the sample holder. The red side should face down!
   Here we use a red cross in a box.


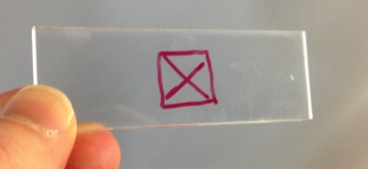


1. Still in the camera app, align your pattern (here a red cross) and move the sample holder until you see some red color. Most likely you don’t see the lines as the microscope is still not focused. Try to focus as best as you can. Then take out this slide.


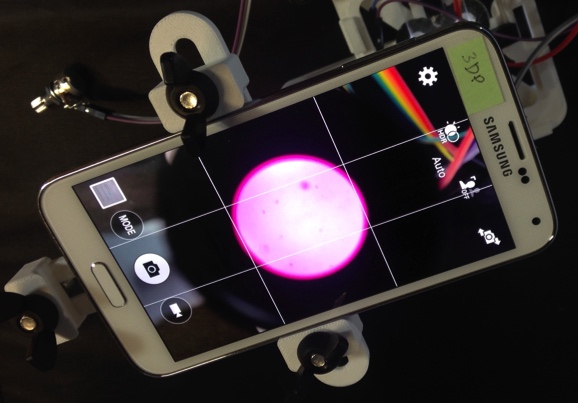


1. Insert the Euglena mini-aquarium with the flat side facing up.


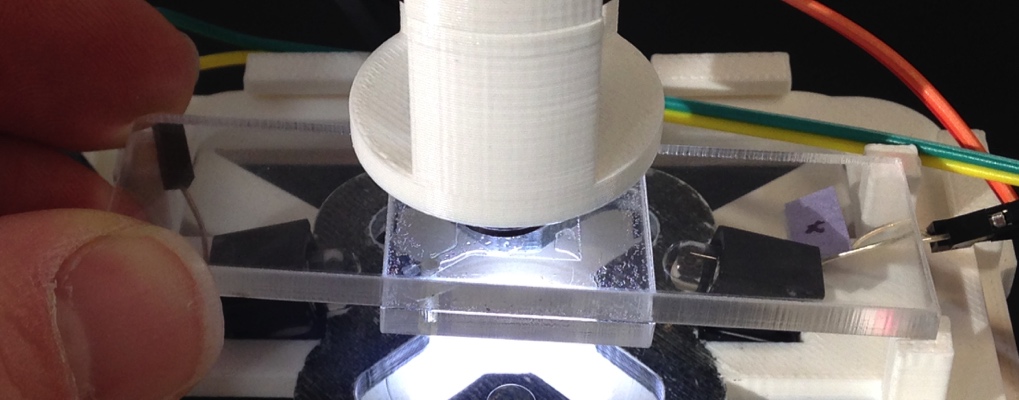


1. Most likely you need to refocus. Try moving just a little down to get Euglena in focus.
2. In case you don’t see Euglena, turn the large focus knob until the objective nearly touches the sample. Slowly back away while watching the screen.
3. Picture 31 shows approximately the position and how much of the screws should be sticking out.
4. When the sample comes into focus, use the fine focus knob to fine tune.
5. Switch to the LudusScope app and start playing.


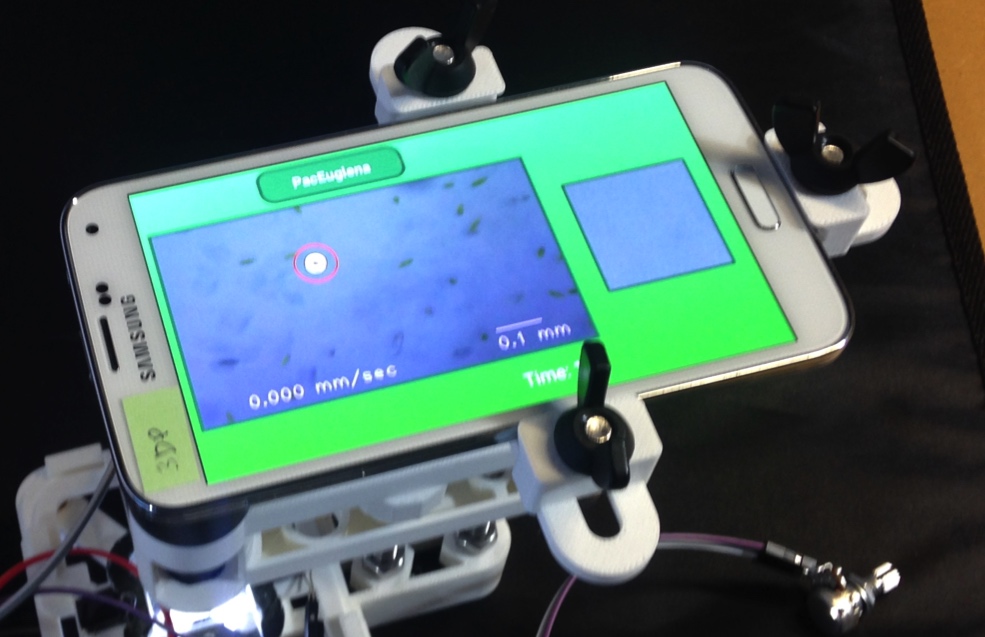

Supplement: S1 Note — (DOCX) [file pone.0162602.s007.docx]
