# Supplementary material for: LudusScope: Accessible Interactive Smartphone Microscopy for Life-Science Education
Source: PLoS One. 2016 Oct 5;11(10):e0162602. doi: 10.1371/journal.pone.0162602 (PMC5051900; doi:10.1371/journal.pone.0162602)
Supplement: S2 Note — (DOC) [file pone.0162602.s008.doc]

**Supplementary Note 2**

**Table of Parts**

| **Full LudusScope Kit Parts** | | | | | | |
| --- | --- | --- | --- | --- | --- | --- |
|  | **Name** | **Quantity** | **Cost Per Unit** | **Total Cost** | **Vendor** | **Vendor ID** |
| **Scope** | Illumination LED | 1 | $0.50 | $0.50 | Sparkfun | COM-11121 |
|  | 9v power outlet | 1 | $5.95 | $5.95 | Sparkfun | TOL-00298 |
|  | Barrel jack | 1 | $2.95 | $2.95 | Sparkfun | TOL-08734 |
|  | 10x 18mm eyepiece | 1 | $14.00 | $14.00 | eBay |  |
|  | CCTV lens (3.6mm) | 1 | $3.35 | $3.35 | M12 Lenses | PT-3620 |
|  | Optics Lockring | 1 | $1.25 | $1.25 | M12 Lenses | PT-LR001P |
|  | Potentiometer (10kohm) | 1 | $0.95 | $0.95 | Sparkfun | COM-09939 |
|  | 500 0hm 1/4 Watt resistor | 1 | N/A | N/A | MakerShed |  |
| **LED Joystick** | Directional LEDs | 4 | $0.95 | $3.80 | Sparkfun | COM-00531 |
|  | Joystick | 1 | $3.95 | $3.95 | Sparkfun | COM-09032 |
|  | Joystick Breakout Board | 1 | $1.95 | $1.95 | Sparkfun | BOB-09110 |
|  | Breadboard | 1 | $4.95 | $4.95 | Sparkfun | PRT-12002 |
|  | 100 0hm 1/2 Watt resistor | 2 | N/A | N/A |  |  |
|  | 10 ohm 1/4 Watt resistor | 4 | N/A | N/A |  |  |
| **Misc Hardware** | 1/4-20 nut | 6 | $3.31/100 | $0.20 | McMaster | 94895A029 |
|  | 1/4-20 bolt (20mm) | 3 | $3.41/10 | $1.02 | McMaster | 92185A540 |
|  | 1/4-20 bolt (100mm) | 4 | $8.39/10 | $3.36 | McMaster | 90044A131 |
|  | 1/4-20 wingnut | 3 | $7.90/50 | $0.47 | McMaster | 94924A600 |
| **Total** |  |  |  | $48.65 |  |  |

| **Full Kit: 3D Printed STL Files** | | | |
| --- | --- | --- | --- |
| **Folder** | **Filename (.stl)** | **Quantity** | **Reference** |
| **Scope\Base** | baseLEDHolder | 1 | A |
|  | baseLeg | 2 | B |
|  | handle | 1 | C |
|  | illuminationBase | 1 | D |
|  | lips | 8 | E |
|  | middleBase | 1 | F |
|  | sampleHolder | 1 | G |
|  | sideStand | 1 | H |
| **Scope\OpticsTube** | eyepieceSpacers | X* | I |
|  | scopeKnob | 1 | J |
|  | scopeTubeBottomHalf | 1 | K |
|  | scopeTubeTopHalf | 1 | L |
| **Scope\PhoneHolder** | phoneHolderLegs | 4 | M |
|  | phoneHolderSpacer | 2X* | N |
|  | universalPhoneHolder | 1 | O |
|  | universalPhoneHolderBase | 2 | P |
|  | universalPhoneHolderSlider | 3 | Q |

* quantity X means you need an arbitrary number of eyepieceSpacers, two times as many phoneHolderSpacers.


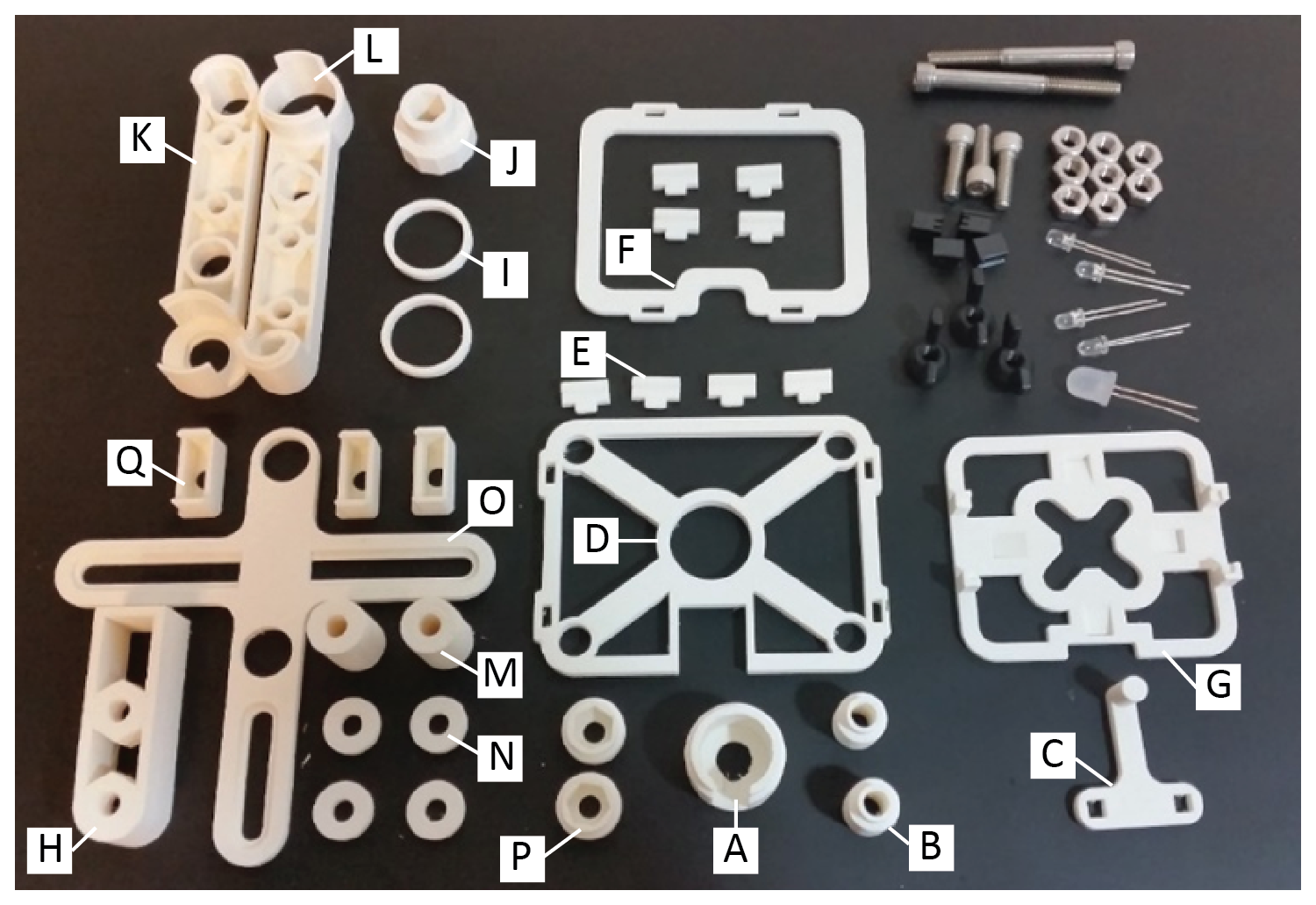


| **Sticker Microfluidic Chamber Parts** | | | | | | |
| --- | --- | --- | --- | --- | --- | --- |
|  | **Name** | **Quantity** | **Cost Per Unit** | **Yield** | **Vendor** | **Vendor ID** |
| **Sticker Microfluidic Chamber** | 1.5 mm acrylic sheet | 1 | $5.59/6”x6” | ~10 chips | McMaster | 8560K358 |
|  | Double-sided tape | 1 | $1.85/4”x8” | ~25 chips | Amazon |  |

| **Sticker Microfluidc: Laser Cutter .ai List** | | |
| --- | --- | --- |
| **Folder** | **Filename (.ai)** | **Quantity** |
| **LaserCutterFiles** | laserTape | 1 |
|  | laserAcrylic | 1 |

| **Microscope Attachment Parts** | | | | | | |
| --- | --- | --- | --- | --- | --- | --- |
|  | **Name** | **Quantity** | **Cost Per Unit** | **Total Cost** | **Vendor** | **Vendor ID** |
| **LED Joystick** | 9v power outlet | 1 | $5.95 | $5.95 | Sparkfun | TOL-00298 |
|  | Barrel jack | 1 | $2.95 | $2.95 | Sparkfun | TOL-08734 |
|  | Directional LEDs | 4 | $0.95 | $3.80 | Sparkfun | COM-00531 |
|  | Joystick | 1 | $3.95 | $3.95 | Sparkfun | COM-09032 |
|  | Joystick Breakout Board | 1 | $1.95 | $1.95 | Sparkfun | BOB-09110 |
|  | Breadboard | 1 | $4.95 | $4.95 | Sparkfun | PRT-12002 |
|  | 100 0hm 1/2 Watt resistor | 2 | N/A | N/A |  |  |
|  | 10 ohm 1/4 Watt resistor | 4 | N/A | N/A |  |  |
| **Misc Hardware** | 1/4-20 bolt (10mm) | 3 | $7.31/50 | $0.44 | McMaster | 92196A537 |
|  | 1/4-20 wingnut | 3 | $7.90/50 | $0.47 | McMaster | 94924A600 |
| **Total** |  |  |  | $23.55 |  |  |

| **Microscope Attachment: 3D Printed STL Files** | | |
| --- | --- | --- |
| **Folder** | **Filename (.stl)** | **Quantity** |
| **ScopeAttachment** | clampUniversal | 1 |
|  | scopeAttachSlider | 3 |
|  | scopeAttachUniversal | 1 |
| **Scope\Base** | sampleHolder | 1 |
