# Supplementary material for: LudusScope: Accessible Interactive Smartphone Microscopy for Life-Science Education
Source: PLoS One. 2016 Oct 5;11(10):e0162602. doi: 10.1371/journal.pone.0162602 (PMC5051900; doi:10.1371/journal.pone.0162602)
Supplement: S3 Note — (DOCX) [file pone.0162602.s009.docx]

**Supplementary Note 3**

**Minimal Worksheet Used for User Study**

Here we present a minimal worksheet, as used for the student user study presented in the paper. We began with a fifteen minute introduction to the operation of the LudusScope and the Euglena Soccer application. Finally, after the student was familiarized with the operation of the scope they spent 15 minutes answering the questions below while playing the game. These questions cover a basic set of qualitative and quantitative observations about the Euglena, and can serve as a starting point for more thorough worksheet tailored to the specific learning goals of the curricula.

**Worksheet:**

1. What color is the Euglena cell?
2. How long is a Euglena cell?
3. How fast does the cell move?
4. Do all cells have the same speed? Measure three different cells!
5. Do Euglena respond to light?
6. Do Euglena move to or away from the light?
7. Draw a Euglena cell!
