# Supplementary material for: LudusScope: Accessible Interactive Smartphone Microscopy for Life-Science Education
Source: PLoS One. 2016 Oct 5;11(10):e0162602. doi: 10.1371/journal.pone.0162602 (PMC5051900; doi:10.1371/journal.pone.0162602)
Supplement: S4 Note — (DOCX) [file pone.0162602.s010.docx]

**Supplementary Note 4**

**Links to External Files and Resources**

**Android code**

The Github link contains the Android and Arduino code for the LudusScope. The code is tested on a Samsung Galaxy S5.

<https://github.com/riedel-kruse-lab/biotic_games_android_sdk/>

**Scratch code**

The link below contains .sb2 files that can be opened with Scratch (<https://scratch.mit.edu/>). There are three separate files. The first Scratch file is a simulation of the *Euglena* turning experiment (*SI, Movie 7*). The second is a simulation of the *Euglena* soccer game (*SI, Movie 8*). The last is a simulation of an alternative *Euglena* Pacman-like game (*SI, Movie 9*). All use a model of *Euglena*  in which the organism turns in response to light stimuli equal to the sine of the difference of the swimming path and the direction of light, plus some noise parameter.

<http://web.stanford.edu/group/riedel-kruse/>

**STL and Illustrator files**

The link below contains all the files needed to construct the LudusScope. See SI, Appendix 2 for details o the files.

<http://web.stanford.edu/group/riedel-kruse/>
