# Supplementary material for: Correction: LudusScope: Accessible Interactive Smartphone Microscopy for Life-Science Education
Source: PLoS One. 2016 Dec 9;11(12):e0168053. doi: 10.1371/journal.pone.0168053 (PMC5148091; doi:10.1371/journal.pone.0168053)
Supplement: S4 Note — (DOCX) [file pone.0168053.s001.docx]

**Supplementary Note 4**

**Links to External Files and Resources**

**Android code**

The Github link contains the Android and Arduino code for the LudusScope. The code is tested on a Samsung Galaxy S5.

[https://github.com/riedel-kruse-lab/](https://github.com/riedel-kruse-lab/biotic_games_android_sdk/)

**Scratch code**

The link below contains .sb2 files that can be opened with Scratch (<https://scratch.mit.edu/>). There are two separate files. The first Scratch file is a simulation of the *Euglena* turning experiment (S6 Video). The second is a simulation of the *Euglena* soccer game (S7 Video). All use a model of *Euglena* in which the organism turns in response to light stimuli equal to the sine of the difference of the swimming path and the direction of light, plus some noise parameter.

[https://github.com/riedel-kruse-lab/](https://github.com/riedel-kruse-lab/biotic_games_android_sdk/)

**STL and Illustrator files**

The link below contains all the files needed to construct the LudusScope. See Supplementary Note 2 for details of the files.

<http://www.thingiverse.com/HIRKLab/designs>
